# Genome-wide burden and association analyses implicate copy number variations in asthma risk among children and young adults from Latin America

**DOI:** 10.1038/s41598-018-32837-w

**Published:** 2018-09-27

**Authors:** Pablo Oliveira, Gustavo N. O. Costa, Andresa K. A. Damasceno, Fernando P. Hartwig, George C. G. Barbosa, Camila A. Figueiredo, Rita de C. Ribeiro-Silva, Alexandre Pereira, M. Fernanda Lima-Costa, Fernanda S. Kehdy, Eduardo Tarazona-Santos, Bernardo L. Horta, Laura C. Rodrigues, Rosemeire L. Fiaccone, Maurício L. Barreto

**Affiliations:** 1 Institute of Collective Health, Federal University of Bahia, 40110-040 Salvador, Bahia Brazil; 2 Center for Data Integration and Knowledge for Health, Oswaldo Cruz Foundation, 41745-715 Salvador, Bahia Brazil; 3 Postgraduate Program in Epidemiology, Federal University of Pelotas, 464, 96020-220 Pelotas, Rio Grande do Sul Brazil; 4 Medical Research Council Integrative Epidemiology Unit, University of Bristol, Bristol, BS8 2BN United Kingdom; 5 Department of Statistics, Institute of Mathematics, Federal University of Bahia, 40170-110 Salvador, Bahia Brazil; 6 Institute of Health Sciences, Federal University of Bahia, 40110-100 Salvador, Bahia Brazil; 7 Nutrition School, Federal University of Bahia, 40110-150 Salvador, Bahia Brazil; 8 Heart Institute, University of São Paulo, 05403-900 São Paulo, São Paulo Brazil; 9 Rene Rachou Research Institute, Oswaldo Cruz Foundation, 30190-002 Belo Horizonte, Minas Gerais Brazil; 10 Leprosy Laboratory, Oswaldo Cruz Institute, Oswaldo Cruz Foundation, 21040-900 Rio de Janeiro, Rio de Janeiro Brazil; 11 Institute of Biological Sciences, Federal University of Minas Gerais, 31270-901 Belo Horizonte, Minas Gerais Brazil; 12 Department of Infectious Disease Epidemiology, Faculty of Epidemiology, London School of Hygiene and Tropical Medicine, London, WC1E 7HT UK

## Abstract

The genetic architecture of asthma was relatively well explored. However, some work remains in the field to improve our understanding on asthma genetics, especially in non-Caucasian populations and with regards to commonly neglected genetic variants, such as Copy Number Variations (CNVs). In the present study, we investigated the contribution of CNVs on asthma risk among Latin Americans. CNVs were inferred from SNP genotyping data. Genome wide burden and association analyses were conducted to evaluate the impact of CNVs on asthma outcome. We found no significant difference in the numbers of CNVs between asthmatics and non-asthmatics. Nevertheless, we found that CNVs are larger in patients then in healthy controls and that CNVs from cases intersect significantly more genes and regulatory elements. We also found that a deletion at 6p22.1 is associated with asthma symptoms in children from Salvador (Brazil) and in young adults from Pelotas (Brazil). To support our results, we conducted an *in silico* functional analysis and found that this deletion spans several regulatory elements, including two promoter elements active in lung cells. In conclusion, we found robust evidence that CNVs could contribute for asthma susceptibility. These results uncover a new perspective on the influence of genetic factors modulating asthma risk.

## Introduction

Asthma is a chronic inflammatory disorder of the airways characterized by reversible airflow obstruction. Asthma is clinically heterogeneous and patients may experience intermittent cough, dyspnea, wheezing, and chest tightness^1^. The pathophysiology of asthma is complex and typically involves airway eosinophilic inflammation, but many individuals can present a persistent noneosinophilic disease^2,3^. It is estimated that nearly 334 million people have asthma worldwide and its prevalence has been increasing in several regions of the planet^4,5^. In Latin America, the global prevalence of asthma symptoms in adolescents was estimated in approximately 16%^6^. Markedly, Brazil has one of the highest disease prevalence among Latin American countries, reaching 24,4% in 2002^7,8^.

The asthma epidemic observed in the last decades has been essentially attributed to temporal changes in a set of different factors, among them diet, allergen exposure, microbiota diversity and occurrence of infections that occurred particularly in high income countries and in urban areas of low-to-middle income countries^9–12^. Nevertheless, it is important to note that such changes in social and environmental conditions operate on individuals or populations with variable degrees of genetic predisposition to asthma. The initial studies mapping candidate genes in the context of asthma identified more than 200 genetic variants associated with disease development and severity, many of these associations being replicated in different populations^13,14^. Later, several large-scale studies, applying mainly small nucleotide polymorphism (SNP) microarrays and whole genome sequencing, have identified multiple short variants (rare and common) associated with asthma in different loci, including: 1q31.3 (*DENND1B*), 2q12.1 (*IL1RL1/IL18R1*), 5q12.1 (*PDE4D*), 5q22.1 (*TSLP/WDR36*), 5q31.1 (*IL13*), 6p21.32 (*HLA-DR/DQ*), 9p24.1 (*IL33*), 14q11.2 (*DAD1/OXAL1L*), 15q22.2 (*FOXB1*) and 17q21.1 (*ORMDL3*/*GSDMB*)^15–20^.

Due to all these efforts, the genetic architecture of asthma is now relatively well known, with the genetic factors identified so far explaining a reasonable proportion of the heritability attributed to the disease (varying between 35% and 95%)^21^. However, some work remains in the field to improve our understanding on asthma genetics, especially in non-Caucasian populations and with regards to other variants found in the human genome, such as Copy Number Variations (CNVs). CNVs are large deletions or duplications that can encompass genes (and their regulatory elements) leading to dosage imbalances^22^. Estimates suggest that CNVs affect approximately 12% of the human genome^23^. These structural variations have been widely studied in several complex human traits, including immunological disorders such as type 1 Diabetes^24,25^ and rheumatoid arthritis^24,26^. However, few comprehensive studies explored the role of CNVs in asthma and only suggestive associations have been found^27,28^. Additionally, it was observed that genes involved in asthma pathogenesis are affected by CNVs^29^.

Here, we conducted a genome wide copy number variation study based in our previously published SNP genotyping data^19^ to investigate the contribution of CNVs on asthma risk in Latin American admixed populations.

## Results

### Global contribution of copy number variations on asthma outcome

**Figure 1:**
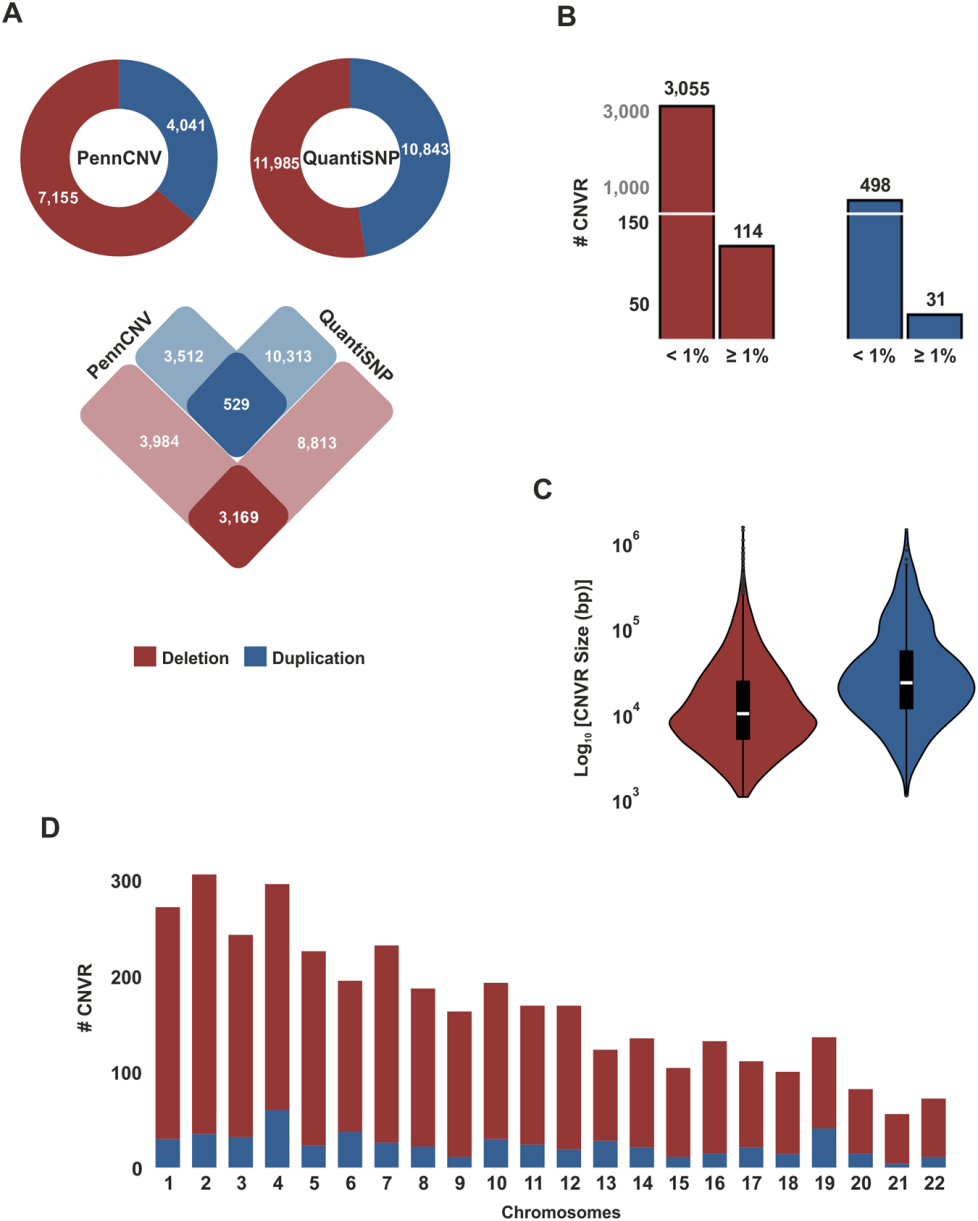
Copy number variation regions (CNVRs) detected in the genome of children from Salvador, Brazil. Copy number variations in the genome of 872 individuals from the SCAALA-Salvador cohort were inferred from SNP microarray data (Illumina HumanOmni 2.5–8v1 panel), using algorithms implemented in PennCNV v1.0.1 and QuantiSNP v2.0. Deletions or duplications showing sequence overlap were grouped into a single CNVR. (**A**) Number (#) of CNVRs detected by PennCNV and QuantiSNP (after quality control). Only CNVRs detected by both algorithms were considered for further analysis. (**B**) Frequency of deletions and duplications (<1% or ≥1%) in the study population. (**C**) Size of the deletions and duplications (bp, base pair), violin plots. (**D**) Number (#) of CNVRs by human autosomal chromosome.

Copy number variations in the genome of admixed children from Salvador (Northeast Region of Brazil) (Table 1) were inferred from SNP genotyping data (Illumina HumanOmni 2.5–8v1 panel) using two distinct algorithms implemented in PennCNV and QuantiSNP. To combine CNVs corresponding to the same event, deletions or duplications showing sequence overlap were grouped into a single copy number variation region (CNVR). Only CNVRs detected by both programs were considered valid. After stringent quality control (detailed in Methods), a set of 3,698 CNVRs (3,169 deletions and 529 duplications) was identified in 872 individuals (Fig. 1A). Of these, only 114 deletions and 31 duplications presented frequencies ≥ 1% in this study population (Fig. 1B and Supplementary Table 1). Regarding the median size of the CNVRs, it was found that duplications (22.0 kb) are more than twice as large as deletions (9.6 kb) (Fig. 1C). Finally, we found that CNVRs were well dispersed across the genome and the distribution of these events reflects the size of the human chromosomes, with decreasing frequency of CNVRs from the first to the twenty-second autosomal chromosome (Fig. 1D).

**Table 1:** Characteristics of the studied samples (after quality control).

Sample Characteristics	Salvador, Brazil						Pelotas, Brazil					
	Cases			Controls			Cases			Controls		
Number of individuals	188			684			367			1381		
Sex (male/female)	108/80			370/314			165/202			678/703		
Median age, years (IQR)	6 (4–11)			6 (4–10)			23 (22–23)			23 (22–23)		
LRRSD, median^a^	0.155			0.152			0.155			0.151		
Ancestry, median %^b^	**EUR**	**AFR**	**NAT**	**EUR**	**AFR**	**NAT**	**EUR**	**AFR**	**NAT**	**EUR**	**AFR**	**NAT**
	40.4	52.1	5.9	42.3	50.8	5.8	81.4	8.9	7.6	84.1	7.0	7.1

^a^Log_2_ of R ratio standard deviation.

^b^Ancestry proportions (ADMIXTURE method), EUR: European; AFR: African; NAT: Native American.

IQR, interquartile range.

After identifying CNVs in the genome of children from Salvador, analyses were conducted to evaluate the global impact of these structural variations on asthma outcome (Table 2). First, the number of CNVR per individual (CNVR count) was compared between patients and healthy controls and no significant difference was found. In average, it was observed 12.8 deletions and 3.6 duplications per asthmatic individual, while among non-asthmatic subjects we identified similar proportions, 14.3 deletions and 3.0 duplications per sample. Next, the size of CNVRs was compared between groups and it was found that structural variations (deletions + duplications) from asthmatic individuals are significantly larger than those presented by their controls (p = 5 × 10^−3^). The mean sizes of the deletions found in cases and controls were 35.6 kb and 26.4 kb, respectively (p = 0.03). Meanwhile, the average sizes of the duplications from cases and controls were 85.1 kb and 62.1 kb, respectively (p = 0.05). Based on this finding, we hypothesized that CNVRs from cases could mobilize more genes, regulatory and constrained elements than those from controls. To evaluate this assumption, CNVR positions were cross-referenced with DNA sequence annotations. As shown in Table 2, we found no significant differences regarding the number of constrained elements (sequence conservation across mammals) intersected by deletions and duplications from asthmatic and non-asthmatic individuals. On the other hand, CNVRs from cases mobilized significantly more genes (deletions, p = 0.01; duplications, p = 0.02; deletions + duplications, p = 2 × 10^−4^) and more regulatory elements (deletions, p = 0.03; duplications, p = 0.06; deletions + duplications, p = 7 × 10^−4^) than those from controls.

**Table 2:** Global contribution of copy number variation regions (CNVRs) on asthma outcome.

*Deletions*	Asthmatics					Non-asthmatics					Ratio	p
	Mean	SD	Median	P25	P75	Mean	SD	Median	P25	P75		
CNVR count^a^	12.8	11.2	10.5	8.0	14.0	14.3	18.0	10.0	8.0	14.0	0.9	0.8
Size (kb)^b^	35.6	94.3	11.3	5.1	28.2	26.4	63.2	9.9	5.0	24.3	1.3	**0.03**
Gene count^c^	1.1	2.0	1.0	0.0	2.0	0.8	1.6	1.0	0.0	1.0	1.4	**0.01**
Regulatory element count^d^	5.1	11.6	2.0	0.0	5.0	3.9	8.7	2.0	0.0	4.0	1.3	**0.03**
GERP element count^e^	6.1	8.1	3.9	0.5	8.0	6.5	8.6	4.2	0.7	8.7	0.9	0.1
***Duplications***												
CNVR count^a^	3.6	4.5	2.0	1.0	4.0	3.0	3.7	2.0	1.0	4.0	1.2	0.2
Size (kb)^b^	85.1	161.2	28.1	13.6	75.8	62.1	118.3	22.9	11.2	53.1	1.4	**0.05**
Gene count^c^	2.8	4.2	1.0	0.0	3.0	2.1	3.5	1.0	0.0	2.0	1.3	**0.02**
Regulatory element count^d^	14.0	22.4	5.0	2.0	15.0	11.4	19.7	4.0	1.0	12.0	1.2	0.06
GERP element count^e^	7.5	8.9	4.7	2.1	9.7	7.6	8.2	4.9	2.4	10.2	1.0	0.5
***Deletions*** + ***Duplications***												
CNVR count^a^	16.4	12.4	13.0	10.0	18.0	17.3	18.4	13.0	10.0	18.0	1.0	0.5
Size (kb)^b^	43.0	108.4	13.0	5.8	32.9	31.6	74.8	11.4	5.5	28.9	1.4	**5** × **10**^**−3**^
Gene count^c^	1.4	2.7	1.0	0.0	2.0	1.0	2.1	1.0	0.0	1.0	1.4	**2** × **10**^**−4**^
Regulatory element count^d^	6.8	14.8	2.0	0.0	7.0	5.0	11.5	2.0	0.0	5.0	1.4	**7** × **10**^**−4**^
GERP element count^e^	6.3	8.3	4.0	0.7	8.3	6.6	8.5	4.3	1.0	8.9	1.0	0.1

Asthmatic and non-asthmatic children from Salvador-SCAALA were compared in terms of: ^a^count of CNVRs per individual; ^b^estimated size of CNVRs (kb, kilobase); ^c^number of genes captured by CNVR; ^d^number of regulatory elements captured by CNVR; ^e^number of GERP constrained elements captured by CNVR.

SD: standard deviation; P25: 25% percentile; P75: 75% percentile; Ratio: case/ctrl ratio (mean); p values ≤ 0.05 were considered statistically significant [Mann-Whitney U test (two-sided)].

### Association of copy number variations with asthma in salvador

In the discovery association phase, analyses were conducted to evaluate the effect of specific structural variations on asthma risk in children from Salvador. The association of CNVRs with asthma was investigated by comparing frequencies of low-to-common variations (minor allele frequency ≥1%) between asthmatic and non-asthmatic individuals, under an additive model. Sex and age, which are considered classic risk factors for asthma, were included as covariates in the logistic regression analysis. Additionally, Log_2_ of R ratio standard deviation (LRRSD), to account for potential differences in sample and/or call quality between cases and controls, and the first three principal components, to correct for population stratification, were included in the regression model. This initial screening stage revealed several deletions and duplications that were nominally associated with asthma (p ≤ 0.05). Supplementary Table 2 shows the results for all CNVRs evaluated in the discovery study. Remarkably, only one deletion with approximately 41.6 kb of size, located at 6p22.1 (6:29,889,788–29,931,412) (Supplementary Fig. 2A), was significantly associated with the disease (OR = 3.0, p = 2 × 10^−4^) (Table 3), overcoming the significance level established for this discovery phase (p ≤ 3.4 × 10^−4^).

**Table 3:** A deletion region located in the locus 6p22.1 is associated with asthma in two independent Brazilian populations.

CNVR	CNVR class	CNVR size (bp)	Freq. Case	Freq. Ctrl	OR	95% CI	p	Power
**Salvador, Brazil (Discovery)**								
6:29,889,788–29,931,412	Deletion	41,624	6.6	2.4	3.0	1.7–5.2	**2 × 10** ^**−4**^	0.902
**Pelotas, Brazil (Replication)**								
6:29,881,842–29,931,412	Deletion	49,570	4.0	1.6	1.9	1.2–2.8	**4 × 10** ^**−3**^	0.837
				**Random-effects meta-analysis**				
						**OR**	**p**	
						2.3	**3 × 10** ^**−6**^	

CNVR: Copy number variation region, chromosome:start-end; bp: base pair; Frequency of CNVR (%); Case: asthmatic; Ctrl: non-asthmatic; OR, odds ratio; SE, SE of odds ratio (OR); 95% CI, 95% confidence interval; p, p value (additive model). The significance threshold established for the discovery phase was p ≤ 3.4 × 10^−4^; The significance level applied in the replication study was p = 0.05; Power: A posteriori statistical power.

Covariates in multivariate analysis: sex, age, Log_2_ of R ratio standard deviation (LRRSD) and principal components (PC1, PC2 and PC3).

Human genome assembly: GRCh38.

### Replication study and association in different ancestry compositions

We then attempted to replicate the association signal at 6p22.1 in another admixed Brazilian sample, composed of 1,748 young adults from the city of Pelotas, located in the Southern Region of Brazil (Table 1). CNVRs located in the locus 6p22.1 were also inferred from SNP genotyping data (Illumina HumanOmni 2.5–8v1 panel) using PennCNV and QuantiSNP. Interestingly, both algorithms detected a 49.6 kb deletion (6:29,881,842–29,931,412) (Supplementary Fig. 2B) whose limits overlap those of the deletion associated with asthma in Salvador, representing, therefore, a single CNVR. As show in Table 3, the association of this structural variation with asthma was replicated in this second Brazilian cohort (OR = 1.9, p = 4 × 10^−3^), with p value below the significance threshold assumed for the replication phase (p = 0.05).

Next, we conducted a meta-analysis on Salvador and Pelotas samples, by applying a random-effects model that assumes significant inter-study variability (Table 3). This analysis confirmed association of this deletion with the disease (OR = 2.3; p = 3 × 10^−6^), providing support for the notion that structural variations could represent risk factors for asthma.

Additional experiments were conducted to evaluate the effect of the deletion at 6p22.1 in subjects with different ancestry. First, our data sets were dichotomized in groups of individuals with proportion of European ancestry above or below the median. Next, we carried out association tests in these subgroups and, despite the reduced sample sizes, the deletion was nominally associated (p ≤ 0.05) with asthma in both situations (proportion of European ancestry above or below the median) (Supplementary Table 3).

### Fine-mapping of the 6p22.1 region

Considering that 6p22.1 is a very complex region, making association signals difficult to interpret, we performed a fine-mapping of the entire locus (6:27,100,000–30,500,000; RefSeq: GRCh38). We focused in the identification of SNPs that could explain the association signal found in this region (Supplementary Fig. 3). Notably, no robust linkage disequilibrium (r^2^ > 0.6) was found between our deletion and any evaluated SNP in the region. In addition, none of the SNPs investigated in this region was significantly associated with asthma risk in Salvador [locus p-value threshold = 8 × 10^−6^ (0.05/6057 SNPs)] and Pelotas [locus p-value threshold = 9 × 10^−6^ (0.05/5782 SNPs)]. We also carried out conditional tests to evaluate the possibility that our deletion and any other SNP tested could be capturing the same association signal. Remarkably, we found that association signals for the SNPs at 6p22.1 are not influenced by the signal of the reported deletion, i.e., the −log_10_ (p values) after adjustment for the deletion genotypes were strongly correlated with −log_10_ (p values) without adjustment [Pearson correlation: Salvador (r^2^ = 0.97; p-value < 10^−4^); Pelotas (r^2^ = 0.98; p-value < 10^−4^)].

### In silico functional analyses

**Figure 2:**
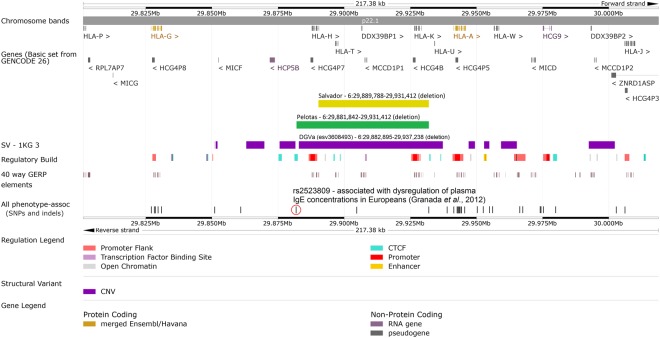
*In silico* functional study on the regulatory potential of the deletion at 6p22.1. Schematic representation of the locus containing the deletion region associated with asthma in Brazilian populations (expanded view: 6:29,801,381–30,018,756; RefSeq: GRCh38). This region was cross-referenced with DNA sequence annotations, including: location of protein coding or non-protein coding genes (GENCODE 26); presence of large structural variations identified by the 1000 genomes project, phase 3 (SV – 1KG 3); position of putative regulatory elements (regulatory build); location of constrained elements for 40 eutherian mammals (GERP, Genomic Evolutionary Rate Profiling); presence of SNPs and short indels associated with any human phenotype in previous studies. Limits of the deletions found in samples from Salvador or Pelotas are symbolized by a yellow or a green bar, respectively. Image created using the Ensembl genome browser (http://www.ensembl.org).

To investigate the regulatory potential of the deletion at 6p22.1, the region was cross-referenced with genomic and epigenomic annotations, obtained from the Ensembl database. This region was evaluated in terms of transcripts location, binding sites for transcription factors, sequence constraint, chromatin segmentation state (evidences of promoter and enhancer marks) and enrichment for marks of open chromatin (DNase I hypersensitive sites). In Fig. 2, it is possible to observe the limits found in Salvador and Pelotas for the asthma-associated deletion. This deletion region may have relevant functional consequences, since it covers a region with seven transcripts, numerous constrained sequences and several regulatory elements (including promoter and promoter flanking regions, transcription factor binding sites and an open chromatin element). In addition, it is close to HLA genes (*HLA-G* and *HLA-A*) and intersects a SNP (rs2523809) previously associated with dysregulation of plasma IgE concentrations in Europeans^30^. Collectively, these data support the biological plausibility of our findings.

## Discussion

Initially, we conducted an exploratory analysis, based in our previously published high-density SNP genotyping data^19^, to detect copy number variations throughout the genome of children from Salvador, Brazil. After stringent quality control, the algorithm implemented in PennCNV identified 7,155 deletions and 4,041 duplications, while QuantiSNP detected 11,985 deletions and 10,843 duplications, in 872 individuals. To avoid false discoveries, we focused only on the variations simultaneously detected by the two programs, remaining 3,169 deletions and 529 duplications. These results highlight an imbalanced ratio between the numbers of deletions and duplications. This can be explained primarily by limitations related to the PennCNV algorithm for the detection of duplication events, which are normally inferred by increased number of peaks in the BAF distribution, as well as increased LRR values. Wang and colleagues (2007)^31^ obtained similar results when testing the PennCNV package. In their data set, deletions were approximately twofold more frequent than duplications. Furthermore, they also found that deletions presented smaller sizes than duplications.

Then, we tested the hypothesis that the cumulative effect of multiple structural variations through an individual’s genome could increase asthma risk. Initially, we found no significant difference in the numbers of CNVRs between asthmatic and non-asthmatic individuals. Nevertheless, we found that CNVRs were larger in cases when compared to controls and that CNVRs from cases intersected significantly more genes and regulatory elements. Despite the modest differences found, this may be increasing the risk of presenting asthma symptoms. To date, the only genome-wide burden analysis associating asthma and CNVs found no evidences on the global contribution of these variations in disease risk^27^. However, it is important to note that this cited study was carried out among Australian children (European descent), using a less dense SNP chip (Illumina 610 K array). Besides that, their analyses were restricted to large (100 to 1.000 kb) and common CNVs [minor allele frequency (MAF) >5%]. In the present study, more robust conditions were created to detect the joint effect of structural variations on asthma risk by applying a much higher density SNP platform (with 2,237,482 SNPs) and by using broader spectra of CNV size (ranging from 1 to 1,430 kb) and frequency (rare to common). Similar results have already been described for other human traits, such as schizophrenia^32^ and obesity^33^.

Individual effects of CNVRs were also evaluated and we found that a deletion located at 6p22.1 was significantly associated with asthma symptoms in Salvador. The SCAALA-Salvador cohort has the largest proportion of African ancestry (50.8%) among the EPIGEN-Brazil populations^34^, with 42.9% and 6.4% of European and Native American ancestries, respectively. This association was replicated in another Brazilian admixed population from the EPIGEN-Brazil program, composed of young adults from the city of Pelotas. Global ancestry in Pelotas is 76.1% European, 15.9% African, and 8% Native American. Even though genetic ancestry at locus-segment level needs to be investigated, this consistent effect found in populations with different genetic backgrounds suggests functional relevance for this deletion or strong linkage to a causal variant yet to be identified.

Despite our sample size in the discovery study (188 asthma cases and 684 controls), our analysis was well powered (>80%) to detect the effect found for the deletion at 6p22.1 (OR = 3.0). This was possible because we evaluated only 145 low-to-common (MAF ≥ 1%) CNVRs that placed the significance level at 3.4 × 10^−4^. Even though not achieving statistical significance following adjustment for multiple tests, several other CNVRs were nominally associated (p ≤ 0.05) with asthma symptoms in the discovery phase (Supplementary Table 2). Although further studies using larger samples are necessary to confirm these results, we investigated if these nominal associations occurred in loci associated with asthma symptoms in our previous study^19^. Notably, no CNVRs were identified in the regions 14q11.2 (*DAD1/OXAL1L* genes) and 15q22.2 (*FOXB1* gene) that could explain SNP associations. Furthermore, we found no deletions or duplications nominally associated with asthma symptoms in loci consistently associated with the disease in previous studies, including: *DENND1B, IL1RL1, PDE4D, TSLP, IL13, HLA-DR/DQ* and *IL33* regions.

To support our results, we carried out an *in silico* functional analysis of the deletion at 6p22.1. Remarkably, this structural variation region was previously identified through DNA sequencing in populations from several continents by the 1000 genomes project, phase 3 (Del 6:29,882,895–29,937,238, RefSeq: GRCh38; DGVa ID: esv3608493). We evaluated several genomic annotations in this region and found that the sequence covered by the asthma-associated deletion spans essentially pseudogenes. Nevertheless, it deletes several regulatory elements in this region, including two promoters active in lung cells (empirical data from the ENCODE project)^35^ that could be involved in local gene expression regulation. Indeed, this deletion is located near the *HLA-A* and *HLA-G* genes and could impact on their transcriptional regulations. The HLA-A product, as a classical MHC I antigen, is responsible for initiating cell-mediated immunity^36^. On the other hand, HLA-G protein, a non-classical MHC I antigen, has immunoinhibitory functions and the loss of HLA-G immune-mediated control seems to be involved in the onset of inflammatory diseases^37^. Interestingly, Granada and colleagues (2012)^30^ found several SNPs near the *HLA-A* and *HLA-G* genes as potential determinants of atopy and IgE production among Europeans. In the aforementioned study, the SNP rs2523809, which is located at approximately 59 kb 5′ of the *HLA-A* gene and is intersected by our asthma-associated deletion, was strongly associated (4 × 10^−8^) with dysregulation of plasma IgE concentrations. Linkage disequilibrium between the SNP rs2523809 and the deletion at 6p22.1 was investigated in our cohorts and very low values were found (r^2^ < 0.1). Additionally, a recent meta-analysis identified another SNP (rs1233578) in the region 6p22.1 that was strongly associated with asthma risk in individuals from ethnically diverse populations^38^. This SNP is located more than 1 Mb away from the 5′ end of the CNV reported in our study and they are not in linkage disequilibrium in our cohorts (r^2^ < 0.2). In addition, the association of this SNP with asthma was not replicated in Salvador (p-value = 0.38) and in Pelotas (p-value = 0.42).

Another important aspect is that we identified a genetic variant that confers susceptibility to asthma in populations with very different ages: children from Salvador (4–11 years of age) and young adults from Pelotas (22–23 years of age). Asthma has various clinical phenotypes that are age-related^39^ and several evidences indicate that although some genetic variations can influence risk of both childhood and adult-onset asthma, other loci are exclusively associated to each group^18^. Although we cannot establish that the appearance of asthma symptoms in patients from Pelotas occurred in adult life, it is possible to affirm that the deletion at 6p22.1 is a genetic risk factor for current asthma in both age groups. Furthermore, phenotyping was conducted in the present study by using the phase II ISAAC questionnaire on asthma symptoms, a tool that has already been applied in hundreds of studies and has proved to be useful to determine asthma prevalence worldwide^40^. However, we did not distinguish atopic from non-atopic asthma. Considering that atopic asthma represents a minor proportion of the cases reported in Latin America^41^ and that the 6p22.1 locus is potentially involved in IgE response^30^, the associations found in our data sets may be underestimated by phenotypic heterogeneity.

In conclusion, we found robust evidence that CNVs could contribute for asthma susceptibility. More specifically and to the best of our knowledge, for the first time we identified a deletion that confers susceptibility to asthma in Latin American children and young adults. These results uncover a new perspective on the influence of genetic factors modulating asthma risk.

## Methods

### Study design and populations

#### Discovery cohort (Salvador)

As previously described^34^, the SCAALA-Salvador (Social Changes, Asthma and Allergy in Latin America) is one of the three population-based cohorts from the EPIGEN-Brazil initiative on population genomics and genetic epidemiology. Originally, the SCAALA-Salvador is a longitudinal study that comprises children living in Salvador (Bahia State), a city of approximately 3 million inhabitants in Northeastern Brazil. Further details on the original cohort and the procedures for collecting data are described by Barreto and colleagues^42^.

#### Replication cohort (Pelotas)

The replication of the association findings was conducted in a cohort of Brazilians from the city of Pelotas, Rio Grande do Sul State. Pelotas is located in the Southern region of Brazil with approximately 340,000 inhabitants. Throughout 1982, the three maternity hospitals in the city were visited daily and births were recorded, corresponding to 99.2% of all births in the city. The live-born infants whose families lived in the urban area constituted the original cohort. Further details on the Pelotas (1982) birth cohort can be seen in Victora and Barros^43^.

### Ethics statement and accordance with guidelines and regulations

The SCAALA-Salvador study was approved by the ethics committee of the Institute of Collective Health (ISC) of the Federal University of Bahia (UFBA). For the Pelotas project, the Ethical Review Board of the Federal University of Pelotas (UFPel) approved all phases of the study. Genotyping of individuals from both cohorts was approved by Brazil’s National Research Ethics Committee (CONEP), as part of the EPIGEN-Brazil project (resolution number: 15895). Informed consent was obtained from all participants at baseline and at all follow-up interviews. Participants signed an informed consent form and authorized their genotyping. All methods and protocols were performed in accordance with the principles of the Declaration of Helsinki.

### Definition of asthma symptoms

Definition of asthma symptoms and phenotyping were performed in the same way for both discovery (Salvador) and replication (Pelotas) studies. Parents or caregivers of children from Salvador (resurveyed in 2005, 4–11 years of age) and young adults from Pelotas (resurveyed in 2004, 22–23 years of age) answered Portuguese-adapted questionnaires from The International Study of Asthma and Allergies in Childhood (ISAAC) project^40^. The interviews were carried out by appropriately trained researchers and individuals were classified as asthmatic when wheezing was reported in the 12 months prior to the questionnaire application and by reporting any one of the following situations: (1) diagnosis of asthma ever; (2) wheezing during exercise in the last 12 months; (3) four or more episodes of wheezing in the last 12 months; or (4) waking up at night because of wheezing in the last 12 months. All other individuals were classified as current non-asthmatics.

### SNP genotyping and quality control

Procedures for SNP genotyping and quality control (QC) were extensively described in Kehdy *et al*.^44^. Briefly, 1,307 children from Salvador and 1,841 young adults from Pelotas, who fully answered the asthma survey, were successfully genotyped as part of the EPIGEN-Brazil project using the Illumina HumanOmni 2.5–8v1 BeadChip panel (comprising 2,237,482 autosomal SNPs; Illumina, San Diego, CA). Stringent post-genotyping QC procedures and filtering were performed for both populations separately and 1 individual from Salvador and 20 from Pelotas were excluded due to inconsistency between the sex registered and the genetic sex, based on X-chromosome markers (using PLINK v1.9^45^; –check-sex). Fifty seven samples from Salvador and 71 from Pelotas were eliminated from further analysis because of close relationship estimated by kinship coefficients for each pair of individuals, using a method implemented in the REAP software (Relatedness Estimation in Admixed Populations)^46^. Pairs of individuals were considered closely related if the estimated kinship coefficient between them was ≥0.1. Finally, we eliminated 1 individual from Salvador and 2 from Pelotas presenting more than 1% of undetermined genotypes, using PLINK v1.9 (−mind 0.01). QC was also performed to eliminate autosomal SNPs showing significant deviation from the Hardy-Weinberg equilibrium [p < 10^−3^ (−hwe 0.001), based on controls only; 56,496 in Salvador and 82,307 in Pelotas] and SNPs with more than 1% of undetermined genotypes (−geno 0.01) in Salvador (112,230) and in Pelotas (99,419). These last two QC stages were also carried out using PLINK v1.9.

### Copy number variation calling and quality control

Intensity values from autosomal SNP probes that passed SNP QC were used to detect genomic structural variations based on algorithms implemented in two of the most used programs in the literature for the detection of copy number variations from SNP arrays: PennCNV v1.0.1^31^ and QuantiSNP v2.0^47^. Both PennCNV and QuantiSNP evaluate deviations in signal intensity patterns to identify changes in number of copies of DNA segments.

Two intensity values were obtained for each probe (using Genome Studio software v2011.1): LRR (Log_2_ of R ratio, where R is the value of the total intensity for the two SNP alleles) and BAF (B allele frequency, a measure of allelic intensity ratio for each SNP). Intensity values were quantile-normalized in order to avoid batch effects. SNP arrays may show variations in hybridization intensity. An algorithm described by Diskin and colleagues^48^ and implemented in PennCNV (genomic_wave.pl option; -adjust argument) was applied to adjust signal intensity values from samples showing a waveness factor (WF value) less than -0.04 or higher than 0.04.

To limit the occurrence of false discoveries in the initial phase, only CNVs ≥ 1 kb and overlapping at least 5 SNP probes were taken into account^49^. Considering that telomeric and centromeric regions show excessive spurious CNV calls^31^, CNVs with at least 1 bp (base pair) overlap with centromeric or telomeric regions (500 kb+/−) were not included in our analyses. Additionally, in MHC region (6:28,510,120–33,480,577, RefSeq: GRCh38), a highly repetitive locus, CNV calls with greater than 70% repeat coverage were excluded. RepeatMasker software (v4.0.6; default options) was used to screen interspersed repeats and low complexity DNA sequences. Following the QC procedures, 235 samples from Salvador were excluded on the basis of large variation in LRR intensities at genome-wide level [standard deviation (SD) >0.20]. Also, 141 samples from Salvador were eliminated from further analysis due to large number of CNVs called (2 SD from the mean) or large CNV sizes (2 SD from the mean). This CNV-based genomic QC was not applied to the Pelotas cohort, since analysis in the replication stage was restricted to the 6p22.1 region.

### Definition of copy number variation regions (CNVRs)

In order to combine structural variations corresponding to the same event, the duplications or deletions detected in the genome of the individuals were grouped into copy number variation regions (CNVRs). CNVs overlapping at least 1 base-pair were merged into a single CNVR^50^, using CNVRuler software^51^. To avoid overestimation of CNVR size and frequency, regional density (recurrence) of participating CNVs were checked and sparse areas not satisfying the density threshold (10%) were trimmed. Only CNVRs called by both PennCNV and QuantiSNP were considered valid.

### Sequence annotations

The regulatory potential of CNVs associated with asthma was evaluated *in silico*. Comparative genomic data and regulatory features for the region 6:29,881,842–29,931,412 (RefSeq: GRCh38) were obtained from the Ensembl database (http://www.ensembl.org). The position of the deletion at 6p22.1 was cross-referenced with DNA sequence annotations, including: (1) transcripts location (introns, exons, 3′ and 5′ untranslated regions); (2) presence of consensus sequences for transcription factors; (3) genomic evolutionary rate profiling–constrained elements for 40 eutherian mammals (GERP)^52^; (4) chromatin segmentation state^35^; and (5) indicative of chromatin accessibility (DNase I hypersensitive sites)^35^.

### Population structure analyses

To explore the admixed nature of our samples, we conducted principal components analysis (PCA) of ancestry, using PLINK v1.9. In Salvador (Supplementary Fig. 1A,B) and Pelotas (Supplementary Fig. 1C,D), only the first three principal components (PCs) account each one for more than 2% of data variance. So, these three more informative PCs were used to adjust for population stratification in the association tests. Additionally, the ADMIXTURE method^53^ was applied to dissect the ancestry composition of asthma cases and controls (Table 1). Based on the results of ADMIXTURE with number of ancestral clusters (K) = 3, we were able to differentiate the main continental parental groups that contributed to the formation of the Brazilian population: Europeans, Africans and Native Americans. These analyses were previously detailed in Kehdy *et al*.^44^.

### Statistical analysis

#### Burden analysis

Burden analyses were conducted to evaluate the global impact of CNVs on asthma outcome. Cases and controls from the discovery cohort were compared in terms of: (1) number of CNVRs per individual (CNVR count); (2) estimated size of CNVRs; (3) number of genes overlapped by a CNVR (at least 1 bp overlapped with any genic region); (4) number of regulatory regions overlapped by a CNVR (at least 1 bp overlapped with regulatory elements: promoter and promoter flanking region, enhancer, open chromatin and transcription factor binding site); and (5) number of constrained elements captured by a CNVR (at least 1 bp overlapped with GERP elements). Size of CNVRs and number of genes, regulatory and constrained regions covered by CNVRs are related to the total for all CNVRs per individual. Gene, regulatory and constrained element annotations were obtained from the Ensembl Biomart tool (http://www.ensembl.org/biomart; Ensembl Genes 88, RefSeq: GRCh38). All comparisons were performed with the non-parametric Mann-Whitney U test (two-sided), using SPSS statistics software v20.0 (IBM). Significance level used in this analysis was α = 0.05.

#### Association analysis

CNVRs were defined as low-to-common if their frequencies were ≥1% in our cohorts (cases and controls) and only low-to-common variants were evaluated at this point. For the discovery and replication phases, association of CNVRs with asthma risk was evaluated using PLINK v1.9. Distribution of genomic copy number segments was compared between cases and controls under an additive genetic model (0, 1 or 2 allele copies for deletions; 2, 3 or 4 allele copies for duplications). No CNVR with 5 or more allele copies has passed CNV-based QC. Classical risk factors for asthma, such as sex and age, were included as covariates from the logistic regression model. In addition, Log_2_ of R ratio standard deviation (LRRSD), to account for potential differences in sample and/or call quality between cases and controls, and the first three principal components from PCA (Supplementary Fig, 1A,C), to correct for eventual population stratification, were included in the regression model. Results are described as estimates of odds ratio (OR) and confidence interval (CI). In the discovery phase, a multiple test threshold (Bonferroni) was applied to the p values to control the probability of observing false-positive results. After that, p values ≤ 3.4 × 10^−4^ (0.05/145) were taken as significant. In the replication study, since only one CNVR was tested, the significance level was α = 0.05. To combine the association results found in both cohorts, a random-effects meta-analysis (assuming inter-study variability) was carried out using PLINK v1.9. A posteriori statistical power was estimated using the GAS Power Calculator tool. Linkage disequilibrium calculations (*r*^2^) were conducted using PLINK v1.9. Pearson correlations were carried out using SPSS statistics software v20.0.

## Electronic supplementary material

Supplementary Information
