# Supplementary material for: Genome-wide burden and association analyses implicate copy number variations in asthma risk among children and young adults from Latin America
Source: Sci Rep. 2018 Sep 27;8:14475. doi: 10.1038/s41598-018-32837-w (PMC6160443; doi:10.1038/s41598-018-32837-w)
Supplement: Supplementary file 1 — Supplementary Information [file 41598_2018_32837_MOESM1_ESM.pdf]

Genome-wide burden and association analyses implicate copy number variations in asthma risk among children and young adults from Latin America

Pablo Oliveira, Gustavo N. O. Costa, Andresa K. A. Damasceno, Fernando P. Hartwig, George C. G. Barbosa, Camila A. Figueiredo, Rita de C. Ribeiro-Silva, Alexandre Pereira, M. Fernanda Lima-Costa, Fernanda S. Kehdy, Eduardo Tarazona-Santos, Bernardo L. Horta, Laura C. Rodrigues, Rosemeire L. Fiaccone, Maurício L. Barreto

Content

Supplementary Figure 1 3

Supplementary Figure 2 4

Supplementary Figure 3 5

Supplementary Table 1 6

Supplementary Table 2 87

Supplementary Table 3 91

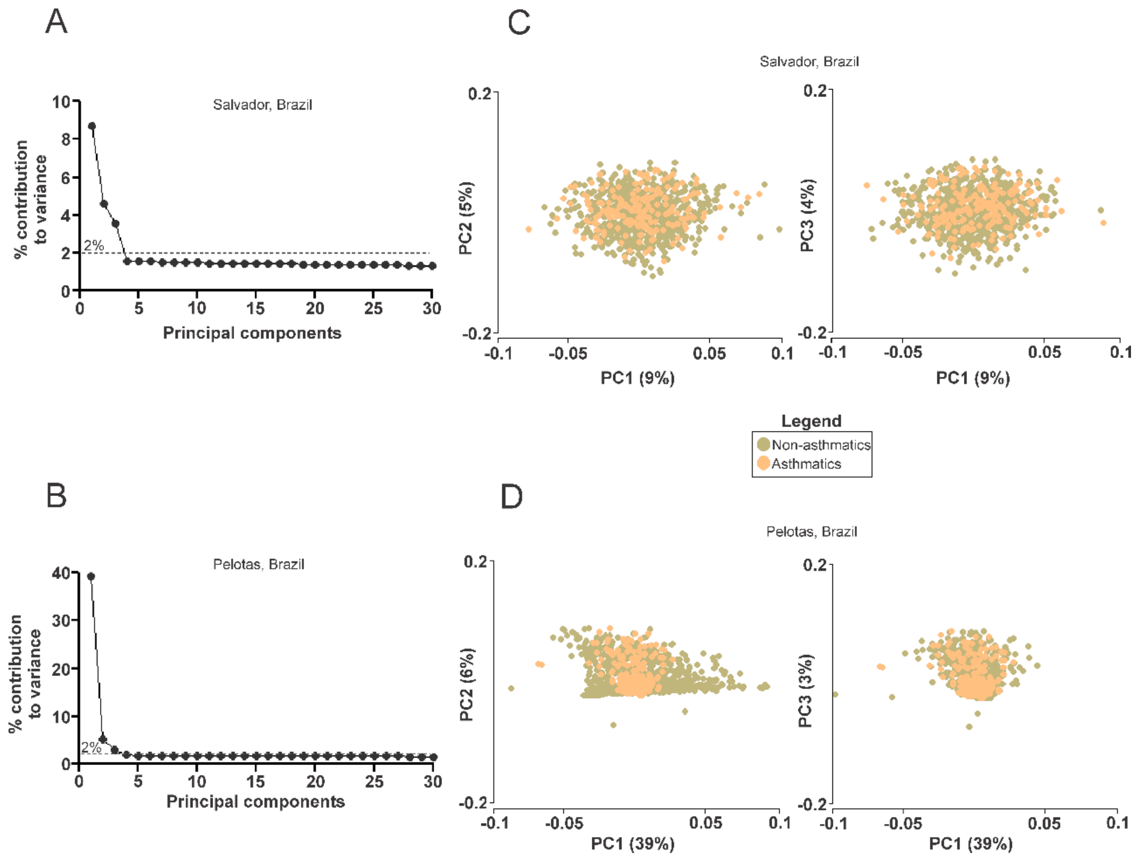

Supplementary Figure 1. Principal components analysis (PCA) of the two cohorts analyzed in this study. Scree plots showing the % of contribution to data variance for the first 30 principal components (PCs) in Salvador (**A**) and Pelotas (**B**). In both cohorts, the eigenvalues for the first 3 PCs are the only greater than 2%. 2D dot plots of the most informative PCs (PC1 vs PC2 and PC1 vs PC3) in Salvador (**C**) and Pelotas (**D**).

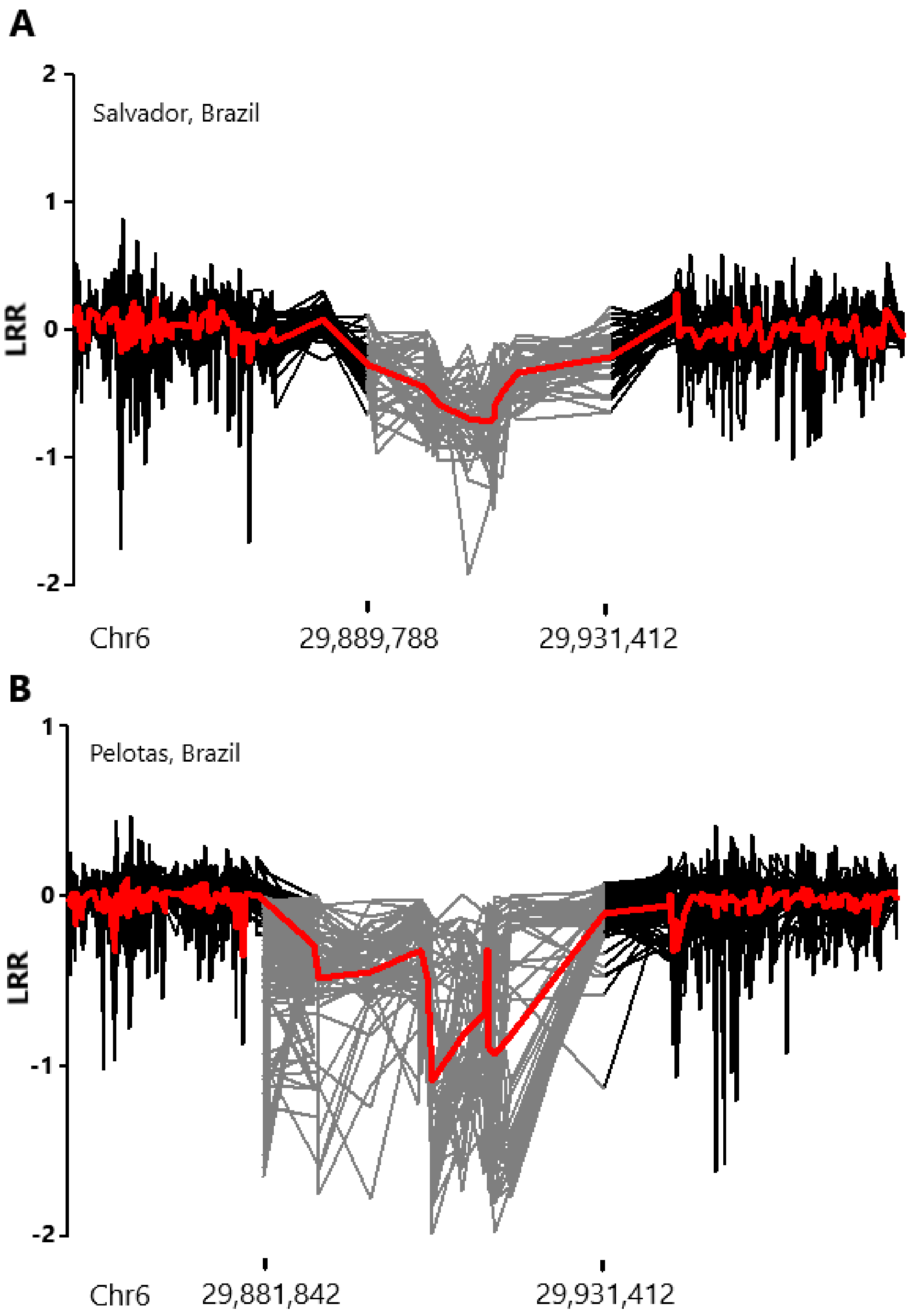

Supplementary Figure 2. Copy number variation region (CNVR) identified at 6p22.1. Log_2 of R ratio (LRR) of individuals carrying the 6p22.1 deletion in Salvador (**A**) and Pelotas (**B**). Red line: mean LRR from individuals carrying the CNVR. Human genome assembly: GRCh38.

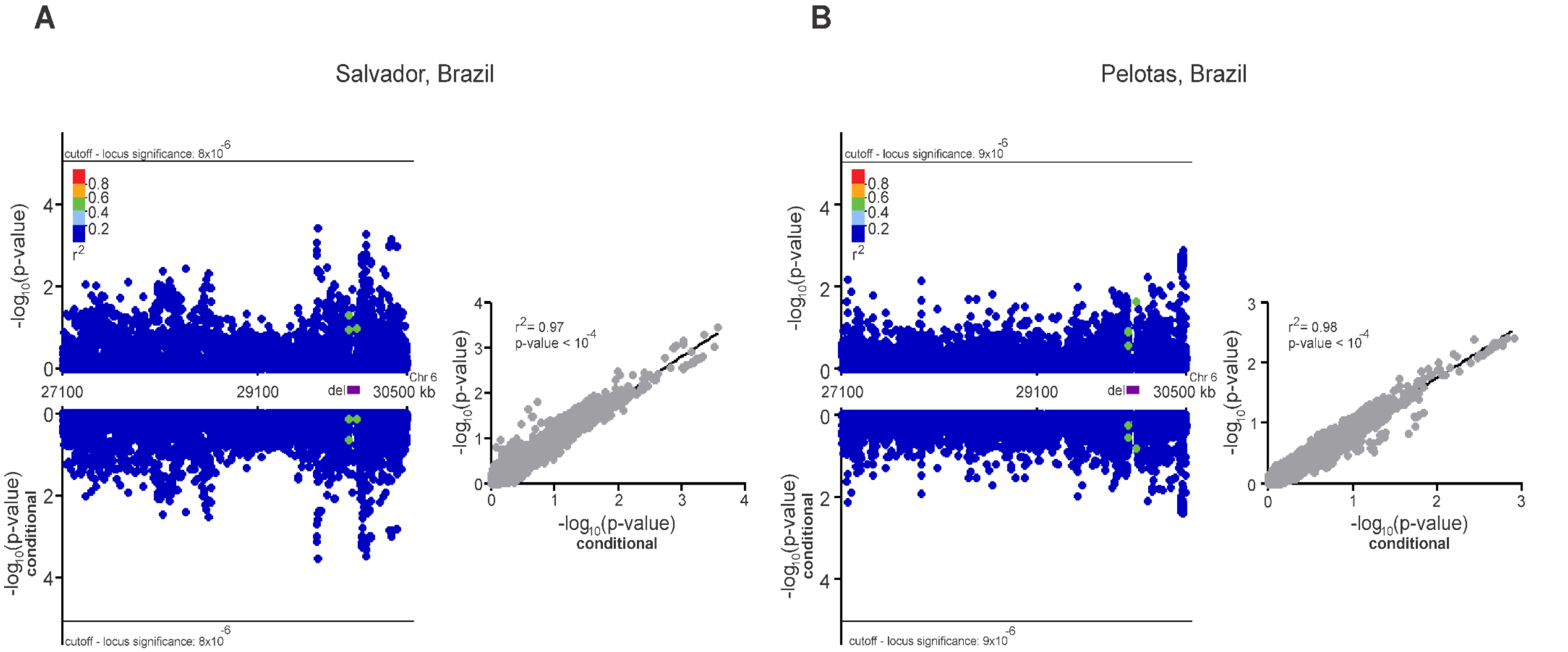

Supplementary Figure 3. Fine-mapping of the 6p22.1 locus. SNPs (Illumina HumanOmni 2.5-8v1) in the entire 6p22.1 region (6:27,100,000-30,500,000; RefSeq: GRCh38) were evaluated. The association of SNPs with asthma was investigated by logistic regression, under an additive model. Sex, age and principal components (PC1, PC2 and PC3) were included in the regression model as covariates [results are shown as $-\log_{10}$ (p-value)]. In the conditional analysis, deletion genotype was used as an additional covariate [results are shown as $-\log_{10}$ (p-value) conditional]. Locus p-value thresholds are represented by 0.05/number of SNPs tested. In Salvador (**A**) and Pelotas (**B**), 6057 and 5782 SNPs (after quality control) were investigated, respectively. Pearson linear correlation between $-\log_{10}$ (p values) adjusted for deletion genotypes and $-\log_{10}$ (p values) without adjustment were conducted. The limits of the deletion associated with asthma are symbolized by purple bars. Linkage disequilibrium (r^2) between the deletion and any SNP in the locus was also investigated.

Supplementary Table 1. Frequent copy number variation regions detected in individuals from Salvador-SCAALA by PennCNV and QuantiSNP

Chr	Start	End	Size (bp)	Frequency (%)
Duplications				
1	6416226	6512391	96165	0.92
1	19439832	19472431	32599	0.06
1	38301463	38318319	16856	0.06
1	43348396	43378597	30201	0.57
1	62175420	62201295	25875	0.06
1	66917226	66950709	33483	0.06
1	67546571	67555819	9248	0.06
1	69412199	69429633	17434	0.06
1	69649654	69666252	16598	0.06
1	77653328	77723536	70208	0.17
1	86037699	86161554	123855	0.06
1	108012816	108017831	5015	0.06
1	147295411	147320234	24823	0.06
1	151369094	151378584	9490	0.06
1	154945225	155087375	142150	1.09
1	159741082	159758934	17852	0.06
1	167226887	167235272	8385	0.06
1	182998334	183010425	12091	0.06
1	191800041	191847632	47591	0.34
1	197850936	197909571	58635	0.06
1	199181530	199189581	8051	0.06
1	211989059	212026247	37188	0.17
1	221713474	221720389	6915	0.06
1	223454297	223468098	13801	0.23
1	232652148	232701588	49440	0.06
1	236960447	236971011	10564	0.23
1	241382386	241385280	2894	0.06
1	241958238	241971884	13646	0.11
1	247664434	248272529	608095	0.52
1	248520558	248526493	5935	0.29
2	555176	720238	165062	0.17
2	796046	911633	115587	0.06
2	18150562	18161979	11417	0.06
2	24632070	24655342	23272	0.06
2	35177521	35183708	6187	0.06
2	35452014	35492456	40442	0.11
2	49460521	49465782	5261	0.23
2	56492665	56519292	26627	0.52
2	56714792	57107909	393117	0.06
2	74752363	74770617	18254	0.06
2	74919394	74997740	78346	0.11
2	76882001	76892220	10219	0.06

2	97535697	97674747	139050	0.34
2	107211485	107246922	35437	0.11
2	123386803	123421929	35126	0.06
2	132000728	132297217	296489	7.34
2	141651040	141763897	112857	0.06
2	154772998	154793467	20469	0.06
2	156611609	156613750	2141	0.11
2	158277999	158326961	48962	0.06
2	162976516	162995345	18829	0.06
2	164493392	164499833	6441	0.06
2	168963314	168969675	6361	0.06
2	169696721	169700840	4119	0.11
2	173957922	173980139	22217	0.06
2	194844685	194873302	28617	0.06
2	203899887	203910307	10420	0.34
2	204736295	204762413	26118	0.06
2	206799531	206804338	4807	0.11
2	210463939	210493961	30022	0.06
2	221991853	222020580	28727	0.06
2	227376905	227394586	17681	2.01
2	227581134	227918547	337413	0.06
2	229765975	229865828	99853	0.06
2	236848561	236849614	1053	0.06
3	576158	1347433	771275	0.17
3	1779529	1798009	18480	0.11
3	2531309	2533022	1713	0.06
3	6277212	6300723	23511	0.52
3	8783940	8813413	29473	0.11
3	17692492	17721460	28968	0.23
3	21210675	21300928	90253	0.11
3	21937094	21952767	15673	0.11
3	30414431	30428535	14104	0.06
3	35127025	35137093	10068	0.06
3	35765313	35899951	134638	0.06
3	36089642	36103577	13935	0.06
3	48779292	48816040	36748	0.23
3	57977845	57992829	14984	0.06
3	63075679	63243596	167917	0.11
3	75370585	75891946	521361	5.96
3	95437291	95548133	110842	2.18
3	110393694	110402099	8405	0.06
3	121631600	121634407	2807	0.06
3	130722331	130730404	8073	0.11
3	136471843	136522088	50245	0.06
3	144363555	144369423	5868	0.06
3	146674130	147058474	384344	0.23

3	148051270	148074957	23687	0.06
3	155761934	155787808	25874	0.34
3	164283490	164410087	126597	2.41
3	165542464	165618099	75635	0.69
3	167397263	167442503	45240	0.06
3	175008726	175020005	11279	0.06
3	177522405	177572673	50268	0.06
3	186827747	186940720	112973	0.06
3	186963419	186967199	3780	0.06
4	3041175	3068437	27262	0.17
4	11871409	11874497	3088	0.11
4	11909201	11925154	15953	0.06
4	16373541	16399452	25911	0.06
4	16615927	16626456	10529	0.06
4	25623528	25629772	6244	0.11
4	25844609	25923235	78626	0.06
4	27261988	27399723	137735	0.06
4	27418796	27471194	52398	0.06
4	27959911	27964883	4972	0.63
4	30568342	30610404	42062	0.06
4	31037291	31051910	14619	0.06
4	31791952	31895086	103134	1.38
4	35185290	35203790	18500	0.11
4	39496873	39514191	17318	0.23
4	40471342	40885974	414632	0.11
4	56197963	56205474	7511	0.06
4	57181361	57234479	53118	0.34
4	57865770	57883452	17682	0.06
4	58284430	58290372	5942	0.11
4	59627752	59643935	16183	0.06
4	60044718	60139651	94933	0.69
4	60197644	60221353	23709	0.23
4	60274324	60325984	51660	0.06
4	60365833	60390997	25164	0.06
4	64458635	64562461	103826	0.46
4	65733418	65764047	30629	0.06
4	68812961	68850046	37085	1.03
4	72758411	72764795	6384	0.11
4	79974477	79987274	12797	0.06
4	88262042	88311420	49378	0.06
4	97075057	97115868	40811	0.06
4	101731191	101750836	19645	0.34
4	115661574	115781230	119656	0.23
4	116393940	116461263	67323	0.06
4	117394404	117411847	17443	0.75
4	127927521	128124962	197441	0.52

4	129078426	130419497	1341071	0.40
4	130774276	130820154	45878	0.34
4	135055091	135205415	150324	0.46
4	135420989	135550966	129977	0.52
4	136720244	136753019	32775	0.06
4	137013387	137024311	10924	0.11
4	137386276	137412504	26228	0.17
4	158302182	158499327	197145	0.06
4	161031076	161086234	55158	0.23
4	163762897	163805955	43058	0.11
4	167039754	167332969	293215	0.06
4	167988321	168010230	21909	0.06
4	171403183	171443288	40105	0.11
4	171807843	171818519	10676	0.06
4	172292112	172326505	34393	0.06
4	174696163	174724266	28103	1.43
4	176212850	176217893	5043	0.69
4	176712142	176733309	21167	0.06
4	178086144	178119263	33119	0.23
4	178174163	178184294	10131	0.06
4	180348095	180358015	9920	0.40
4	183498299	183512278	13979	0.06
4	189309576	189413352	103776	0.40
5	2080480	2091624	11144	0.11
5	10120128	10175050	54922	0.06
5	13791861	14184007	392146	0.23
5	18359864	18360974	1110	0.52
5	18996857	19012175	15318	0.06
5	24143856	24150242	6386	0.34
5	27712909	27727704	14795	0.06
5	32658732	32694836	36104	0.06
5	41873629	42118462	244833	0.11
5	79082720	79094089	11369	0.06
5	84790666	84803174	12508	1.43
5	95886431	95939597	53166	0.06
5	99862563	100002726	140163	0.06
5	100266063	100367400	101337	0.17
5	110123833	110151360	27527	0.06
5	118344592	118431649	87057	0.11
5	119203647	119225667	22020	0.06
5	120962846	121079439	116593	0.23
5	126163417	126171614	8197	0.06
5	130139143	130368109	228966	0.11
5	131176798	131201384	24586	0.40
5	141350643	141360493	9850	0.11
5	145300348	145333952	33604	0.29

6	11574107	11577339	3232	0.06
6	19556284	19560023	3739	0.06
6	23799565	23803993	4428	0.11
6	29124213	29191852	67639	0.11
6	32253451	32257051	3600	0.23
6	32624423	32627111	2688	0.06
6	34213903	34265377	51474	1.66
6	48700193	48715904	15711	0.40
6	63293027	63314119	21092	0.06
6	67526541	67540959	14418	0.11
6	72302505	72312331	9826	0.06
6	73324884	73364162	39278	0.23
6	74696158	74729452	33294	0.06
6	83239302	83250620	11318	0.11
6	89146781	89165815	19034	0.17
6	93244935	93258133	13198	0.11
6	93292953	93301411	8458	0.06
6	95492640	95521115	28475	0.57
6	96921179	96929696	8517	0.06
6	98861181	99352825	491644	0.06
6	102118676	102155138	36462	0.06
6	103595583	103650272	54689	0.06
6	105176995	105192900	15905	0.06
6	110795516	111022191	226675	0.69
6	111113606	111335982	222376	0.11
6	117187918	117244290	56372	0.06
6	120624604	120662464	37860	0.17
6	121041290	121050457	9167	0.06
6	141988984	142010944	21960	0.29
6	149314865	149325172	10307	0.06
6	158688120	158706485	18365	0.06
6	165464769	165480063	15294	0.34
6	167198269	167268035	69766	0.23
6	167411159	167432533	21374	0.06
6	167933559	168197136	263577	0.92
6	168590686	168598990	8304	0.17
7	5049397	5072477	23080	0.63
7	9574931	9588021	13090	0.80
7	9859197	9928557	69360	0.06
7	13468915	13471981	3066	0.06
7	14686947	15579357	892410	0.40
7	20769922	20796794	26872	0.57
7	26042751	26091467	48716	0.06
7	26185559	26224658	39099	2.12
7	33092926	33147667	54741	0.06
7	37892348	37896928	4580	0.17

7	41136432	41145303	8871	0.06
7	45761447	45957771	196324	0.06
7	47368291	47678915	310624	0.17
7	56419507	56505640	86133	0.11
7	56517704	56591620	73916	0.06
7	62744672	63252790	508118	3.33
7	64435731	64467431	31700	0.06
7	67349913	67404013	54100	0.06
7	77665185	77672742	7557	0.06
7	82875907	82903347	27440	0.11
7	89837368	89863026	25658	0.11
7	97748769	98123642	374873	0.06
7	123153128	123248429	95301	0.17
7	140510141	140533785	23644	0.11
7	148932499	148951045	18546	0.17
7	152842286	152868926	26640	0.06
8	8921818	8933070	11252	0.06
8	11890825	11895723	4898	0.06
8	15562268	15575144	12876	0.11
8	16734050	16737245	3195	0.06
8	22355111	22383225	28114	0.11
8	26476982	26486298	9316	0.06
8	57298249	57311794	13545	0.06
8	57363838	57760353	396515	0.06
8	60644018	60655651	11633	0.06
8	61794805	61811611	16806	0.06
8	66560409	66575362	14953	0.06
8	66580106	66659086	78980	0.06
8	74570724	74593900	23176	0.06
8	86187173	86322952	135779	0.06
8	89516616	89534750	18134	0.46
8	89600644	89705854	105210	0.06
8	102806069	102822395	16326	0.06
8	111618105	111631233	13128	0.06
8	111697665	111734593	36928	0.29
8	123247191	123377368	130177	0.11
8	131986197	132038653	52456	0.11
8	133064115	133078740	14625	0.06
9	20840839	20949160	108321	0.06
9	27194391	27293011	98620	0.17
9	28458251	28475534	17283	0.06
9	32716890	32728906	12016	0.06
9	71559826	71848520	288694	0.06
9	74908391	74930322	21931	0.06
9	81867074	81888033	20959	0.06
9	104041268	104051184	9916	0.11

9	110157008	110175898	18890	0.11
9	113119384	113131637	12253	0.06
9	116762076	116771458	9382	0.40
10	5009340	5233115	223775	0.11
10	6635805	6796537	160732	0.11
10	7555151	7580143	24992	0.06
10	11651167	11664894	13727	0.11
10	11738325	11753602	15277	0.06
10	12500530	12509057	8527	0.46
10	16519909	16574606	54697	0.11
10	17581565	17595621	14056	0.17
10	20543783	20555017	11234	0.06
10	26581325	26620398	39073	0.11
10	29708817	29712504	3687	0.11
10	33566200	33576948	10748	0.06
10	42711252	42754262	43010	0.29
10	47293674	47306686	13012	0.06
10	52924457	52933583	9126	0.06
10	52947471	52954397	6926	0.06
10	58080923	58128237	47314	0.11
10	65125333	65145717	20384	0.80
10	68672593	68815771	143178	0.46
10	68888627	68937485	48858	0.11
10	71862744	71880676	17932	0.40
10	80028446	80035330	6884	0.06
10	81034435	81040988	6553	0.06
10	82650138	82671673	21535	0.11
10	89864235	89884669	20434	0.06
10	100578852	100639197	60345	0.11
10	101249045	101590420	341375	0.06
10	109230352	109282708	52356	0.11
10	112140240	112207986	67746	0.06
10	124357910	124360857	2947	0.29
11	1120324	1649253	528929	5.16
11	2044100	2082296	38196	1.26
11	30529700	30534390	4690	0.06
11	42947325	42951703	4378	0.11
11	50220667	50821348	600681	5.73
11	62213425	62236100	22675	0.11
11	62324795	62381373	56578	0.34
11	62414123	62420194	6071	0.06
11	65749835	65799248	49413	3.96
11	65816789	65969132	152343	3.73
11	66040897	66061986	21089	0.23
11	66149680	66179692	30012	0.06
11	83142361	83165228	22867	0.06

11	86859657	86862268	2611	0.11
11	94731919	94750995	19076	0.06
11	104480071	104506228	26157	0.06
11	107853742	107991310	137568	0.11
11	113106744	113108781	2037	0.06
11	115686504	115698339	11835	0.06
11	116789734	116792069	2335	0.06
11	124250072	124265029	14957	0.06
11	127972782	128094435	121653	0.06
11	133011440	133062945	51505	0.06
11	134282136	134344486	62350	0.11
12	2655540	2663115	7575	0.06
12	5122424	5284947	162523	0.06
12	6197760	6200432	2672	0.06
12	7662594	7665848	3254	0.06
12	10201980	10229648	27668	0.34
12	11304779	11325747	20968	1.20
12	17809145	17813685	4540	0.06
12	19057828	19152454	94626	0.06
12	29053138	29073147	20009	0.17
12	40244221	40252906	8685	0.06
12	58095530	58107273	11743	0.06
12	82326322	82419747	93425	0.11
12	83007562	83022106	14544	0.17
12	107314677	107326421	11744	0.06
12	114635794	114644389	8595	0.06
12	120433074	120449267	16193	0.46
12	128083027	128087592	4565	0.06
12	129043684	129055401	11717	0.06
12	130163701	130184555	20854	0.06
13	19517919	19544083	26164	0.06
13	21104754	21113428	8674	0.11
13	21372282	21379825	7543	0.06
13	23051869	23064163	12294	0.06
13	27932143	27954398	22255	0.11
13	32916728	32925590	8862	0.06
13	35752646	35760164	7518	0.06
13	36263862	36283502	19640	0.06
13	36963500	36982862	19362	1.49
13	41027566	41039590	12024	0.06
13	42308484	42322287	13803	0.06
13	42859299	42939595	80296	0.06
13	48519097	48536525	17428	0.06
13	49396404	49399506	3102	0.06
13	49436063	49470845	34782	0.06
13	53137719	53162459	24740	0.06

13	53964213	53981152	16939	0.06
13	62096640	62112385	15745	0.06
13	67068828	67231995	163167	0.06
13	79796487	79837790	41303	0.06
13	81369815	81385065	15250	0.06
13	85579573	85589662	10089	0.06
13	95972556	95991634	19078	0.06
13	96678843	96771510	92667	0.06
13	100039069	100057445	18376	0.06
13	102592905	102599717	6812	0.11
13	103032716	103049205	16489	0.11
13	107857699	107866791	9092	0.06
14	20013277	20436339	423062	0.57
14	20889996	20941547	51551	0.06
14	31517550	31521641	4091	0.06
14	34726588	34934766	208178	0.29
14	35741638	35823699	82061	0.23
14	44352777	44454516	101739	0.06
14	73304219	73318155	13936	0.06
14	77386513	77395013	8500	0.06
14	79803259	79999983	196724	0.06
14	82113991	82119980	5989	0.06
14	83004287	83036287	32000	0.06
14	91077949	91088564	10615	0.06
14	93851517	93867057	15540	0.17
14	95649237	95665238	16001	0.11
14	96987073	97344557	357484	0.06
14	97701638	97709130	7492	0.06
14	97732764	97743393	10629	0.06
14	99794334	99799894	5560	0.06
14	100132248	100256325	124077	2.70
14	100296166	100311456	15290	0.06
14	103693804	103729273	35469	0.75
15	31198520	31206286	7766	0.06
15	39918683	39920539	1856	0.97
15	51977299	52050905	73606	0.06
15	52713950	52733495	19545	0.06
15	57352069	57406764	54695	0.11
15	74606750	74636378	29628	0.57
15	75197756	75215477	17721	0.11
15	75326073	75417289	91216	0.11
15	76478144	76489014	10870	0.06
15	87127932	87149878	21946	0.23
15	100752379	100928864	176485	0.11
16	4091727	4097924	6197	0.06
16	8381113	8403035	21922	0.06

16	49216487	49260741	44254	0.06
16	53491908	53523967	32059	0.52
16	57515403	57544765	29362	0.11
16	60100657	60104607	3950	0.06
16	63726650	63730250	3600	0.06
16	63765026	63785891	20865	0.06
16	72275691	72537611	261920	0.11
16	74847725	75002535	154810	0.06
16	79226868	79239785	12917	0.17
16	81800708	81808260	7552	0.69
16	83565248	83569140	3892	0.06
16	87375682	87411805	36123	1.38
16	89542941	89572729	29788	0.57
17	4884425	4908320	23895	0.86
17	8067861	8081468	13607	0.06
17	9528319	9534195	5876	0.06
17	14296292	14316169	19877	1.20
17	19752889	19757974	5085	0.06
17	39194210	39204540	10330	0.06
17	44404704	44494310	89606	0.06
17	45304010	45321252	17242	0.11
17	49675117	49708439	33322	0.06
17	58456803	58489749	32946	0.06
17	58505032	58542806	37774	0.34
17	61382833	61416963	34130	0.34
17	69153831	69170807	16976	0.06
17	70502506	70537986	35480	0.06
17	70551531	70570984	19453	0.06
17	77219021	77237877	18856	0.57
17	77552529	77658640	106111	0.06
17	78239710	78252585	12875	0.46
17	78577923	78601339	23416	0.17
17	79369266	79395063	25797	0.57
17	80630475	80642647	12172	0.23
18	820381	828428	8047	0.40
18	11244991	11588946	343955	0.17
18	12195107	12328985	133878	0.06
18	28280627	28302121	21494	0.06
18	31849736	31925797	76061	0.75
18	31939201	31947921	8720	0.06
18	41062075	41076217	14142	0.06
18	41277457	41283486	6029	0.06
18	49486464	49497269	10805	0.06
18	52303301	52314008	10707	0.06
18	57509248	57671992	162744	0.11
18	58579210	58778838	199628	0.11

18	74440613	74479641	39028	0.06
18	78628305	78639959	11654	0.06
19	3328320	3335576	7256	0.06
19	3383395	3410259	26864	0.06
19	3479373	3703130	223757	4.59
19	3738651	3805890	67239	2.58
19	4780935	4818565	37630	0.29
19	6895855	7106549	210694	0.57
19	8493358	8529661	36303	1.55
19	11397501	11414796	17295	0.11
19	14391379	14427808	36429	0.80
19	14464147	14483799	19652	0.06
19	14495415	14542296	46881	0.86
19	14553016	14575034	22018	0.11
19	15667349	15727059	59710	4.19
19	17497707	17541587	43880	0.11
19	19375426	19409001	33575	0.11
19	19730943	19773382	42439	0.06
19	31343072	31355686	12614	0.06
19	32383819	32388358	4539	1.26
19	33275568	33281400	5832	0.06
19	33719877	33744316	24439	0.06
19	35601587	35657649	56062	0.92
19	35872568	35905132	32564	0.11
19	35996244	36010543	14299	0.11
19	36096969	36129876	32907	1.26
19	36504018	36537641	33623	0.17
19	36840712	36858366	17654	0.06
19	36898545	36922545	24000	0.06
19	37065771	37095148	29377	0.06
19	38206351	38226738	20387	0.17
19	38304947	38491143	186196	0.92
19	38827465	38913817	86352	0.57
19	40402976	40405580	2604	0.06
19	41155272	41194588	39316	0.06
19	48395295	48514469	119174	1.89
19	48708450	48729709	21259	0.11
19	48748517	48761108	12591	0.06
19	50475637	50514469	38832	0.11
19	50981734	50988343	6609	0.11
19	51081694	51130501	48807	0.63
19	52490367	52540022	49655	0.11
19	56660842	56671001	10159	0.06
20	571277	578790	7513	0.06
20	2560993	2563158	2165	0.06
20	3009168	3016469	7301	0.06

20	5946028	5958807	12779	0.06
20	7026059	7199049	172990	0.06
20	9733230	9767480	34250	0.06
20	23859357	23871343	11986	0.06
20	24436524	24443584	7060	0.06
20	24707683	24781454	73771	0.06
20	33412545	33423541	10996	0.06
20	40715632	40753880	38248	0.06
20	44965137	44996412	31275	0.06
20	60376492	60387400	10908	0.06
20	60992315	61014216	21901	1.49
20	63087338	63106936	19598	0.75
21	14245760	14288523	42763	0.06
21	41854544	41860297	5753	0.06
21	43832191	43863789	31598	0.29
21	45228126	45366638	138512	0.52
22	23014571	23022202	7631	0.17
22	23381534	23400522	18988	0.17
22	32410344	32449740	39396	0.06
22	33325334	33331783	6449	0.06
22	34464592	34495618	31026	0.06
22	42734392	42781650	47258	0.06
22	43383514	43388500	4986	0.06
22	44783504	44825700	42196	0.06
22	45098951	45104637	5686	0.06
22	45170586	45193936	23350	0.11
22	49077502	49081089	3587	0.11
Deletions				
1	1110158	1171304	61146	0.06
1	1275031	1606824	331793	0.92
1	1622382	1736859	114477	1.09
1	1916447	1947645	31198	0.17
1	2577613	2743008	165395	0.34
1	3060252	3066299	6047	0.06
1	3093405	3106763	13358	0.34
1	3413826	3427238	13412	0.06
1	4058927	4063057	4130	0.11
1	4969471	4995544	26073	0.06
1	5053851	5114018	60167	0.06
1	5294499	5298656	4157	0.11
1	5595707	5603031	7324	0.06
1	5973659	5981226	7567	0.06
1	6423523	6444559	21036	0.40
1	6464589	6475089	10500	0.17
1	6629764	6636785	7021	0.17
1	6939678	6942334	2656	0.06

1	7117509	7128361	10852	0.11
1	7294057	7313767	19710	0.11
1	7444081	7458046	13965	0.11
1	7497092	7503841	6749	0.06
1	9346920	9352349	5429	0.11
1	9596879	9626134	29255	0.63
1	9782518	9795471	12953	0.63
1	11038275	11054028	15753	0.23
1	11661559	11674027	12468	0.06
1	12191261	12207407	16146	0.23
1	14864756	14866420	1664	0.06
1	15570525	15574806	4281	0.06
1	15595279	15605747	10468	0.23
1	15725847	15752981	27134	0.52
1	16186658	16191431	4773	0.06
1	16231647	16232908	1261	0.06
1	16977530	17013444	35914	0.29
1	17492083	17494864	2781	0.06
1	18250219	18275606	25387	0.17
1	18801641	18813343	11702	0.06
1	19342389	19344116	1727	0.06
1	19397585	19405379	7794	0.06
1	20295419	20299054	3635	0.06
1	21139102	21170218	31116	0.06
1	21535163	21541666	6503	0.11
1	21587361	21599655	12294	0.29
1	21632043	21633097	1054	0.06
1	22027617	22031562	3945	0.06
1	22173037	22183413	10376	1.32
1	23643640	23647541	3901	0.06
1	24058667	24060378	1711	0.06
1	28008170	28067150	58980	0.06
1	30007244	30015473	8229	0.17
1	30559046	30567587	8541	0.11
1	30803715	30806609	2894	0.11
1	31207466	31215111	7645	0.11
1	31689862	31696584	6722	0.06
1	33225076	33232248	7172	0.06
1	34237230	34249092	11862	0.06
1	34325265	34331747	6482	0.11
1	34451840	34456759	4919	0.11
1	34780696	34786068	5372	0.17
1	35163104	35173903	10799	0.06
1	38326229	38331771	5542	0.06
1	38456381	38461513	5132	0.06
1	39523340	39528131	4791	0.06

1	39590285	39619228	28943	0.06
1	40270145	40405825	135680	0.34
1	42733444	42747828	14384	0.11
1	43994967	43999686	4719	0.17
1	44730872	44733738	2866	0.06
1	45778902	45782973	4071	0.06
1	47564055	47565491	1436	0.06
1	49243451	49304339	60888	0.06
1	51010270	51151493	141223	0.29
1	51170977	51198700	27723	0.06
1	51312913	51349807	36894	0.11
1	51898040	51918293	20253	0.17
1	52785243	52867444	82201	0.97
1	53779830	53806157	26327	0.06
1	54237914	54241938	4024	0.11
1	54291478	54293257	1779	0.06
1	54811757	54818385	6628	0.11
1	55101737	55106280	4543	0.06
1	55943205	55947904	4699	0.06
1	57447555	57449213	1658	0.06
1	58727216	58737449	10233	0.69
1	59578285	59581012	2727	0.11
1	61559613	61562731	3118	0.06
1	61939375	61990763	51388	1.15
1	62147311	62150211	2900	0.06
1	62300855	62303472	2617	0.06
1	66437680	66451277	13597	0.11
1	66879399	66902195	22796	0.34
1	70698032	70701995	3963	0.17
1	70779404	70780985	1581	0.06
1	70837818	70847638	9820	0.06
1	70949180	70999715	50535	0.11
1	72138644	72142975	4331	0.06
1	73998371	74000628	2257	0.06
1	75876281	75924101	47820	0.17
1	76398127	76401224	3097	0.11
1	77119801	77128889	9088	0.06
1	77307994	77315882	7888	0.69
1	78170655	78196099	25444	0.17
1	79617744	79693536	75792	0.11
1	79739774	79755294	15520	0.11
1	80866323	80877187	10864	0.06
1	85191787	85200435	8648	0.06
1	85540360	85543257	2897	0.06
1	86325278	86329156	3878	0.06
1	86562986	86573626	10640	0.11

1	89406347	89420670	14323	0.06
1	90082050	90091422	9372	0.06
1	90441425	90454465	13040	0.40
1	90785174	90799531	14357	0.06
1	93123210	93138397	15187	0.06
1	93516968	93543504	26536	0.11
1	93989892	94005667	15775	0.06
1	100185108	100187311	2203	0.17
1	101543796	101556807	13011	0.11
1	101622813	101635734	12921	0.06
1	102022230	102027196	4966	0.11
1	102150487	102176695	26208	0.06
1	103612312	103668424	56112	1.32
1	103778424	103814778	36354	0.40
1	104417085	104435781	18696	0.06
1	104961901	104975030	13129	0.06
1	105764986	105774609	9623	1.20
1	107113734	107118984	5250	0.17
1	108102082	108115312	13230	0.06
1	110522948	110528657	5709	0.11
1	112758014	112759035	1021	0.06
1	112964556	112985309	20753	0.06
1	114287816	114294399	6583	0.06
1	114559503	114610193	50690	0.06
1	114850938	114954438	103500	0.06
1	115351066	115357742	6676	0.06
1	117572513	117581951	9438	0.11
1	119629919	119639892	9973	0.06
1	143272542	143284605	12063	0.06
1	147001352	147243554	242202	0.06
1	153321880	153324551	2671	0.06
1	153821908	153866569	44661	0.29
1	155051855	155063753	11898	0.06
1	155179729	155186056	6327	0.11
1	156366136	156367343	1207	0.06
1	156832865	156867624	34759	0.06
1	158523936	158527961	4025	0.11
1	158928732	158932486	3754	0.06
1	160834954	160837925	2971	0.06
1	161519677	161649951	130274	4.36
1	164143425	164148771	5346	0.06
1	164778171	164781379	3208	0.06
1	164967529	164975726	8197	0.06
1	165931994	165940619	8625	0.34
1	166496641	166499192	2551	0.17
1	166729898	166736387	6489	0.06

1	166751662	166764782	13120	0.17
1	167053025	167062646	9621	0.17
1	168002785	168020293	17508	0.17
1	168045971	168063858	17887	0.06
1	169548890	169558232	9342	0.06
1	169934721	169974038	39317	0.11
1	170398412	170417993	19581	0.63
1	171812595	171819642	7047	0.06
1	172132939	172147658	14719	0.06
1	173106734	173118193	11459	0.11
1	174871961	174887622	15661	0.06
1	175881588	175884181	2593	0.06
1	179378546	179414971	36425	0.11
1	179986526	180044908	58382	0.34
1	180936158	180939925	3767	0.06
1	182115351	182118149	2798	0.11
1	184992503	185001245	8742	0.17
1	185065971	185073437	7466	0.06
1	185447125	185448984	1859	0.11
1	186689993	186707910	17917	0.06
1	186781174	186788209	7035	0.11
1	186815008	186819132	4124	0.11
1	187579227	187661054	81827	0.11
1	187672082	187813897	141815	0.17
1	189203553	189216195	12642	0.06
1	189414281	189466224	51943	0.40
1	190444895	190488210	43315	0.06
1	192231136	192245934	14798	0.17
1	193654681	193665521	10840	0.06
1	194330252	194339923	9671	0.46
1	195144854	195157599	12745	0.06
1	195853127	195857608	4481	0.06
1	195861608	195906930	45322	0.11
1	196753917	196949015	195098	2.75
1	196994083	197005726	11643	0.11
1	199524893	199527234	2341	0.06
1	200244430	200252163	7733	0.06
1	202008126	202017290	9164	0.06
1	203935438	203952747	17309	0.40
1	204001686	204006494	4808	0.17
1	205291838	205294176	2338	0.06
1	206009205	206024848	15643	0.29
1	206867593	206870398	2805	0.29
1	210162694	210170191	7497	0.06
1	210429364	210441720	12356	1.38
1	211051576	211056094	4518	0.06

1	215204065	215217236	13171	0.06
1	215632217	215638288	6071	0.06
1	215684433	215688450	4017	0.11
1	219435234	219442274	7040	0.06
1	219718860	219723318	4458	0.23
1	219846955	219863296	16341	0.17
1	221550077	221646478	96401	0.06
1	224240559	224245465	4906	0.11
1	227258918	227295105	36187	0.23
1	227541523	227546189	4666	0.06
1	227729781	227734013	4232	0.06
1	227920883	227927608	6725	0.11
1	228265196	228287116	21920	0.34
1	228333272	228341428	8156	0.06
1	229042665	229048940	6275	0.06
1	229425427	229435823	10396	0.11
1	233173672	233270885	97213	0.06
1	235885621	235893517	7896	0.06
1	237281283	237292995	11712	0.06
1	239332589	239348499	15910	0.06
1	239364037	239368203	4166	0.11
1	240557416	240570126	12710	0.17
1	240675636	240679137	3501	0.06
1	242148962	242168650	19688	0.23
1	242863455	242889755	26300	0.06
1	243526130	243532300	6170	0.06
1	243801600	243835432	33832	0.06
1	243870703	243876706	6003	0.06
1	243895793	243898198	2405	0.11
1	245115505	245121590	6085	0.11
1	245171804	245174422	2618	0.06
1	245523501	245531430	7929	0.06
1	245704055	245705997	1942	0.06
1	245869685	245886945	17260	0.06
1	245933015	245937851	4836	0.06
1	246511474	246524284	12810	0.11
1	247113931	247120194	6263	0.06
1	247961134	247968086	6952	0.06
1	247988697	248052014	63317	0.06
1	248134229	248423931	289702	0.11
2	1490584	1521942	31358	0.40
2	1768191	1772021	3830	0.06
2	2245781	2254085	8304	0.06
2	4263430	4277582	14152	2.64
2	4447338	4455357	8019	0.06
2	6698995	6706983	7988	0.06

2	8030032	8038092	8060	0.06
2	11667873	11668906	1033	0.06
2	13028107	13038338	10231	0.06
2	13063749	13069755	6006	0.06
2	13222805	13483232	260427	0.06
2	16245008	16253725	8717	0.06
2	16927367	17069465	142098	0.40
2	17990546	18012011	21465	0.11
2	19187033	19214748	27715	0.06
2	19376830	19382654	5824	0.06
2	19901462	19909300	7838	0.11
2	20209561	20213549	3988	0.29
2	21778103	21972801	194698	0.06
2	24870341	24883357	13016	0.06
2	27541603	27560221	18618	0.69
2	28169927	28175897	5970	0.06
2	28995030	29003693	8663	0.06
2	29312001	29322054	10053	0.06
2	29508922	29510935	2013	0.11
2	30395925	30403433	7508	0.23
2	30959296	30965022	5726	0.06
2	32069551	32218985	149434	0.69
2	32529919	32551696	21777	0.06
2	32575295	32613697	38402	0.06
2	33373150	33377504	4354	0.11
2	33647327	33659035	11708	0.06
2	34004838	34009275	4437	0.06
2	34592704	34684569	91865	0.17
2	34836144	34843217	7073	0.63
2	34871069	34876455	5386	0.06
2	35073345	35094222	20877	0.06
2	35162475	35174896	12421	0.11
2	35295108	35411515	116407	1.03
2	35569994	35576281	6287	0.06
2	35784777	35825818	41041	0.06
2	35954279	35961079	6800	0.17
2	36186946	36192396	5450	0.06
2	36199138	36216526	17388	0.06
2	36984820	36998563	13743	0.06
2	37847596	37850640	3044	0.06
2	38054621	38088540	33919	0.06
2	38158760	38166095	7335	0.06
2	39555186	39625632	70446	0.17
2	39975365	39982372	7007	0.06
2	40192989	40202969	9980	0.11
2	40471781	40483813	12032	0.06

2	41128212	41146848	18636	0.11
2	41555200	41685107	129907	0.11
2	41715849	41778515	62666	0.06
2	42762452	42771804	9352	0.06
2	42889285	42893290	4005	0.06
2	42919660	42927070	7410	0.11
2	43300350	43307376	7026	0.06
2	43349204	43351911	2707	0.06
2	45104848	45136523	31675	0.06
2	45650229	45652166	1937	0.11
2	45817739	45820667	2928	0.06
2	47019760	47032358	12598	0.69
2	47287895	47296270	8375	0.06
2	47339963	47348474	8511	0.06
2	47817633	47859715	42082	0.40
2	48814183	48827884	13701	0.06
2	49415695	49480794	65099	0.06
2	50271467	50286636	15169	0.23
2	50679587	50852344	172757	0.11
2	51700780	51757206	56426	0.06
2	52774904	52831803	56899	0.17
2	52997838	52998995	1157	0.06
2	53118152	53134593	16441	0.06
2	53727113	53741637	14524	0.06
2	53797489	53837843	40354	0.06
2	54138692	54293217	154525	0.11
2	55285451	55371790	86339	0.52
2	55734424	55736283	1859	0.06
2	55768874	55771314	2440	0.06
2	55783471	55788114	4643	0.34
2	55844427	55849699	5272	0.17
2	57013142	57016078	2936	0.06
2	57175857	57248779	72922	1.55
2	57322316	57372378	50062	0.52
2	57593494	57625171	31677	0.17
2	58082239	58091263	9024	0.06
2	59721994	59725802	3808	0.17
2	59865651	59874077	8426	0.40
2	60717889	60730858	12969	0.06
2	60823058	60829881	6823	0.06
2	61079619	61096705	17086	0.06
2	61734294	61735846	1552	0.06
2	62508790	62531798	23008	0.06
2	64287849	64295173	7324	0.06
2	65735926	65755012	19086	0.06
2	67532940	67539817	6877	1.43

2	70132213	70157862	25649	0.06
2	71486608	71488535	1927	0.06
2	75139501	75147139	7638	0.06
2	76365730	76394126	28396	0.06
2	76416141	76438908	22767	0.06
2	76710320	76721975	11655	0.11
2	77044128	77051347	7219	0.06
2	78053222	78071863	18641	0.11
2	78476432	78496536	20104	0.46
2	78599108	78648180	49072	0.11
2	78673143	78677228	4085	0.06
2	78913837	78923956	10119	0.06
2	79104957	79112779	7822	0.06
2	79135028	79148459	13431	0.34
2	79298917	79303938	5021	0.06
2	79833869	79839372	5503	0.06
2	80052740	80060550	7810	0.06
2	83020535	83025643	5108	0.06
2	83307746	83364729	56983	0.40
2	84963202	84965158	1956	0.29
2	85698557	85710877	12320	0.11
2	98241725	98269462	27737	0.57
2	100548429	100556794	8365	0.06
2	101649552	101671866	22314	0.06
2	105724357	105736650	12293	0.06
2	109497570	109529214	31644	0.23
2	110079565	110224953	145388	0.23
2	111028554	111032214	3660	0.06
2	111173844	111186961	13117	0.06
2	111932598	112048667	116069	0.11
2	112295484	112340863	45379	1.15
2	114324147	114388747	64600	0.11
2	115009881	115031811	21930	0.06
2	115938858	115972645	33787	0.11
2	118675643	118678127	2484	0.29
2	120919779	120924640	4861	0.06
2	121043177	121050200	7023	0.06
2	121110331	121114826	4495	0.06
2	121317246	121323934	6688	0.06
2	122019635	122041141	21506	0.06
2	122290358	122296333	5975	0.06
2	122842842	122851976	9134	0.06
2	123545171	123549188	4017	0.06
2	124055641	124058530	2889	0.06
2	124496293	124499348	3055	0.06
2	125372681	125378695	6014	0.06

2	125861332	125910808	49476	1.20
2	126703219	126708072	4853	0.06
2	127084807	127086143	1336	0.06
2	127203694	127205692	1998	0.06
2	127346138	127352644	6506	0.06
2	127794853	127797562	2709	1.26
2	128261966	128293335	31369	0.23
2	128359348	128389632	30284	0.34
2	129240870	129253957	13087	0.06
2	129439035	129441794	2759	0.17
2	130915650	130931210	15560	0.06
2	131554102	131572603	18501	0.17
2	132852784	132977500	124716	0.06
2	133877623	133900277	22654	0.06
2	134986023	134988995	2972	0.11
2	135194835	135246359	51524	0.23
2	135277266	135327091	49825	0.23
2	135491143	135507061	15918	0.06
2	135794618	135827309	32691	0.06
2	135903649	135934255	30606	0.23
2	136038449	136042684	4235	0.06
2	137014197	137026022	11825	0.11
2	137199083	137206047	6964	0.06
2	139038548	139049328	10780	0.11
2	139438950	139450933	11983	0.17
2	139802256	139806048	3792	0.06
2	141397190	141406720	9530	0.06
2	145337502	145362943	25441	0.06
2	147272776	147304669	31893	0.06
2	147737716	147743405	5689	0.06
2	148251936	148259243	7307	0.11
2	148408754	148412896	4142	0.06
2	149510891	149544289	33398	0.06
2	151275616	151282757	7141	0.06
2	151379825	151450921	71096	0.06
2	153833707	153835985	2278	0.34
2	154728379	154735898	7519	0.06
2	155499688	155528834	29146	0.11
2	155551971	155563528	11557	0.06
2	158116904	158121813	4909	0.52
2	158307390	158414794	107404	0.06
2	159046537	159053899	7362	0.06
2	160121183	160129411	8228	0.06
2	163582547	163588672	6125	1.43
2	164759961	164790831	30870	0.11
2	166731755	166734263	2508	0.11

2	167088838	167092628	3790	0.23
2	168277286	168287738	10452	0.23
2	168966776	168980220	13444	0.34
2	169598697	169607041	8344	0.23
2	170473853	170478348	4495	0.06
2	170549561	170552759	3198	0.06
2	172238412	172245247	6835	0.06
2	173429489	173433007	3518	0.06
2	173828460	173834208	5748	0.06
2	174280106	174286198	6092	0.06
2	175767187	175771473	4286	0.06
2	176509328	176512639	3311	0.69
2	178632298	178670552	38254	0.06
2	179635867	179637629	1762	0.06
2	179810942	179835970	25028	0.06
2	182722555	182757820	35265	0.17
2	183580266	183643772	63506	0.06
2	183798045	184036734	238689	2.87
2	184123053	184126290	3237	0.06
2	184174332	184270717	96385	1.78
2	185331576	185341365	9789	0.06
2	187075283	187105206	29923	0.06
2	187330200	187343989	13789	0.06
2	187775849	187777595	1746	0.17
2	189783193	189784669	1476	0.06
2	189957482	189966952	9470	0.06
2	192009533	192012520	2987	0.06
2	192411285	192420369	9084	0.69
2	194795354	194813153	17799	0.06
2	195774599	195782815	8216	0.11
2	197747609	197785181	37572	0.63
2	200751667	200805220	53553	0.06
2	200920934	200934696	13762	0.23
2	202875062	202980578	105516	0.46
2	204740447	204745132	4685	0.06
2	206996787	207000187	3400	0.06
2	208069842	208086648	16806	0.92
2	208371237	208381725	10488	0.40
2	208776550	208784068	7518	0.06
2	208822617	208849604	26987	0.06
2	209032828	209037065	4237	0.06
2	209604483	209627500	23017	0.11
2	209858018	210002084	144066	0.06
2	210905997	210913548	7551	0.29
2	211790092	211797374	7282	0.06
2	211938040	211943584	5544	0.11

2	212174751	212176608	1857	0.06
2	212182615	212209010	26395	0.11
2	213710404	213716400	5996	0.11
2	213734481	213752856	18375	0.06
2	214137345	214149800	12455	0.06
2	217008807	217010795	1988	0.11
2	217392920	217396646	3726	1.32
2	219365406	219376828	11422	0.06
2	221200528	221208781	8253	0.06
2	221382209	221389570	7361	0.17
2	221991853	222000831	8978	0.06
2	222142370	222145464	3094	0.06
2	222534062	222539963	5901	0.06
2	222761309	222773184	11875	0.29
2	225590902	225595624	4722	0.06
2	225917548	225919064	1516	0.06
2	226480649	226482737	2088	0.75
2	226901684	226943609	41925	0.06
2	227595968	227601271	5303	0.06
2	227658238	227659691	1453	0.11
2	227740475	227748553	8078	0.17
2	230330332	230359923	29591	0.06
2	232761878	232767430	5552	0.11
2	233222032	233228150	6118	0.11
2	233469518	233471537	2019	0.34
2	233793040	233809023	15983	0.23
2	234024915	234047011	22096	0.29
2	234655499	234663275	7776	0.06
2	236073414	236076748	3334	0.06
2	238533360	238537442	4082	0.06
2	238910646	238912828	2182	0.06
2	240265192	240271928	6736	0.29
2	240888617	240933763	45146	0.57
2	241003836	241007705	3869	0.06
2	241186277	241203684	17407	0.11
3	783581	790906	7325	0.06
3	856978	861485	4507	0.06
3	956829	964745	7916	0.34
3	1417834	1422665	4831	0.06
3	1626248	1641470	15222	0.11
3	1662989	1670726	7737	0.17
3	3547248	3551915	4667	0.06
3	3725502	3735448	9946	0.06
3	4542969	4551438	8469	0.06
3	5368137	5378490	10353	0.06
3	5385605	5411021	25416	0.11

3	6036962	6059253	22291	0.34
3	6586069	6594282	8213	0.06
3	6607961	6677308	69347	0.06
3	6909768	6913684	3916	0.06
3	7930973	7949976	19003	0.06
3	9065174	9069025	3851	0.06
3	9131261	9133167	1906	0.29
3	9321411	9325935	4524	0.11
3	9593787	9631334	37547	0.34
3	10131808	10134889	3081	0.11
3	10198225	10200559	2334	0.06
3	10724944	10735947	11003	0.17
3	11185647	11191093	5446	0.06
3	11910141	11913634	3493	0.06
3	12360975	12369203	8228	0.06
3	12608077	12621734	13657	0.06
3	13702560	13714384	11824	0.11
3	14249629	14252672	3043	0.11
3	15296693	15302747	6054	0.11
3	16586082	16614824	28742	0.29
3	20949247	20954543	5296	0.29
3	22039274	22050563	11289	0.06
3	22257173	22261581	4408	0.06
3	22390535	22462409	71874	0.06
3	23594145	23603962	9817	0.06
3	24728009	24758050	30041	0.23
3	24850914	24866377	15463	0.06
3	25034833	25040366	5533	0.06
3	25476981	25480212	3231	0.06
3	25907809	25923756	15947	0.17
3	26188876	26267563	78687	0.06
3	27771171	27776465	5294	0.11
3	27795987	27800796	4809	0.06
3	28769097	28777632	8535	0.52
3	29177405	29183409	6004	0.06
3	29909894	29914416	4522	0.11
3	30414431	30428535	14104	0.06
3	30868982	30872545	3563	0.75
3	32456596	32458456	1860	0.06
3	33275728	33306037	30309	0.06
3	33779310	33784319	5009	0.06
3	34687261	34757912	70651	0.23
3	35358899	35372656	13757	0.06
3	36002719	36005882	3163	0.06
3	36024552	36025686	1134	0.06
3	36358551	36375195	16644	0.11

3	36387326	36417762	30436	0.11
3	38096133	38126634	30501	0.29
3	38575635	38588323	12688	0.06
3	39183299	39189506	6207	0.46
3	39313948	39356535	42587	0.06
3	39540301	39548800	8499	0.17
3	39693421	39755235	61814	0.17
3	40396235	40400417	4182	0.06
3	41051056	41101296	50240	0.06
3	45096854	45098015	1161	0.11
3	45592947	45597960	5013	0.06
3	46368545	46378613	10068	0.11
3	46406413	46408806	2393	0.06
3	48517813	48527685	9872	0.06
3	48795824	48853842	58018	0.23
3	49052843	49078387	25544	0.06
3	49199961	49238644	38683	0.06
3	49643540	49671149	27609	0.23
3	50691566	50775465	83899	0.23
3	50936609	50947823	11214	0.06
3	52034720	52054635	19915	0.11
3	53949727	53954905	5178	0.06
3	54225686	54233814	8128	0.06
3	57204489	57210880	6391	0.06
3	57594600	57664278	69678	0.06
3	60119602	60470175	350573	0.23
3	60816850	60823544	6694	0.11
3	62679547	62708646	29099	0.34
3	63142628	63153064	10436	0.80
3	63710616	63733152	22536	0.06
3	65243680	65248876	5196	0.06
3	65456392	65466500	10108	0.06
3	66941950	66947130	5180	0.06
3	66980228	66987517	7289	0.06
3	67609725	67615621	5896	0.06
3	68803595	68809588	5993	0.06
3	69014785	69044447	29662	0.11
3	72837424	72843210	5786	0.06
3	74480075	74485938	5863	0.06
3	75666750	75685682	18932	0.17
3	76206829	77025877	819048	0.23
3	77898557	77920814	22257	0.06
3	78865951	78901778	35827	0.06
3	79285456	79291571	6115	0.06
3	80329517	80336640	7123	0.06
3	83625305	83633236	7931	0.06

3	84460374	84520244	59870	0.23
3	84779958	84808776	28818	0.06
3	94805942	94814769	8827	0.06
3	96347328	96350171	2843	0.06
3	96500117	96511216	11099	0.06
3	98134315	98167403	33088	0.06
3	98180562	98190822	10260	0.06
3	98393807	98403400	9593	0.23
3	103227372	103231851	4479	0.17
3	103309291	103313495	4204	0.06
3	103801514	104210570	409056	0.69
3	106500281	106515006	14725	1.61
3	109675987	109703091	27104	0.06
3	110205386	110221729	16343	0.06
3	110538126	110571996	33870	0.40
3	111893344	111900324	6980	0.06
3	113073418	113086339	12921	0.06
3	118054625	118058212	3587	0.17
3	118229805	118239552	9747	0.06
3	121622536	121637521	14985	0.06
3	124722390	124730444	8054	0.06
3	124796467	124803356	6889	0.06
3	125532112	125593286	61174	0.06
3	126146923	126156736	9813	0.11
3	126212760	126237733	24973	0.11
3	126508342	126520083	11741	0.06
3	127697630	127702643	5013	0.06
3	128944316	128946170	1854	0.06
3	130240625	130253047	12422	0.06
3	131969668	131988000	18332	0.06
3	132555669	132558523	2854	0.06
3	132577576	133273326	695750	0.23
3	133297470	133304724	7254	0.29
3	135124398	135125537	1139	0.11
3	136231248	136237797	6549	0.11
3	136354321	136512833	158512	0.29
3	137420294	137430107	9813	0.11
3	138708211	138780640	72429	0.23
3	138841359	138906711	65352	0.06
3	141622158	141626891	4733	0.11
3	141976426	142027242	50816	0.63
3	142311077	142391976	80899	0.11
3	143446660	143448480	1820	0.06
3	143526166	143536439	10273	0.06
3	144433380	144463191	29811	0.06
3	144563957	144576198	12241	0.80

3	145755495	145767228	11733	0.06
3	145911296	145944666	33370	0.17
3	146572295	146586682	14387	0.06
3	148807150	148815610	8460	0.06
3	151021288	151025736	4448	0.40
3	152053357	152058190	4833	0.11
3	152696295	152699435	3140	0.17
3	153101712	153141427	39715	0.06
3	153748607	153752474	3867	0.63
3	154573689	154728118	154429	0.23
3	155986074	155990743	4669	0.06
3	156162266	156171187	8921	0.06
3	159075161	159095191	20030	1.72
3	160962985	160968000	5015	0.63
3	163525018	163540186	15168	0.06
3	164584512	164593341	8829	0.46
3	164806471	164831106	24635	0.23
3	165491049	165501356	10307	0.06
3	165803043	165831591	28548	0.06
3	165865082	165868486	3404	0.06
3	166071491	166087515	16024	0.17
3	172505419	172508450	3031	0.06
3	172869947	172874521	4574	0.06
3	173461508	173463651	2143	0.06
3	174385597	174391348	5751	0.06
3	175257462	175287164	29702	0.11
3	175596580	175601112	4532	0.06
3	176169594	176189554	19960	0.92
3	177230472	177237552	7080	0.06
3	178387427	178403079	15652	0.11
3	179154567	179185476	30909	0.06
3	181971859	181993485	21626	0.06
3	182464458	182478805	14347	0.06
3	183579399	183583986	4587	0.11
3	183637058	183691779	54721	0.17
3	183902898	183906222	3324	0.06
3	183910797	183917700	6903	0.06
3	184051895	184054726	2831	0.06
3	184088267	184093444	5177	0.11
3	187169261	187171532	2271	0.06
3	189364452	189386147	21695	0.40
3	191611935	191619528	7593	0.34
3	191682341	191694808	12467	0.06
3	191803201	191846961	43760	0.11
3	191986209	191990586	4377	0.06
3	192085531	192093330	7799	0.06

3	192376131	192380577	4446	0.06
3	193084652	193093500	8848	0.06
3	194171321	194173223	1902	0.17
3	194673000	194682701	9701	0.06
3	194693381	194719470	26089	0.06
3	194829981	194836910	6929	0.06
3	194956616	194965177	8561	0.06
3	194990318	194993425	3107	0.17
3	195041874	195047034	5160	0.11
3	195467922	195472712	4790	0.06
3	195864185	195893166	28981	0.23
3	196026869	196039866	12997	0.34
3	196423307	196453455	30148	0.17
3	197023336	197028466	5130	0.63
3	197326102	197330015	3913	0.06
3	197439620	197443665	4045	0.06
4	1221731	1231023	9292	0.06
4	1349548	1386277	36729	0.57
4	1531673	1571414	39741	0.11
4	3273559	3274764	1205	0.17
4	3483685	3532851	49166	2.18
4	3858787	3867523	8736	0.11
4	3909724	4172117	262393	0.57
4	4358615	4363339	4724	0.06
4	5839850	5915901	76051	0.17
4	5990359	5994691	4332	0.23
4	7135305	7138704	3399	0.11
4	7144623	7147886	3263	0.17
4	7181189	7185670	4481	0.40
4	7446197	7459376	13179	0.17
4	7922236	7931111	8875	0.06
4	8217755	8238103	20348	0.46
4	8637198	8641733	4535	0.06
4	8643020	8648129	5109	0.23
4	8650998	8657219	6221	0.11
4	8668614	8674143	5529	0.06
4	9522093	9585140	63047	0.06
4	9993558	9997174	3616	0.11
4	10194898	10202797	7899	0.06
4	11604236	11605291	1055	0.06
4	12756993	12770879	13886	0.06
4	14341584	14348528	6944	0.23
4	14507175	14521998	14823	0.06
4	14910729	14938665	27936	3.78
4	15226735	15229726	2991	0.11
4	18791889	18807804	15915	0.06

4	19990179	19994993	4814	0.11
4	21447157	21464941	17784	0.17
4	21516317	21574000	57683	0.34
4	22173435	22178154	4719	0.06
4	23448232	23451487	3255	0.06
4	25054956	25059136	4180	0.57
4	25500761	25506358	5597	0.11
4	25733420	25741710	8290	0.23
4	25967002	25982443	15441	0.06
4	26210471	26215653	5182	0.06
4	26441532	26452604	11072	0.11
4	26790596	26796037	5441	0.06
4	28141535	28149200	7665	0.06
4	28617248	28621565	4317	0.06
4	29926294	29928811	2517	0.11
4	31356170	31361660	5490	0.06
4	32374380	32386775	12395	0.06
4	32393088	32408573	15485	0.06
4	32438821	32441347	2526	0.06
4	34005795	34044853	39058	0.06
4	34124386	34133982	9596	0.06
4	34487675	34489317	1642	0.23
4	34770604	34853652	83048	0.06
4	35692875	35696259	3384	0.11
4	35774101	35776010	1909	0.06
4	35920542	35977120	56578	0.06
4	37991633	38011405	19772	0.06
4	38455108	38464757	9649	0.06
4	38719221	38726455	7234	0.17
4	39496873	39499466	2593	0.06
4	39568333	39742743	174410	1.09
4	39816902	39842473	25571	0.06
4	39947882	40046209	98327	0.40
4	40151635	40158320	6685	0.06
4	41404351	41405729	1378	0.11
4	45008827	45035653	26826	3.04
4	53729858	53736915	7057	0.17
4	56683362	56684704	1342	0.06
4	56841303	56869833	28530	0.06
4	57390500	57398396	7896	0.06
4	57947265	57952232	4967	0.23
4	58547806	58552475	4669	0.06
4	58791948	58806872	14924	0.06
4	61602248	61612986	10738	0.06
4	61863688	61874789	11101	0.06
4	62492811	62496637	3826	0.06

4	63377655	63387913	10258	0.06
4	63521603	63547049	25446	0.06
4	64458635	64477743	19108	0.40
4	64956382	64974326	17944	0.80
4	65828363	65835161	6798	0.06
4	67739220	67740402	1182	0.06
4	68527638	68623755	96117	2.98
4	69165902	69176016	10114	0.06
4	69616698	69618239	1541	0.06
4	69718320	69748645	30325	0.06
4	69899290	69908682	9392	0.06
4	70294290	70414090	119800	0.11
4	70873002	70883208	10206	0.06
4	72209829	72247656	37827	0.06
4	75408966	75496578	87612	0.29
4	74831557	74859264	27707	0.11
4	77355267	77366613	11346	0.06
4	78354306	78371084	16778	0.06
4	85179854	85202135	22281	0.34
4	88157465	88163229	5764	0.06
4	89096633	89101761	5128	0.06
4	89441959	89451415	9456	0.06
4	89660998	89679819	18821	0.29
4	90743714	90750499	6785	0.06
4	90965465	90975676	10211	0.69
4	91281608	91289031	7423	0.06
4	91513521	91524802	11281	0.11
4	91896150	91911557	15407	0.06
4	91973845	92021371	47526	0.17
4	92105522	92114771	9249	0.29
4	92625545	92669550	44005	0.23
4	92700269	92719943	19674	0.06
4	94705293	94733132	27839	0.06
4	95009325	95015217	5892	0.06
4	95440538	95480637	40099	0.06
4	95517323	95520895	3572	0.06
4	98753206	98757810	4604	0.06
4	101605156	101609521	4365	0.06
4	103068493	103075119	6626	0.06
4	103518984	103536241	17257	0.06
4	103644887	103653108	8221	0.11
4	103819338	103839927	20589	0.06
4	104345888	104352505	6617	0.11
4	104385225	104391800	6575	0.11
4	105327073	105336946	9873	0.06
4	107003419	107010899	7480	0.11

4	108453095	108454943	1848	0.06
4	108855230	108908471	53241	0.06
4	109040851	109043760	2909	0.06
4	110683327	110687329	4002	0.06
4	111285478	111291124	5646	0.06
4	111371861	111386488	14627	2.92
4	112564369	112617124	52755	0.11
4	112655003	112658006	3003	0.06
4	113433356	113442215	8859	0.17
4	114389243	114393972	4729	0.06
4	115345266	115355529	10263	0.40
4	115474508	115484442	9934	0.11
4	116393940	116404890	10950	0.06
4	116419205	116486562	67357	0.29
4	117025490	117041232	15742	0.06
4	117192874	117207581	14707	0.17
4	117223340	117245362	22022	0.06
4	117372898	117411847	38949	0.11
4	117671193	117678039	6846	0.17
4	118151707	118159626	7919	0.06
4	118356905	118390369	33464	0.17
4	120198896	120303410	104514	0.11
4	120465383	120474014	8631	0.06
4	120601503	120623983	22480	0.34
4	121394587	121413001	18414	0.11
4	121418033	121491679	73646	0.23
4	122001332	122004374	3042	0.06
4	124044107	124045592	1485	0.23
4	124293356	124300695	7339	0.06
4	126392351	126395215	2864	0.06
4	126445197	126449999	4802	0.06
4	128750763	128759100	8337	0.06
4	128923137	128928102	4965	0.06
4	129630448	129634823	4375	0.06
4	131996534	132024693	28159	0.52
4	132185584	132199536	13952	0.06
4	133349760	133372757	22997	0.06
4	133790519	133833400	42881	0.17
4	134544175	134659501	115326	0.17
4	134905734	134915051	9317	0.23
4	135091373	135097568	6195	0.06
4	135587430	135590244	2814	0.29
4	135674909	135680789	5880	0.06
4	135724321	135728887	4566	0.06
4	136005088	136046012	40924	0.06
4	136349126	136397242	48116	0.11

4	136438762	136451448	12686	0.34
4	138311075	138313926	2851	0.06
4	141204226	141215611	11385	0.11
4	143832994	144093819	260825	4.07
4	144780273	144785210	4937	0.06
4	145285572	145391948	106376	0.40
4	146047752	146054988	7236	0.17
4	146545906	146547291	1385	0.06
4	149722804	149782678	59874	0.11
4	152058098	152064205	6107	0.06
4	153091334	153095992	4658	0.06
4	153494032	153506500	12468	0.06
4	155086509	155090483	3974	0.34
4	156326385	156328651	2266	0.11
4	156484072	156587692	103620	0.11
4	156914334	156921244	6910	0.06
4	157619176	157622061	2885	0.06
4	157700370	157720743	20373	0.57
4	160128568	160150870	22302	0.34
4	160169463	160189795	20332	0.17
4	160239088	160244984	5896	0.11
4	160354926	160361355	6429	0.06
4	160862071	160870982	8911	0.06
4	161300232	161302835	2603	0.06
4	161589661	161599289	9628	0.06
4	161627664	161643231	15567	0.17
4	161692625	161708470	15845	0.06
4	162351166	162357156	5990	0.34
4	164655303	164666188	10885	0.06
4	164774280	164777683	3403	0.06
4	164785832	164795749	9917	0.29
4	165068498	165124404	55906	0.34
4	165547452	165581517	34065	0.11
4	166398658	166462447	63789	0.06
4	166577973	166589782	11809	0.06
4	167535578	167540874	5296	0.06
4	167869309	168071368	202059	0.29
4	168792746	168797697	4951	0.06
4	170047931	170054053	6122	0.06
4	171211675	171221721	10046	0.06
4	171807843	171816979	9136	0.06
4	172379674	172426342	46668	0.06
4	173471356	173498820	27464	0.17
4	173545731	173554947	9216	0.17
4	173896559	173905006	8447	0.06
4	174609314	174614257	4943	0.06

4	174984735	175103274	118539	0.06
4	177547006	177565451	18445	0.11
4	178040647	178047520	6873	0.06
4	179038059	179042420	4361	0.06
4	180212643	180217600	4957	0.11
4	181261499	181264797	3298	0.06
4	182054067	182058201	4134	0.06
4	182154522	182174533	20011	0.11
4	182364858	182374924	10066	0.11
4	184183318	184189761	6443	0.06
4	184811408	184816161	4753	0.11
4	184832200	184839680	7480	0.34
4	185477338	185498735	21397	0.06
4	186446056	186447310	1254	0.06
4	186921989	186929178	7189	1.84
4	187228564	187239773	11209	0.17
4	187288028	187309547	21519	0.11
4	187790575	187793051	2476	0.17
4	188023891	188025074	1183	0.06
4	188657825	188665453	7628	0.06
4	189225350	189229108	3758	0.06
4	189486816	189533075	46259	0.06
5	537222	542289	5067	0.17
5	661741	873886	212145	2.81
5	1052245	1093494	41249	0.57
5	1175988	1187474	11486	0.17
5	1662069	1677112	15043	0.06
5	1686118	1688112	1994	0.23
5	1841227	1852170	10943	0.06
5	3346139	3349469	3330	0.06
5	3693799	3698825	5026	0.06
5	4024526	4034431	9905	0.06
5	4159456	4167400	7944	0.06
5	5156542	5168366	11824	0.06
5	6551547	6553372	1825	0.06
5	6632182	6645034	12852	0.06
5	6792152	6795762	3610	0.06
5	6984313	6987091	2778	0.11
5	7178531	7200737	22206	0.34
5	8591521	8597734	6213	0.23
5	9756217	9770223	14006	0.06
5	10794874	10797906	3032	0.06
5	11448556	11459171	10615	0.17
5	11873554	11879797	6243	0.06
5	12584431	12669624	85193	0.29
5	12687505	12756843	69338	0.29

5	13275096	13280689	5593	0.06
5	13359366	13382837	23471	0.11
5	16006130	16017804	11674	0.34
5	17318885	17321598	2713	0.06
5	17429496	17434500	5004	0.06
5	17939163	17945922	6759	0.06
5	17960987	17989180	28193	0.11
5	18007118	19015970	1008852	0.40
5	19280415	19286859	6444	0.69
5	19545165	19548252	3087	0.06
5	19605057	19614028	8971	0.06
5	19957277	19978017	20740	0.57
5	20240944	20262348	21404	0.29
5	22223236	22239112	15876	0.06
5	22556143	22573221	17078	0.06
5	23049000	23053079	4079	0.17
5	23578332	23598332	20000	0.06
5	24048775	24081870	33095	0.06
5	25424618	25446648	22030	0.06
5	25910615	26020006	109391	0.06
5	26348036	26356918	8882	0.06
5	26906750	26913135	6385	0.06
5	27099320	27153148	53828	0.06
5	28256342	28500607	244265	0.29
5	29596352	29624532	28180	0.11
5	30004495	30009526	5031	0.06
5	30110718	30122083	11365	0.06
5	30583082	30589514	6432	0.11
5	36070960	36074140	3180	0.06
5	36546199	36554107	7908	0.06
5	40074640	40081810	7170	0.06
5	40115231	40122096	6865	0.06
5	40176002	40184510	8508	0.06
5	40336444	40351447	15003	0.52
5	40767295	40779589	12294	0.11
5	41448085	41458135	10050	0.06
5	42590384	42603352	12968	0.06
5	42638320	42643098	4778	0.06
5	44189795	44196647	6852	0.06
5	44899983	44919855	19872	0.06
5	51972930	51979594	6664	0.06
5	55918030	55925051	7021	0.11
5	56111125	56122165	11040	0.06
5	57003385	57005440	2055	0.06
5	57192338	57228587	36249	0.29
5	57422028	57432182	10154	0.06

5	57623336	57626488	3152	0.06
5	58355547	58359069	3522	0.06
5	61277352	61302716	25364	0.17
5	63886301	63899120	12819	0.06
5	65303174	65379406	76232	0.06
5	67342569	67352045	9476	0.06
5	69191229	69282601	91372	0.17
5	76372663	76376693	4030	0.11
5	79125957	79215563	89606	3.27
5	79372535	79387480	14945	0.29
5	79760518	79766425	5907	0.06
5	80265085	80276188	11103	0.06
5	80830158	80871510	41352	0.11
5	81202560	81204295	1735	0.23
5	81269668	81296457	26789	0.06
5	82435614	82519512	83898	0.40
5	84747973	84768324	20351	0.06
5	85094176	85114848	20672	0.06
5	85354068	85419065	64997	0.11
5	86971122	87057004	85882	0.17
5	90065665	90072975	7310	0.06
5	93430213	93436727	6514	0.06
5	93636172	93646340	10168	0.06
5	97367538	98689505	1321967	1.66
5	98753579	98761031	7452	0.29
5	99987974	100055023	67049	0.06
5	100553722	100587806	34084	0.11
5	101833530	101870665	37135	0.40
5	104107316	104117228	9912	0.17
5	104172643	104201262	28619	0.40
5	104362408	104446262	83854	0.06
5	104622750	104638857	16107	0.11
5	104870579	104996732	126153	0.23
5	105097815	105168044	70229	0.29
5	106276414	106280838	4424	0.11
5	106554601	106577225	22624	0.11
5	106890089	106898113	8024	0.52
5	107361322	107365793	4471	0.29
5	108834906	108888191	53285	0.11
5	108951434	108991796	40362	0.17
5	110024841	110039779	14938	0.06
5	110416853	110443223	26370	0.06
5	112013623	112030052	16429	0.11
5	112290835	112347635	56800	0.17
5	112934564	112941755	7191	0.11
5	113620897	113646891	25994	0.17

5	113816844	113836766	19922	1.43
5	113991787	113999533	7746	0.23
5	114245738	114248895	3157	0.06
5	114259261	114264647	5386	0.06
5	114677683	114680008	2325	0.11
5	115067757	115072691	4934	0.06
5	116872257	116874652	2395	0.11
5	116951528	116967982	16454	0.06
5	117976748	118002366	25618	0.06
5	119300538	119306332	5794	0.06
5	120029759	120106037	76278	0.11
5	120304531	120307928	3397	0.06
5	120588837	120590610	1773	0.06
5	120725334	120737551	12217	0.06
5	120804905	120845039	40134	0.11
5	121350603	121370202	19599	0.23
5	121919079	121924912	5833	0.06
5	123287268	123288507	1239	0.06
5	123475626	123485369	9743	0.17
5	126159007	126171614	12607	0.63
5	127109951	127120090	10139	0.11
5	128879045	128885806	6761	0.06
5	129291662	129295132	3470	0.11
5	130738468	130781661	43193	0.11
5	132693008	132708607	15599	0.11
5	132989674	133025211	35537	0.06
5	134596037	134736501	140464	0.97
5	135445219	135448156	2937	0.11
5	135670775	135679910	9135	0.11
5	136535514	136540364	4850	0.06
5	136551466	136582278	30812	0.06
5	138441242	138454889	13647	0.23
5	138725652	138762993	37341	0.46
5	140413110	140504522	91412	0.23
5	140929431	140942008	12577	0.06
5	141097310	141101891	4581	0.11
5	141188399	141191953	3554	0.11
5	141341387	141345024	3637	0.23
5	142120871	142150782	29911	0.06
5	143849253	143851230	1977	0.11
5	144014500	144023551	9051	0.06
5	144135070	144139762	4692	0.06
5	144148409	144161181	12772	0.11
5	144518625	144520288	1663	0.06
5	144675269	144682743	7474	0.06
5	145933370	145935315	1945	0.06

5	146132805	146182719	49914	0.06
5	146409543	146417922	8379	0.11
5	146550981	146558898	7917	0.06
5	147260623	147264009	3386	0.06
5	147312447	147318885	6438	0.06
5	147349399	147357904	8505	0.06
5	147797762	147805157	7395	0.34
5	149636555	149640603	4048	0.06
5	150116758	150124595	7837	0.06
5	151020073	151023009	2936	0.06
5	151975081	152001438	26357	0.06
5	152773633	152784200	10567	0.06
5	155024616	155028460	3844	0.06
5	157033084	157052193	19109	0.29
5	158218325	158228240	9915	0.06
5	162372456	162383343	10887	0.06
5	163131669	163136905	5236	0.11
5	163588237	163596967	8730	0.06
5	163747690	163755444	7754	0.80
5	165295522	165298768	3246	0.75
5	165365075	165373662	8587	0.06
5	166340689	166352908	12219	0.11
5	170450803	170453067	2264	0.06
5	170721011	170742121	21110	0.06
5	171642848	171664463	21615	0.11
5	173262829	173265345	2516	0.06
5	173667148	173672181	5033	0.11
5	174739342	174743179	3837	0.06
5	174870583	174876400	5817	0.06
5	175046828	175049461	2633	0.06
5	175883811	175891232	7421	0.11
5	176728959	176736805	7846	0.06
5	176835223	176880962	45739	0.52
5	177944712	177974508	29796	1.03
5	177985488	178002696	17208	0.06
5	178065631	178071979	6348	0.06
5	178536523	178537788	1265	0.11
5	178768588	178775309	6721	0.06
5	179126018	179140021	14003	0.11
5	179297794	179303061	5267	0.06
5	179773896	179804248	30352	0.17
6	902934	911285	8351	0.06
6	1993750	2005994	12244	0.06
6	3693987	3711111	17124	0.11
6	4170590	4174271	3681	0.17
6	4692663	4697259	4596	0.06

6	5167360	5510129	342769	0.29
6	6172069	6176811	4742	0.06
6	6963726	6965604	1878	0.06
6	9495536	9519108	23572	0.06
6	10152776	10177380	24604	0.06
6	10497171	10542893	45722	0.06
6	11798245	11918585	120340	0.06
6	12210897	12213464	2567	0.06
6	15762117	15774010	11893	0.06
6	16568561	16573598	5037	0.11
6	17229572	17252111	22539	0.11
6	19227627	19245135	17508	0.23
6	20581597	20589110	7513	0.06
6	21827479	21832315	4836	0.23
6	23609043	23623655	14612	0.23
6	23811997	23816462	4465	0.06
6	26282094	26295463	13369	0.11
6	26344105	26352025	7920	3.44
6	26614056	26619100	5044	0.46
6	27240386	27247211	6825	0.06
6	27330850	27338739	7889	0.11
6	27684784	27691413	6629	0.06
6	28050416	28061465	11049	0.23
6	28419666	28424457	4791	0.06
6	28700703	28719042	18339	0.80
6	28748568	28764737	16169	0.17
6	28856620	28860233	3613	0.06
6	29031986	29042376	10390	0.52
6	29517512	29534259	16747	1.09
6	29889788	29931412	41624	3.33
6	30782905	30792404	9499	0.06
6	31025813	31029915	4102	0.06
6	31387541	31488621	101080	1.84
6	31614248	31618317	4069	0.06
6	31833091	31839763	6672	0.06
6	32749447	32752812	3365	0.06
6	33995724	34000578	4854	0.11
6	38171706	38173546	1840	0.06
6	38209891	38218629	8738	0.06
6	38870847	38873325	2478	0.06
6	39190089	39195069	4980	0.06
6	39325801	39328887	3086	0.06
6	40395655	40401121	5466	0.57
6	42183647	42185910	2263	0.06
6	42537378	42631935	94557	0.23
6	42812727	42862843	50116	0.11

6	44894997	44898585	3588	0.06
6	48567251	48611491	44240	0.06
6	52767229	52804387	37158	0.06
6	52925013	52930135	5122	0.06
6	53990706	54001558	10852	0.23
6	55788116	55796080	7964	0.06
6	56363288	56366925	3637	0.06
6	60383189	60395845	12656	0.06
6	63220704	63238458	17754	0.17
6	64640883	64656671	15788	0.06
6	65102147	65354367	252220	0.11
6	65700825	65705727	4902	0.06
6	66238936	66726868	487932	2.01
6	67072567	67084552	11985	0.06
6	68019238	68021821	2583	0.06
6	69504952	69515019	10067	0.06
6	70889615	70891807	2192	0.11
6	71447526	71450697	3171	0.11
6	71987321	71989810	2489	0.06
6	73387004	73399746	12742	0.06
6	73447894	73529092	81198	0.11
6	73536669	73549323	12654	0.06
6	73957565	73975267	17702	0.06
6	74122681	74123762	1081	0.17
6	74383321	74387332	4011	0.06
6	76853349	76878407	25058	0.17
6	77124398	77144466	20068	0.63
6	77616376	77624914	8538	0.06
6	78149819	78467228	317409	3.21
6	80489109	80508195	19086	0.06
6	80575520	80584120	8600	1.61
6	80794111	80805406	11295	0.11
6	81587173	81659962	72789	0.06
6	83519395	83522280	2885	0.06
6	87668196	87693993	25797	0.06
6	88412775	88433272	20497	0.06
6	88983718	89001975	18257	0.06
6	90646196	90652393	6197	0.06
6	92710704	92718333	7629	0.06
6	92820218	92824665	4447	0.06
6	92878116	92889133	11017	0.06
6	94225590	94231662	6072	0.06
6	94583307	94636049	52742	0.11
6	94763972	94923583	159611	0.11
6	97731610	97743512	11902	0.06
6	99956729	99962241	5512	0.06

6	102717218	102832775	115557	0.17
6	103552569	103556472	3903	0.06
6	103675469	103700308	24839	0.06
6	104462210	104487762	25552	0.06
6	104725241	104869061	143820	0.34
6	105186804	105199916	13112	0.06
6	107135643	107177000	41357	0.11
6	107776211	107782259	6048	0.06
6	110075337	110091553	16216	0.52
6	112321323	112322466	1143	0.17
6	113579449	113582033	2584	0.06
6	116326406	116328587	2181	0.11
6	117756414	117763184	6770	0.52
6	125162408	125169709	7301	0.06
6	126657671	126703978	46307	0.06
6	128750646	128772127	21481	0.06
6	129418302	129425960	7658	0.17
6	129486484	129489364	2880	0.06
6	129611643	129633972	22329	0.46
6	129714667	129720744	6077	0.46
6	132600101	132604474	4373	0.34
6	136486013	136508794	22781	0.06
6	136794748	136813866	19118	0.06
6	140066616	140069969	3353	0.11
6	140406508	140413832	7324	0.06
6	140455086	140459940	4854	0.06
6	140543421	140596593	53172	0.06
6	141370404	141388285	17881	0.11
6	141573091	141589919	16828	0.11
6	141640911	141875365	234454	0.69
6	141938035	141962061	24026	0.06
6	142650034	142652702	2668	0.23
6	144821373	144824770	3397	0.29
6	146627370	146705287	77917	0.11
6	147179348	147183827	4479	0.06
6	149753911	149792036	38125	0.23
6	149931160	149949450	18290	0.06
6	151005819	151007201	1382	0.23
6	151063827	151071137	7310	0.06
6	151297100	151302345	5245	0.06
6	151979492	151989333	9841	0.57
6	153464352	153475941	11589	0.23
6	155250202	155289290	39088	0.06
6	155785980	155806677	20697	0.06
6	158740396	158757041	16645	0.06
6	160482146	160486434	4288	0.63

6	162297190	162329478	32288	0.11
6	162396542	162414356	17814	0.06
6	162562811	162659326	96515	0.11
6	163373474	163376743	3269	0.06
6	165382401	165385747	3346	0.06
6	166693660	166741586	47926	0.06
6	167268243	167314230	45987	0.06
6	167322466	167359267	36801	0.06
6	167745609	167758721	13112	0.06
6	167989168	167993206	4038	0.29
6	168040800	168093484	52684	1.26
6	168590686	168598990	8304	0.23
6	169106742	169120465	13723	0.11
7	991481	1012717	21236	0.11
7	1108367	1129983	21616	0.06
7	1185232	1223395	38163	0.11
7	1255586	1289071	33485	0.34
7	1537068	1562800	25732	0.17
7	1738406	1751712	13306	0.23
7	2001609	2016690	15081	0.40
7	2629191	2666844	37653	0.34
7	2875744	2887938	12194	0.06
7	2905708	2907430	1722	0.06
7	3348364	3352241	3877	0.11
7	3354836	3412970	58134	0.06
7	3571976	3578511	6535	0.46
7	3630881	3635650	4769	0.06
7	3676035	3687895	11860	0.06
7	3866057	3873656	7599	0.06
7	4099277	4101082	1805	0.06
7	4299782	4301317	1535	0.06
7	4315576	4495828	180252	1.20
7	4558414	4575575	17161	0.34
7	4783328	4804237	20909	0.92
7	5137374	5150247	12873	0.06
7	5988559	6007152	18593	0.11
7	6209299	6232553	23254	0.06
7	6289315	6370897	81582	0.40
7	7287120	7353766	66646	0.06
7	7884653	7885966	1313	0.11
7	9033418	9042935	9517	0.06
7	9257624	9262682	5058	0.40
7	9593325	9596844	3519	0.11
7	9614276	9618269	3993	0.23
7	9828799	9831380	2581	0.06
7	10074193	10093784	19591	0.06

7	10205790	10263997	58207	0.11
7	11299546	11361901	62355	0.11
7	11668033	11673573	5540	0.11
7	12350561	12393569	43008	0.11
7	13029202	13035492	6290	0.06
7	13068725	13090051	21326	2.29
7	13275130	13277065	1935	0.06
7	13545049	13575780	30731	0.06
7	13841161	13846099	4938	0.29
7	13853049	13879209	26160	0.06
7	14141391	14162680	21289	0.06
7	14469683	14474919	5236	0.06
7	14514921	14529220	14299	0.06
7	14710955	14721747	10792	0.11
7	15132349	15134859	2510	0.06
7	15239863	15244407	4544	0.06
7	16222610	16373887	151277	0.17
7	17470600	17473964	3364	0.06
7	17530855	17635952	105097	0.06
7	18296669	18308034	11365	0.06
7	19051984	19095587	43603	0.11
7	20267888	20288064	20176	3.10
7	22003001	22007544	4543	0.06
7	22743170	22755534	12364	0.06
7	22774123	22794012	19889	0.06
7	24058110	24074437	16327	0.11
7	25313113	25333518	20405	0.06
7	25360111	25375572	15461	0.06
7	26577050	26579861	2811	0.17
7	26738787	26763444	24657	0.06
7	28356110	28365779	9669	0.06
7	28987022	28995737	8715	0.29
7	29054969	29059564	4595	0.06
7	29636481	29744895	108414	0.92
7	29823519	29827466	3947	0.11
7	30451695	30456758	5063	0.11
7	30850001	30873404	23403	0.17
7	31383170	31386069	2899	0.06
7	33178093	33197877	19784	0.06
7	36861974	36869263	7289	0.11
7	37731368	37851005	119637	0.34
7	38186533	38196002	9469	0.06
7	38256357	38338702	82345	10.03
7	38349716	38377955	28239	0.06
7	38607291	38613193	5902	0.06
7	40225809	40246029	20220	0.06

7	40271638	40274754	3116	0.11
7	41473344	41474698	1354	0.40
7	44763687	44768817	5130	0.06
7	47143093	47145485	2392	0.06
7	51976058	51981065	5007	0.17
7	53207019	53211442	4423	0.06
7	53586309	53649018	62709	0.06
7	54762510	54768791	6281	0.06
7	55811496	55853114	41618	0.17
7	56198735	56211388	12653	0.06
7	56544583	56558320	13737	0.34
7	57176453	57195560	19107	0.06
7	57387311	57395936	8625	0.06
7	62742653	62997884	255231	4.42
7	63252691	63268846	16155	0.17
7	64234704	64243653	8949	0.06
7	64268823	64277609	8786	0.06
7	64446888	64621160	174272	0.06
7	64702947	64750963	48016	0.29
7	65045230	65059834	14604	0.06
7	65984729	66056240	71511	0.06
7	66638105	66647208	9103	0.06
7	67358035	67399305	41270	0.06
7	68191556	68201186	9630	0.57
7	68537021	68540854	3833	0.06
7	70447221	70452025	4804	0.06
7	70866248	70878095	11847	0.52
7	72262936	72305492	42556	0.06
7	72500699	72517939	17240	0.57
7	74009516	74066239	56723	0.29
7	74115646	74154095	38449	0.11
7	74205625	74253943	48318	0.17
7	74323737	74357419	33682	0.17
7	74393989	74446388	52399	0.63
7	75765166	75815587	50421	0.40
7	78443163	78446245	3082	0.11
7	78499655	78520971	21316	0.06
7	78692748	78694533	1785	0.06
7	79274493	79281080	6587	0.06
7	79863894	79875114	11220	0.06
7	80331775	80336864	5089	0.06
7	80986541	80991146	4605	0.06
7	81471211	81477958	6747	0.17
7	83493602	83499168	5566	0.11
7	85400976	85423179	22203	0.29
7	85449699	85475288	25589	0.11

7	85882236	85893338	11102	0.06
7	86771959	86780457	8498	0.06
7	90162845	90171041	8196	0.06
7	91958722	92039540	80818	0.23
7	93592090	93624851	32761	0.06
7	100240611	100307211	66600	0.34
7	100755214	100786529	31315	0.06
7	101779421	101785228	5807	0.11
7	105326396	105356575	30179	0.06
7	105426782	105462577	35795	0.34
7	105473100	105476012	2912	0.06
7	106176858	106179918	3060	0.06
7	106421043	106422167	1124	0.06
7	107468612	107475407	6795	0.11
7	108862455	108904752	42297	0.06
7	109442967	109464142	21175	0.75
7	109816478	109822243	5765	0.06
7	110632371	110671861	39490	0.06
7	110846352	110879734	33382	0.06
7	111140642	111443435	302793	2.47
7	111738162	111789826	51664	0.06
7	112303547	112320917	17370	0.17
7	112343661	112350577	6916	0.06
7	113666922	113761488	94566	0.06
7	114635514	114651380	15866	0.11
7	115405540	115437139	31599	0.06
7	115476249	115484286	8037	0.06
7	118487954	118530461	42507	2.29
7	118666685	118671460	4775	0.06
7	122123669	122178700	55031	0.06
7	123680381	123685654	5273	0.17
7	125236132	125250310	14178	0.06
7	125277987	125295173	17186	0.06
7	125539465	125556916	17451	0.06
7	125848374	125853906	5532	0.06
7	127161935	127166678	4743	0.11
7	129766882	129778156	11274	0.11
7	130216351	130221866	5515	0.06
7	134538966	134573524	34558	0.06
7	135522216	135530649	8433	0.06
7	135678297	135681044	2747	0.23
7	136381362	136397415	16053	0.29
7	137128719	137133266	4547	0.06
7	138195102	138205280	10178	0.06
7	141986407	141998026	11619	0.06
7	142129271	142141614	12343	0.06

7	143128657	143193775	65118	0.34
7	143387481	143396731	9250	0.11
7	144084303	144113806	29503	0.23
7	145064291	145074478	10187	0.17
7	146096868	146102035	5167	0.52
7	146534616	146557416	22800	0.11
7	148151055	148154165	3110	0.06
7	148934474	148947457	12983	1.95
7	149598842	149618038	19196	0.06
7	149668212	149671621	3409	0.11
7	150351675	150356717	5042	0.06
7	150538576	150542588	4012	0.06
7	150610251	150622590	12339	0.06
7	150751785	150758207	6422	0.06
7	150856387	150865114	8727	0.46
7	150868863	150875668	6805	0.17
7	152690784	152700845	10061	0.17
7	153497075	153509603	12528	1.20
7	154711618	154723732	12114	0.17
7	155093527	155097474	3947	1.49
7	155145324	155156283	10959	0.06
7	155166389	155189056	22667	0.57
7	155265157	155287886	22729	0.06
7	155569233	155571547	2314	0.06
7	156530238	156541965	11727	0.11
7	156840985	156850466	9481	0.06
7	156902733	156908494	5761	0.23
7	157277234	157284746	7512	0.06
7	157421025	157429029	8004	0.34
7	157809785	157813447	3662	0.17
7	158149215	158154214	4999	0.06
7	158515597	158537228	21631	0.06
7	158595319	158623511	28192	0.23
7	158677524	158683456	5932	0.23
7	158706303	158729046	22743	0.11
8	981580	1020267	38687	0.06
8	1066903	1106870	39967	0.29
8	1348873	1350184	1311	0.17
8	1356997	1358754	1757	0.17
8	1724613	1726632	2019	0.17
8	2148982	2156817	7835	0.06
8	2158771	2211989	53218	0.57
8	2731282	2733490	2208	0.11
8	3029873	3036511	6638	0.63
8	3257643	3270577	12934	0.11
8	3348940	3352569	3629	0.06

8	3396246	3402609	6363	0.11
8	3659270	3664857	5587	0.06
8	4009734	4017975	8241	0.06
8	4056077	4063472	7395	0.06
8	4110409	4112015	1606	0.17
8	4168284	4176385	8101	0.46
8	4290472	4300260	9788	0.06
8	4423415	4463888	40473	0.06
8	4617952	4630893	12941	0.57
8	4653081	4657165	4084	0.40
8	4725196	4732176	6980	0.11
8	5028794	5038475	9681	0.06
8	5050187	5057024	6837	0.06
8	5213748	5218567	4819	0.23
8	5571568	5573175	1607	0.06
8	6101073	6126814	25741	2.01
8	6294339	6296791	2452	0.17
8	6314862	6351904	37042	0.40
8	6499416	6507401	7985	0.06
8	6838495	6841108	2613	0.17
8	7154036	7785129	631093	0.40
8	8722087	8772426	50339	0.23
8	9852174	9860768	8594	0.06
8	11130043	11134371	4328	0.23
8	11842178	11843741	1563	0.23
8	11916380	11930179	13799	0.11
8	13619440	13626521	7081	0.11
8	13652018	13756130	104112	2.24
8	13795213	13838525	43312	0.29
8	14559618	14560822	1204	0.06
8	14651084	14658468	7384	0.46
8	14689850	14718386	28536	0.06
8	15562268	15575144	12876	0.17
8	15920498	15929380	8882	0.06
8	15958485	15961693	3208	0.63
8	16234177	16249301	15124	0.11
8	16955323	16968788	13465	0.23
8	17517451	17521389	3938	0.11
8	20030532	20033265	2733	0.06
8	20273934	20279271	5337	0.06
8	21625742	21632426	6684	0.06
8	22648895	22650543	1648	0.06
8	23010653	23018472	7819	0.06
8	23163943	23171633	7690	0.06
8	23328054	23337415	9361	0.06
8	24755010	24792886	37876	0.06

8	25138018	25153837	15819	0.06
8	26738756	26745756	7000	0.06
8	27786996	27837730	50734	0.34
8	28013819	28015631	1812	0.17
8	28528677	28551083	22406	0.06
8	28755207	28868516	113309	0.06
8	29296260	29314465	18205	0.06
8	29347956	29354552	6596	0.11
8	32624155	32639729	15574	0.06
8	33649392	33672581	23189	0.17
8	34229450	34232152	2702	0.06
8	34979499	34996980	17481	0.23
8	38531112	38543517	12405	0.06
8	39530806	39575057	44251	0.57
8	39939939	39942187	2248	0.06
8	40236785	40247392	10607	0.06
8	41373362	41394768	21406	0.17
8	48671213	48674415	3202	0.06
8	49961335	50047950	86615	0.11
8	51126804	51164696	37892	0.06
8	51342579	51347820	5241	0.06
8	51970749	52000640	29891	0.06
8	53050218	53079475	29257	0.29
8	54533052	54537711	4659	0.06
8	55076481	55092356	15875	0.06
8	55697708	55705643	7935	0.06
8	55822561	55855194	32633	0.17
8	56320228	56349376	29148	0.06
8	57361011	57435874	74863	0.11
8	57442391	57454399	12008	1.20
8	61125450	61138050	12600	0.11
8	62935739	62938350	2611	0.06
8	64019391	64068198	48807	0.06
8	64201538	64211314	9776	0.06
8	64329609	64343492	13883	0.40
8	65949957	65954892	4935	0.06
8	68474725	68483386	8661	0.06
8	68880306	68888591	8285	0.06
8	68959626	68963342	3716	0.11
8	69101020	69112887	11867	0.06
8	69523630	69541416	17786	0.11
8	69913749	69936479	22730	0.11
8	69945636	69951659	6023	0.17
8	69958621	69977897	19276	0.06
8	73091561	73107956	16395	0.06
8	73754834	73775124	20290	0.34

8	73994494	73997501	3007	0.06
8	75183908	75198815	14907	0.11
8	75369081	75375177	6096	0.80
8	79146659	79170429	23770	0.06
8	79974940	79991372	16432	0.06
8	82455336	82505666	50330	0.06
8	82575960	82590515	14555	0.11
8	83569443	83733919	164476	0.06
8	83893419	83895573	2154	0.06
8	85331941	85348562	16621	0.06
8	85581363	85638247	56884	0.06
8	87372393	87420409	48016	0.06
8	87531678	87540883	9205	0.06
8	90919028	90925092	6064	0.06
8	93474648	93483277	8629	0.06
8	93634713	93641979	7266	0.06
8	96021609	96024415	2806	0.06
8	97743206	97766059	22853	0.23
8	97876925	97892501	15576	0.06
8	100639811	100644478	4667	0.06
8	100793919	100798618	4699	0.06
8	101375563	101391178	15615	0.40
8	102692268	102702998	10730	0.06
8	103802073	103826332	24259	0.11
8	104166011	104188506	22495	0.52
8	104712619	104723542	10923	0.06
8	109413035	109429113	16078	0.23
8	111786402	111789985	3583	0.11
8	112299542	112370163	70621	0.11
8	113792132	113795845	3713	0.06
8	114459900	114502591	42691	0.69
8	116319980	116322335	2355	0.06
8	118406037	118413471	7434	0.06
8	118676085	118682873	6788	0.40
8	121352478	121377669	25191	0.06
8	128216815	128221552	4737	0.11
8	128580289	128608229	27940	0.11
8	129101694	129119118	17424	0.17
8	129961819	129969662	7843	0.06
8	134113303	134115750	2447	0.06
8	134991415	134994387	2972	0.23
8	136673185	136849837	176652	1.20
8	137055377	137069047	13670	0.23
8	139546570	139577199	30629	0.11
8	140366882	140377822	10940	0.06
8	140613722	140617217	3495	0.06

8	141012587	141049737	37150	0.06
8	141345643	141439595	93952	0.52
8	141474266	141492234	17968	0.17
8	141861270	141875272	14002	0.23
8	141993744	142025210	31466	0.11
8	142092622	142107190	14568	0.06
8	142522314	142537523	15209	0.63
8	143257962	143276889	18927	0.23
8	143427962	143466636	38674	0.06
8	143489261	143607119	117858	0.40
8	143904185	143957097	52912	0.52
8	143989660	144012715	23055	0.06
8	144040026	144105743	65717	0.23
8	144278821	144322373	43552	0.11
8	144383278	144458398	75120	0.40
8	144493267	144533863	40596	0.23
9	610771	705205	94434	0.34
9	790256	793816	3560	0.06
9	1189365	1197716	8351	0.06
9	1269555	1271596	2041	0.06
9	1427380	1428973	1593	0.06
9	2209802	2212574	2772	0.06
9	2403909	2421690	17781	0.06
9	2695208	2697695	2487	0.11
9	2840829	2848800	7971	0.06
9	3741545	3763952	22407	0.06
9	4362167	4385225	23058	0.06
9	4455607	4458374	2767	0.06
9	4671409	4793033	121624	0.11
9	5108334	5130363	22029	0.06
9	5304951	5337542	32591	0.86
9	5674203	5693620	19417	0.29
9	6399107	6401105	1998	0.06
9	6610588	6641859	31271	0.06
9	7732981	7772350	39369	0.11
9	8905119	9032105	126986	0.11
9	9878200	10010764	132564	0.29
9	10081387	10091133	9746	0.06
9	10170874	10180371	9497	0.06
9	10279866	10285562	5696	0.23
9	10371281	10374460	3179	0.06
9	10546814	10592572	45758	0.29
9	10704801	10710144	5343	0.06
9	10909144	10936687	27543	0.06
9	11155468	11160884	5416	0.06
9	11353760	11381692	27932	0.06

9	11640367	12182768	542401	2.70
9	12298181	12456576	158395	0.29
9	12494501	12565444	70943	0.06
9	12727189	12749459	22270	0.06
9	13791532	13803516	11984	0.06
9	13815722	13887707	71985	0.11
9	15304437	15405298	100861	0.11
9	16907143	16912535	5392	0.06
9	17307206	17334077	26871	0.11
9	17402131	17406621	4490	0.57
9	17810305	17900506	90201	0.06
9	18589750	18595550	5800	0.06
9	19253270	19336325	83055	0.40
9	19736873	19740885	4012	0.06
9	19861137	19875940	14803	0.11
9	20669941	20681079	11138	0.06
9	21193861	21226833	32972	0.57
9	21599221	21610098	10877	0.06
9	22364081	22366793	2712	0.06
9	22572194	22594968	22774	0.11
9	22923906	22929104	5198	0.17
9	23125852	23159742	33890	0.06
9	23290143	23299153	9010	0.11
9	23935424	23948630	13206	0.06
9	24329958	24343371	13413	0.11
9	25249934	25352203	102269	0.57
9	25559758	25584046	24288	0.17
9	26275966	26305948	29982	0.06
9	26343790	26357955	14165	0.11
9	26550672	26562766	12094	0.06
9	26874345	26885992	11647	0.17
9	27800009	27807541	7532	0.17
9	28183433	28195208	11775	0.11
9	28309790	28348135	38345	0.17
9	28542805	28661684	118879	0.34
9	28672394	28776754	104360	1.32
9	29539149	29544134	4985	0.06
9	30175190	30310100	134910	0.11
9	31405319	31453234	47915	0.11
9	32170495	32172342	1847	0.11
9	33462097	33467985	5888	0.11
9	33845081	33914360	69279	0.46
9	35982824	35993379	10555	0.11
9	36135427	36147874	12447	0.06
9	37394545	37396261	1716	0.06
9	38386354	38389540	3186	0.06

9	66894081	66938489	44408	0.06
9	68746911	68750097	3186	0.06
9	71151555	71155980	4425	0.23
9	71870540	71872536	1996	0.11
9	72064358	72089308	24950	0.46
9	74888019	74902569	14550	0.06
9	76535611	76545077	9466	0.11
9	77261966	77321718	59752	0.06
9	77458300	77463600	5300	0.06
9	77595834	77603316	7482	0.06
9	78097650	78103095	5445	0.06
9	78823393	78850088	26695	0.17
9	79034093	79038982	4889	0.06
9	85724372	85727723	3351	0.06
9	89310312	89312318	2006	0.06
9	92965601	92975367	9766	0.29
9	93113165	93138214	25049	0.11
9	97321004	97328667	7663	0.06
9	101899463	101910706	11243	0.11
9	102017404	102019814	2410	0.06
9	102349016	102366836	17820	0.46
9	102669637	102678641	9004	0.17
9	103005945	103023251	17306	1.20
9	103084214	103111246	27032	0.29
9	103616197	103627049	10852	0.17
9	103779694	103793832	14138	0.06
9	103954708	103958400	3692	1.32
9	104610357	104622668	12311	0.46
9	110021284	110034605	13321	0.06
9	111261915	111272550	10635	0.11
9	111702207	111707858	5651	1.09
9	112248160	112258135	9975	0.06
9	112894766	112900864	6098	0.06
9	113003798	113009320	5522	0.11
9	114247463	114265429	17966	0.11
9	115017389	115019496	2107	0.06
9	115310805	115321587	10782	0.17
9	116746516	116749852	3336	0.06
9	118415803	118494757	78954	0.11
9	119530014	119542304	12290	0.17
9	119880516	119886403	5887	0.06
9	122566586	122568460	1874	0.17
9	123416415	123435342	18927	0.06
9	124162723	124165294	2571	0.06
9	124836861	124857643	20782	0.40
9	125420699	125438143	17444	0.52

9	126699041	126706307	7266	0.11
9	126777727	126785408	7681	0.06
9	127549801	127558278	8477	0.06
9	129287563	129292175	4612	0.06
9	129769341	129779207	9866	0.34
9	130886229	130906229	20000	0.75
9	132439243	132447209	7966	0.06
9	133034308	133038096	3788	0.23
9	133708289	133736268	27979	0.34
9	133763269	133810892	47623	0.40
9	133951218	133981075	29857	0.34
9	134029242	134033114	3872	0.11
9	134233655	134239683	6028	0.06
9	134340689	134376145	35456	0.06
9	134706412	134710273	3861	0.52
9	134750731	134786460	35729	0.92
9	134848652	134855500	6848	0.06
9	134966572	135003508	36936	0.17
9	135243499	135258742	15243	0.11
9	135384508	135402312	17804	0.63
9	135680873	135705128	24255	0.11
9	135797454	135800141	2687	0.11
9	136250450	136266613	16163	0.11
9	139165990	139297002	131012	0.46
9	136681035	136723533	42498	0.40
9	136739095	136740232	1137	0.06
9	136754269	136860923	106654	0.29
9	136936335	136984718	48383	0.23
9	137156924	137294126	137202	0.29
9	137403232	137467395	64163	0.40
10	555091	599976	44885	0.11
10	772409	792239	19830	0.40
10	940944	944216	3272	0.06
10	954440	972645	18205	0.06
10	2122824	2131168	8344	0.06
10	2833600	2835590	1990	0.29
10	2958497	2961645	3148	0.17
10	3728688	3730738	2050	0.06
10	4411120	4417857	6737	0.06
10	5371671	5377657	5986	0.17
10	5861874	5863298	1424	0.11
10	6166502	6173294	6792	0.06
10	6392942	6400186	7244	0.06
10	6647709	6651049	3340	0.06
10	7625841	7627589	1748	0.11
10	8268018	8280868	12850	0.23

10	9378393	9539963	161570	0.06
10	10614388	10618875	4487	0.40
10	12299476	12312071	12595	0.11
10	12578764	12592838	14074	0.06
10	12996207	13005437	9230	0.06
10	13070869	13086941	16072	0.06
10	14443206	14446968	3762	0.06
10	14946306	15021039	74733	0.06
10	15045474	15055617	10143	0.11
10	15410760	15413884	3124	0.06
10	16326089	16329769	3680	0.06
10	18964709	18969220	4511	0.06
10	19061551	19072671	11120	0.06
10	19134993	19139714	4721	0.06
10	19205064	19207887	2823	0.11
10	19465954	19474558	8604	0.06
10	20543783	20555017	11234	0.06
10	22903481	22911565	8084	0.06
10	24315764	24317924	2160	0.06
10	25867799	25869661	1862	0.11
10	26395163	26402038	6875	2.47
10	27318130	27418262	100132	0.92
10	29819631	29823042	3411	0.06
10	30253136	30342382	89246	0.63
10	30704185	30706090	1905	0.06
10	31218512	31255800	37288	0.29
10	31683454	31688402	4948	0.06
10	31880495	31901768	21273	0.06
10	33129089	33132620	3531	0.06
10	33719528	33732937	13409	0.06
10	33909083	33915175	6092	0.86
10	34961141	34967251	6110	0.06
10	35184557	35189199	4642	0.06
10	35887518	35888707	1189	0.06
10	36648634	36653166	4532	0.34
10	42876813	42879460	2647	0.29
10	43040089	43050719	10630	0.29
10	44915523	44917339	1816	0.29
10	45417323	45425396	8073	0.69
10	47348233	47357676	9443	0.52
10	49797741	49818810	21069	0.06
10	51512034	51535254	23220	0.17
10	51667587	51835743	168156	0.06
10	52784801	52790014	5213	0.63
10	52804603	52809080	4477	0.23
10	53167918	53177475	9557	1.61

10	53614714	53697548	82834	0.52
10	54325198	54327724	2526	0.06
10	54672482	54678648	6166	0.11
10	54760682	54782480	21798	0.57
10	55099774	55361364	261590	1.89
10	56775534	56786401	10867	0.06
10	57153439	57177179	23740	0.06
10	57415380	57486056	70676	0.46
10	58327865	58334514	6649	0.06
10	58868392	58871775	3383	0.06
10	60644471	60686460	41989	0.11
10	61407115	61408730	1615	0.11
10	61872165	61901581	29416	0.06
10	62234146	62247845	13699	0.06
10	64488054	64605163	117109	0.29
10	65281992	65290053	8061	0.06
10	65501436	65561243	59807	0.11
10	65567286	65584273	16987	0.29
10	66226903	66239347	12444	0.06
10	66315274	66354723	39449	0.23
10	66587307	66614538	27231	0.29
10	66731037	66779762	48725	0.06
10	67782114	67790109	7995	0.06
10	67854694	67867031	12337	0.06
10	68655758	68814077	158319	0.34
10	69579777	69587520	7743	0.06
10	70291636	70305220	13584	0.06
10	70977472	70983556	6084	0.11
10	71691619	71705276	13657	0.34
10	71727260	71738658	11398	0.17
10	72441913	72466597	24684	0.06
10	72561296	72712397	151101	0.17
10	72971840	72988124	16284	0.06
10	74048555	74057306	8751	0.06
10	74997649	75002230	4581	0.06
10	77573606	77581976	8370	0.06
10	78560093	78562342	2249	0.06
10	79146974	79160613	13639	0.23
10	81425343	81429197	3854	0.11
10	83866801	83869011	2210	0.11
10	84127298	84129173	1875	0.06
10	84981663	84987932	6269	0.11
10	85396496	85402491	5995	0.06
10	86191929	86199806	7877	0.34
10	86570177	86576522	6345	0.17
10	86653055	86663766	10711	1.03

10	86690301	86709830	19529	0.17
10	88643669	88646694	3025	0.06
10	90237985	90242355	4370	1.89
10	90556058	90588307	32249	0.06
10	90632615	90636363	3748	0.34
10	92897184	92999191	102007	0.23
10	93276818	93281804	4986	0.17
10	93923695	93935118	11423	0.06
10	93939246	93946277	7031	0.11
10	94408644	94535408	126764	0.17
10	94552250	94589459	37209	0.06
10	94820579	94839728	19149	0.29
10	95666329	95679583	13254	0.11
10	96396435	96404312	7877	0.11
10	98017502	98021308	3806	0.11
10	98819421	98826879	7458	0.06
10	98944840	99151606	206766	0.23
10	101966097	101973867	7770	0.06
10	102064329	102072932	8603	0.06
10	103244899	103253390	8491	0.06
10	103956782	103960346	3564	0.57
10	105013239	105017323	4084	0.06
10	106224562	106257058	32496	0.23
10	107423589	107431701	8112	0.06
10	108499676	108504409	4733	0.11
10	108543367	108557547	14180	2.75
10	108673885	108791347	117462	0.06
10	109526451	109642134	115683	0.29
10	111146915	111149811	2896	0.06
10	112375255	112380138	4883	0.06
10	112591242	112616199	24957	0.06
10	113334636	113339164	4528	0.11
10	113449709	113456619	6910	0.29
10	113560739	113564855	4116	0.06
10	114284828	114288999	4171	0.06
10	114728886	114730510	1624	0.17
10	117259666	117261896	2230	0.06
10	120838827	120841959	3132	0.06
10	122027395	122073742	46347	0.06
10	122563296	122570194	6898	0.11
10	123460305	123465648	5343	0.23
10	124705138	124706575	1437	0.06
10	124886641	124887889	1248	0.06
10	127290113	127292384	2271	0.06
10	127786137	127792810	6673	0.11
10	127854190	127856744	2554	0.06

10	128433593	128468423	34830	0.40
10	128497770	128499803	2033	0.11
10	128860638	128863076	2438	0.06
10	128886520	128889231	2711	0.57
10	130625651	130630920	5269	0.06
10	131284394	131286729	2335	0.06
10	131811600	131834476	22876	0.80
10	132399071	132404671	5600	0.06
10	132952715	132964785	12070	0.06
11	1021268	1039867	18599	0.86
11	1398381	1473478	75097	0.40
11	1691875	1695519	3644	0.11
11	1828103	1839194	11091	0.29
11	1916508	1938477	21969	0.46
11	1956322	1992578	36256	0.40
11	2011460	2052683	41223	0.40
11	2572418	2598160	25742	0.34
11	3721816	3758858	37042	0.06
11	4529499	4613512	84013	0.06
11	5044857	5051871	7014	0.17
11	5166403	5242219	75816	0.11
11	5844060	5869835	25775	8.95
11	6029735	6033321	3586	0.11
11	6167854	6172535	4681	0.46
11	7664187	7667798	3611	0.06
11	9929710	9952203	22493	0.06
11	10204325	10208304	3979	0.17
11	10788216	10792475	4259	0.06
11	11280317	11283961	3644	0.29
11	11688989	11691497	2508	0.06
11	12001351	12019425	18074	0.06
11	12240209	12253318	13109	0.11
11	15787749	15795202	7453	0.52
11	16615915	16620664	4749	0.06
11	17040907	17067458	26551	0.06
11	17189928	17211641	21713	0.06
11	17276116	17319323	43207	0.52
11	17465190	17469839	4649	0.34
11	17473504	17476247	2743	0.06
11	17574766	17579672	4906	0.06
11	18418191	18439631	21440	0.06
11	18587464	18598500	11036	0.11
11	19470467	19473068	2601	0.11
11	19516616	19519240	2624	0.06
11	20168561	20172250	3689	0.06
11	21401554	21403830	2276	0.34

11	21589423	21689672	100249	0.11
11	21926854	21934370	7516	0.06
11	24756913	24772778	15865	0.17
11	25101610	25274527	172917	0.34
11	25564364	25909715	345351	6.71
11	26141695	26223246	81551	0.11
11	26445513	26449563	4050	0.06
11	29885611	29888267	2656	0.06
11	31261830	31328261	66431	0.06
11	32669110	32724730	55620	0.06
11	33274045	33305645	31600	0.06
11	33361672	33371690	10018	0.06
11	33442721	33448334	5613	0.17
11	35762736	35769162	6426	0.06
11	37384393	37444223	59830	0.06
11	38434461	38448427	13966	0.11
11	38692233	38699680	7447	0.11
11	38953954	38962239	8285	0.06
11	39823594	39829738	6144	0.06
11	40166146	40167681	1535	0.06
11	40434450	40673841	239391	0.06
11	40931136	40947628	16492	0.17
11	40950364	40963951	13587	0.06
11	41117280	41119257	1977	0.06
11	42651474	42658769	7295	0.06
11	44064106	44085637	21531	0.06
11	45434750	45463891	29141	0.06
11	47625686	47756344	130658	0.29
11	47864812	47869782	4970	0.06
11	47873486	47903136	29650	0.06
11	49066286	49082234	15948	0.06
11	50135103	50162831	27728	0.11
11	57876022	57882668	6646	0.06
11	58691939	58704038	12099	0.06
11	59322956	59416719	93763	0.11
11	60135814	60216381	80567	0.06
11	62032773	62039584	6811	0.06
11	63015678	63018963	3285	0.06
11	63043086	63052586	9500	0.11
11	63095244	63104202	8958	0.06
11	63715719	63793381	77662	0.57
11	67269719	67307660	37941	0.06
11	68233112	68248727	15615	0.11
11	68463378	68580664	117286	0.06
11	69001614	69018191	16577	0.17
11	69189889	69197569	7680	0.06

11	74912966	74918138	5172	0.06
11	75545077	75549603	4526	0.06
11	76256797	76258705	1908	0.23
11	76659111	76668862	9751	0.23
11	76705616	76710987	5371	0.40
11	76764986	76797895	32909	0.46
11	77084793	77129979	45186	0.40
11	77177028	77208958	31930	1.26
11	78563123	78569277	6154	0.06
11	78647378	78656369	8991	0.06
11	79470559	79472758	2199	0.06
11	81272336	81306084	33748	0.52
11	82141468	82365345	223877	0.46
11	83109241	83112694	3453	0.06
11	83821792	83826348	4556	0.23
11	83849843	83862732	12889	0.06
11	84829533	84859671	30138	0.11
11	86859657	86862268	2611	0.23
11	87196551	87288398	91847	0.06
11	87500295	87509297	9002	0.06
11	87675905	87743887	67982	2.35
11	90264804	90279378	14574	0.06
11	90639468	90642337	2869	0.17
11	90849053	90856784	7731	0.17
11	91104348	91110121	5773	0.06
11	91350940	91360177	9237	0.06
11	91471433	91473860	2427	0.06
11	91542597	91548007	5410	0.06
11	92374030	92380283	6253	0.06
11	93780364	93789452	9088	0.17
11	95030873	95052339	21466	0.06
11	95129413	95132889	3476	0.06
11	95442698	95447515	4817	0.11
11	96246170	96247903	1733	0.06
11	96647886	96652386	4500	0.06
11	97103708	97177557	73849	0.23
11	97622333	97623612	1279	0.06
11	99215531	99225748	10217	0.06
11	99650260	99698597	48337	0.06
11	100263076	100284669	21593	0.06
11	100565963	100573792	7829	0.06
11	100912798	100916480	3682	0.17
11	101970244	102022191	51947	0.06
11	102630934	102635854	4920	0.29
11	103284025	103287549	3524	0.06
11	105053115	105060146	7031	4.19

11	105074180	105100635	26455	0.06
11	105179726	105196983	17257	0.29
11	106523477	106533881	10404	0.29
11	114297215	114303113	5898	0.17
11	114533054	114734835	201781	0.63
11	117013903	117036397	22494	0.06
11	117437716	117443604	5888	0.11
11	121999668	122030128	30460	1.26
11	124829565	124836588	7023	0.06
11	125204606	125213355	8749	0.23
11	125946544	125953802	7258	0.06
11	126963459	126979625	16166	0.06
11	128155371	128157904	2533	0.11
11	130462844	130468110	5266	0.06
11	131332741	131334995	2254	0.06
11	134282136	134344486	62350	0.11
12	1825242	1828635	3393	0.06
12	2136728	2150500	13772	1.43
12	3739945	3751056	11111	0.06
12	4820934	4846304	25370	0.11
12	5133635	5137125	3490	0.06
12	5957143	6017761	60618	0.06
12	6216522	6218947	2425	0.17
12	6249028	6254134	5106	0.06
12	7565506	7568485	2979	0.57
12	7662594	7725063	62469	0.46
12	7728764	7806996	78232	0.29
12	8738931	8745367	6436	0.06
12	8858075	8861159	3084	0.17
12	9806553	9808919	2366	0.11
12	9825309	9833901	8592	0.06
12	9879456	9881871	2415	0.06
12	12297598	12301714	4116	0.06
12	13483242	13492480	9238	0.06
12	13721333	13724496	3163	0.06
12	13933200	13937502	4302	0.06
12	14431165	14454787	23622	0.11
12	15415250	15421148	5898	1.09
12	17230728	17233424	2696	0.06
12	18232372	18239955	7583	0.40
12	19762074	19770824	8750	0.11
12	20864745	20881992	17247	0.11
12	21355623	21366821	11198	0.06
12	21783449	21798119	14670	0.06
12	21978568	21987430	8862	0.06
12	21996011	21997869	1858	0.06

12	22461495	22484740	23245	0.17
12	22714010	22718726	4716	0.17
12	23001521	23004537	3016	0.06
12	25495742	25501520	5778	0.06
12	25982029	25986589	4560	0.23
12	28858243	28862986	4743	0.46
12	29663372	29667982	4610	0.06
12	30960466	30961636	1170	0.40
12	31352080	31358268	6188	0.11
12	31382587	31383606	1019	0.46
12	31747816	31761490	13674	0.06
12	31899488	31923920	24432	0.57
12	32382881	32432643	49762	0.06
12	39425100	39431811	6711	0.06
12	40213764	40218182	4418	0.06
12	40426199	40427374	1175	0.11
12	40789198	40791489	2291	0.06
12	41102837	41121790	18953	0.11
12	43535318	43541196	5878	0.06
12	45104610	45131928	27318	0.06
12	48326356	48345229	18873	0.06
12	49339765	49348866	9101	0.06
12	49522157	49526612	4455	0.06
12	49544030	49563158	19128	0.06
12	50942186	50950271	8085	0.06
12	52000827	52010834	10007	0.06
12	52372779	52378665	5886	0.06
12	54480686	54492905	12219	0.29
12	54596339	54602111	5772	0.11
12	54909029	54917297	8268	0.06
12	55087817	55096074	8257	0.06
12	55514501	55530240	15739	0.75
12	56514114	56583666	69552	0.29
12	56748008	56764726	16718	0.06
12	59114606	59121938	7332	0.23
12	59526601	59554360	27759	0.17
12	60498484	60506092	7608	0.06
12	60796566	60808400	11834	0.06
12	61015330	61017772	2442	0.06
12	61867629	61872072	4443	0.06
12	63147944	63151375	3431	0.06
12	64271515	64384638	113123	0.29
12	65269412	65277255	7843	0.06
12	66722054	66725924	3870	0.06
12	68417768	68424871	7103	0.06
12	70858350	70863557	5207	0.06

12	72359410	72369780	10370	0.06
12	72431108	72444161	13053	0.06
12	73401240	73424800	23560	0.17
12	73597862	73601707	3845	0.06
12	73688563	73715158	26595	0.17
12	76100309	76136590	36281	1.43
12	76706241	76713558	7317	0.06
12	77660330	77674320	13990	0.11
12	79460078	79482137	22059	0.11
12	79794032	79813732	19700	0.06
12	84014095	84033677	19582	0.06
12	84312697	84327813	15116	0.11
12	84951695	84961159	9464	0.11
12	86771724	86879880	108156	0.06
12	87018320	87031385	13065	0.11
12	88311426	88332765	21339	0.06
12	90552916	90763783	210867	0.11
12	92308478	92313029	4551	0.06
12	93255121	93271141	16020	0.23
12	93315763	93317252	1489	0.23
12	93484592	93503072	18480	0.29
12	93803903	93811430	7527	0.46
12	94478816	94488668	9852	0.06
12	95135622	95171113	35491	0.86
12	95362692	95389711	27019	0.11
12	96039562	96041461	1899	0.06
12	96440374	96457824	17450	0.06
12	97361236	97375350	14114	0.06
12	98193661	98209114	15453	0.23
12	101410671	101472396	61725	0.52
12	102628334	102631121	2787	0.06
12	102866082	102869482	3400	0.06
12	103802227	103808913	6686	0.06
12	107568194	107574299	6105	0.11
12	108173419	108175692	2273	0.11
12	110604531	110610242	5711	0.57
12	110906526	110915044	8518	0.06
12	111806340	111812396	6056	0.06
12	113359066	113371457	12391	0.06
12	114044311	114050990	6679	0.06
12	114635794	114644389	8595	0.06
12	117922745	117931579	8834	0.06
12	118118818	118132502	13684	0.06
12	119552240	119553972	1732	0.06
12	121180338	121187288	6950	0.06
12	121787389	121805999	18610	0.06

12	122007327	122019673	12346	0.06
12	122105918	122123662	17744	0.06
12	122557457	122600613	43156	0.11
12	122844892	122870494	25602	0.52
12	123088247	123091734	3487	0.06
12	124256444	124264337	7893	0.06
12	124291951	124297598	5647	0.06
12	126338247	126343654	5407	0.11
12	126921858	126924576	2718	0.06
12	127005346	127010817	5471	0.06
12	127107838	127118110	10272	0.40
12	127796998	127805097	8099	0.06
12	128072414	128093983	21569	1.09
12	129043684	129055401	11717	0.17
12	129272084	129274511	2427	0.06
12	129811102	129813274	2172	0.23
12	129821758	129840428	18670	0.86
12	130007765	130010595	2830	0.11
12	130453014	130460912	7898	0.06
12	130733140	130735876	2736	0.11
12	131245772	131340814	95042	1.09
12	131370928	131380721	9793	0.06
12	131484705	131486583	1878	0.11
12	131540628	131543469	2841	0.06
12	131577718	131631122	53404	0.06
12	132393473	132410877	17404	0.11
12	132430921	132434067	3146	0.11
12	132632393	132649610	17217	0.06
13	20228784	20460311	231527	0.23
13	20914428	20923730	9302	0.06
13	22255776	22261708	5932	0.06
13	22329412	22339188	9776	0.06
13	23039738	23064163	24425	0.46
13	23413970	23422058	8088	0.06
13	24738385	24741868	3483	0.06
13	24995860	25005198	9338	0.86
13	26471742	26475476	3734	0.06
13	26912056	26915221	3165	0.06
13	26948828	26964359	15531	0.06
13	27061300	27066217	4917	0.06
13	27992958	28009029	16071	0.34
13	28218588	28263159	44571	0.06
13	30075614	30082996	7382	0.11
13	30227128	30232241	5113	0.06
13	30947166	30952348	5182	0.06
13	31638987	31645249	6262	0.06

13	32097968	32099700	1732	1.03
13	32116838	32118173	1335	0.06
13	32757748	32777770	20022	0.17
13	36919839	36937654	17815	0.06
13	37496412	37546138	49726	0.97
13	37751408	37763530	12122	0.06
13	37853082	37855647	2565	0.17
13	38918942	38923532	4590	0.06
13	39545273	39550746	5473	0.57
13	39858567	39864762	6195	0.17
13	39887317	39890802	3485	0.06
13	40527751	40540636	12885	0.06
13	42068410	42072179	3769	0.06
13	42726161	42728389	2228	0.06
13	47173385	47187182	13797	0.06
13	48356448	48368678	12230	0.11
13	48918949	48930674	11725	0.06
13	49376947	49407144	30197	0.34
13	50598827	50606287	7460	0.11
13	51534431	51536485	2054	0.06
13	52578892	52599452	20560	0.29
13	54213723	54247776	34053	0.23
13	54502823	54513664	10841	0.06
13	55761580	55821875	60295	0.11
13	56413072	56428550	15478	0.06
13	57239433	57387355	147922	2.06
13	60533179	60567284	34105	0.06
13	60636327	60650236	13909	0.86
13	62017732	62026243	8511	0.29
13	62374856	62378736	3880	0.06
13	76036154	76039093	2939	1.32
13	76690994	76697218	6224	0.06
13	77360500	77362644	2144	0.06
13	78519634	78549041	29407	0.06
13	81674842	81709378	34536	0.06
13	81842320	81857711	15391	0.11
13	81925430	81938183	12753	0.06
13	82409359	82427027	17668	0.06
13	83527345	83582139	54794	1.66
13	83615544	83619236	3692	0.06
13	84034948	84050777	15829	0.06
13	84790917	84795232	4315	0.06
13	85285616	85308725	23109	1.38
13	85336739	85338172	1433	0.06
13	86684237	86934184	249947	7.28
13	86314889	86338351	23462	0.17

13	86946854	86953207	6353	0.06
13	87294220	87301793	7573	0.06
13	89759222	89767502	8280	0.11
13	91302190	91304927	2737	0.06
13	91842792	91846936	4144	0.06
13	93272492	93305563	33071	0.11
13	93672343	93678615	6272	0.06
13	95351476	95372983	21507	0.75
13	96268166	96274136	5970	1.03
13	96305871	96309138	3267	0.17
13	98262439	98275214	12775	0.06
13	98678914	98681429	2515	0.06
13	99578605	99580570	1965	0.11
13	99633681	99647305	13624	0.17
13	99829113	99832682	3569	0.11
13	101528387	101671867	143480	0.17
13	103972302	103978606	6304	0.06
13	104021503	104030691	9188	0.17
13	104144594	104161520	16926	0.06
13	104740970	104752416	11446	0.23
13	105580652	105653286	72634	0.29
13	107516183	107532961	16778	0.06
13	110089431	110110313	20882	0.06
13	111435289	111438181	2892	0.06
13	111937985	111944644	6659	0.29
13	111979418	111986621	7203	0.11
13	112181730	112184670	2940	0.06
13	112201459	112203478	2019	0.06
13	112880068	112893719	13651	0.06
13	112979265	113013506	34241	0.23
13	113396307	113399947	3640	0.23
14	20215935	20275110	59175	1.15
14	20836761	20875328	38567	0.06
14	20877548	20952929	75381	4.64
14	20972304	20978068	5764	0.11
14	21601552	21633789	32237	0.06
14	22612317	22614001	1684	0.11
14	22708063	22720040	11977	0.06
14	22789297	22791483	2186	0.06
14	22796599	22802985	6386	0.06
14	23384946	23413975	29029	0.06
14	23817031	23819293	2262	0.11
14	23978239	23988910	10671	0.11
14	24757227	24761450	4223	0.06
14	25793937	25796620	2683	0.06
14	26186477	26222799	36322	0.11

14	26723031	26728626	5595	0.06
14	27101354	27473006	371652	0.06
14	27936620	27965310	28690	0.06
14	27991880	28050598	58718	0.11
14	28105513	28483881	378368	1.20
14	30657532	30678129	20597	0.06
14	30804471	30811759	7288	0.06
14	31253232	31261966	8734	0.06
14	32706616	32712150	5534	0.17
14	32857325	32928169	70844	0.11
14	32969302	32973053	3751	0.11
14	33397214	33404032	6818	0.06
14	34547992	34626739	78747	0.52
14	36204669	36209380	4711	0.06
14	36658048	36664141	6093	0.06
14	37126427	37132737	6310	0.17
14	39843096	39848889	5793	0.06
14	39955901	40050209	94308	0.17
14	40148816	40173342	24526	0.06
14	41130816	41138799	7983	0.06
14	41274991	41501068	226077	0.29
14	41922415	41939740	17325	0.06
14	42363186	42370418	7232	0.06
14	42594675	42610488	15813	0.11
14	43019672	43034338	14666	0.06
14	43227128	43241157	14029	0.06
14	43376762	43412447	35685	0.06
14	43490010	43511500	21490	0.06
14	43561379	43619224	57845	0.06
14	44034845	44062214	27369	0.52
14	44307009	44328991	21982	0.06
14	44677278	44682347	5069	0.46
14	44712092	44754254	42162	0.29
14	47760853	47808269	47416	0.34
14	48320260	48443440	123180	0.06
14	48613609	48665931	52322	0.11
14	48722837	48784014	61177	0.06
14	48911691	48915645	3954	0.69
14	48926670	48944788	18118	0.23
14	52635966	52687018	51052	0.29
14	52771121	52777919	6798	0.06
14	53272117	53296289	24172	0.17
14	56379958	56433324	53366	0.11
14	56920314	56937236	16922	0.06
14	57666832	57675265	8433	0.69
14	59002955	59009283	6328	0.06

14	59198568	59201930	3362	0.06
14	59959510	60032791	73281	0.06
14	61497351	61516910	19559	0.17
14	61931149	61942464	11315	0.06
14	62331767	62334509	2742	0.23
14	62490173	62492818	2645	0.11
14	63111303	63116391	5088	0.06
14	63432267	63447059	14792	0.06
14	63489794	63496191	6397	0.29
14	63763371	63841725	78354	0.17
14	64009152	64032207	23055	0.34
14	65157091	65166076	8985	0.06
14	65174526	65176629	2103	0.06
14	65188381	65192018	3637	0.06
14	65862173	65868884	6711	0.06
14	69886900	69889517	2617	0.06
14	72402082	72409454	7372	0.06
14	73559982	73584938	24956	0.29
14	73769024	73778904	9880	0.11
14	73993924	74001940	8016	0.29
14	74027734	74074781	47047	0.06
14	74640832	74649233	8401	0.23
14	77145768	77147515	1747	0.06
14	77475120	77492472	17352	0.23
14	78068563	78079500	10937	0.06
14	78993068	79017240	24172	0.06
14	79539319	79547291	7972	0.06
14	82265862	82275144	9282	0.06
14	83060926	83437932	377006	0.06
14	83702151	83706040	3889	0.06
14	84615147	84628426	13279	0.06
14	85404493	85407192	2699	0.06
14	85994571	86022737	28166	0.80
14	89309840	89315495	5655	0.06
14	89462240	89470286	8046	0.06
14	90058540	90071571	13031	0.06
14	90148442	90151050	2608	0.11
14	92115481	92117685	2204	0.06
14	94041896	94045763	3867	0.17
14	94945834	94953775	7941	2.12
14	96061192	96068116	6924	0.11
14	96129215	96131580	2365	0.11
14	96889208	96897139	7931	0.06
14	97175041	97180740	5699	0.06
14	97727875	97743393	15518	0.11
14	98568439	98590755	22316	0.40

14	99107349	99112788	5439	0.06
14	100724670	100740386	15716	0.06
14	102551958	102568207	16249	0.06
14	104570952	104589639	18687	0.23
14	104769950	104804187	34237	0.06
14	104852515	104898819	46304	0.29
14	105383761	105397519	13758	0.69
15	24112065	24518827	406762	2.52
15	24821213	24839447	18234	0.29
15	24845258	24871394	26136	0.11
15	24923986	24927476	3490	0.11
15	26505921	26518060	12139	0.06
15	31745235	31752985	7750	0.06
15	32216889	32327926	111037	2.24
15	33226279	33227964	1685	0.06
15	35057529	35059024	1495	0.06
15	36155410	36162641	7231	0.06
15	37924977	37928190	3213	0.06
15	38953515	38971288	17773	0.17
15	40636415	40667426	31011	0.11
15	40706144	40716144	10000	0.06
15	41160937	41200815	39878	0.17
15	41924350	41930192	5842	0.06
15	42070825	42083765	12940	0.11
15	42098025	42099966	1941	0.17
15	42112316	42115568	3252	0.86
15	42525828	42588152	62324	0.17
15	43023851	43032351	8500	0.06
15	44946097	44989584	43487	0.52
15	45109926	45117706	7780	0.17
15	46151495	46161683	10188	0.11
15	49520918	49523178	2260	0.06
15	50256899	50272687	15788	0.06
15	50591947	50748601	156654	0.52
15	51195155	51199657	4502	0.06
15	51966500	52004439	37939	0.46
15	52090724	52095803	5079	0.06
15	52716726	52718586	1860	0.06
15	52782295	52790685	8390	0.06
15	55415501	55438983	23482	0.06
15	55801540	55810338	8798	0.06
15	57710991	57713313	2322	0.06
15	58199610	58222367	22757	0.06
15	58429614	58439256	9642	0.06
15	58508567	58574881	66314	0.06
15	59308916	59312337	3421	0.06

15	59759843	59803362	43519	0.69
15	66735166	66742613	7447	0.06
15	66764233	66766921	2688	0.06
15	67984005	68004550	20545	0.06
15	68459549	68464362	4813	0.17
15	68743881	68768599	24718	0.17
15	69371049	69375434	4385	0.06
15	70194233	70197005	2772	0.06
15	72414443	72432875	18432	0.06
15	73085681	73138420	52739	0.06
15	74253301	74259262	5961	0.29
15	74295847	74318059	22212	0.23
15	74335666	74338282	2616	0.06
15	75489213	75522363	33150	0.23
15	75670873	75701407	30534	0.11
15	76201024	76213679	12655	0.06
15	76646259	76679115	32856	0.06
15	76995740	77034495	38755	0.34
15	77256301	77281885	25584	0.06
15	77544008	77592162	48154	0.46
15	77606077	77620087	14010	0.06
15	77780520	77799190	18670	0.69
15	79256109	79261492	5383	0.86
15	80434281	80436028	1747	0.06
15	82104669	82109185	4516	0.80
15	82634768	82642084	7316	0.06
15	84917702	84958185	40483	0.17
15	85786279	85789950	3671	0.06
15	85814285	85820469	6184	0.46
15	85839982	85843332	3350	0.06
15	86428401	86473279	44878	0.23
15	86951933	86960021	8088	0.06
15	87188292	87194642	6350	0.17
15	87362985	87366884	3899	0.75
15	87603315	87627650	24335	0.06
15	89066719	89070819	4100	0.17
15	89631201	89645161	13960	0.06
15	89655941	89664988	9047	0.06
15	89675931	89731525	55594	0.06
15	89822228	89830207	7979	0.06
15	89924262	89971157	46895	0.06
15	90308068	90310105	2037	0.06
15	92429951	92651958	222007	0.23
15	93253947	93255540	1593	0.06
15	93313961	93323262	9301	0.34
15	93572837	93580568	7731	0.11

15	95447283	95451555	4272	0.06
15	96719197	96725488	6291	0.11
15	98132833	98136845	4012	0.11
15	98236794	98240955	4161	0.34
15	99065787	99084185	18398	0.11
15	99097113	99099267	2154	0.34
15	100656059	100661399	5340	0.34
15	101409722	101419713	9991	0.06
16	1729088	1778838	49750	0.34
16	1833278	1919876	86598	0.92
16	1964282	1985921	21639	0.06
16	1997862	2004154	6292	0.17
16	2058754	2109241	50487	0.63
16	2272715	2299205	26490	0.06
16	2357438	2404509	47071	0.29
16	2915518	2978416	62898	0.06
16	3524622	3528630	4008	0.06
16	3553179	3576300	23121	0.80
16	3674351	3676093	1742	0.06
16	4058741	4093180	34439	0.23
16	4173961	4179383	5422	0.06
16	4891046	4902458	11412	0.40
16	4979496	4996207	16711	0.06
16	6042692	6051067	8375	0.06
16	6097580	6099243	1663	0.06
16	6556677	6639465	82788	0.11
16	6749201	6751845	2644	0.06
16	6793114	6803745	10631	0.11
16	6885886	6982360	96474	0.23
16	6989597	7078136	88539	0.11
16	8071351	8074842	3491	0.75
16	8418992	8425901	6909	0.06
16	9024742	9045221	20479	0.23
16	9165375	9170501	5126	0.06
16	10555182	10558906	3724	0.06
16	10634387	10635681	1294	0.34
16	10699285	10711898	12613	0.46
16	11154606	11160470	5864	0.06
16	11192572	11197270	4698	0.06
16	11604850	11622878	18028	0.06
16	12558104	12560975	2871	0.17
16	12627664	12631355	3691	0.06
16	13061016	13062909	1893	0.06
16	13274082	13284432	10350	0.06
16	13568822	13578087	9265	0.11
16	14558982	14567379	8397	0.06

16	19974855	19979291	4436	0.06
16	22379935	22428998	49063	0.06
16	22616132	22692420	76288	0.23
16	24404303	24406215	1912	0.06
16	24437735	24452238	14503	0.06
16	25186259	25190659	4400	0.06
16	25619297	25627932	8635	0.11
16	25745922	25759427	13505	0.17
16	27325106	27339366	14260	0.17
16	28591670	28618832	27162	1.38
16	30377388	30385962	8574	0.06
16	31408522	31424558	16036	0.06
16	31701124	31708731	7607	0.11
16	32457714	32638415	180701	5.10
16	34025437	34117342	91905	3.04
16	34121562	34130550	8988	0.06
16	47882325	47894006	11681	0.57
16	48424421	48427842	3421	0.06
16	49703131	49706034	2903	0.11
16	49922283	49926346	4063	0.06
16	52339798	52344524	4726	0.11
16	55193022	55197284	4262	0.06
16	58336933	58349751	12818	0.11
16	58638361	58649955	11594	0.06
16	58983958	58991483	7525	0.40
16	59213779	59220586	6807	0.06
16	60048702	60064640	15938	0.80
16	60261060	60429506	168446	0.06
16	64041792	64080394	38602	0.97
16	64230338	64319776	89438	0.06
16	64879394	64892279	12885	0.06
16	66262541	66265815	3274	0.06
16	67184339	67203991	19652	0.06
16	68711938	68743930	31992	0.06
16	69109129	69115407	6278	0.06
16	69618542	69671023	52481	0.23
16	69690320	69706471	16151	0.23
16	69818655	69821723	3068	0.06
16	71949758	71981464	31706	0.06
16	73586806	73590919	4113	0.06
16	75497871	75544561	46690	0.29
16	75959815	76022476	62661	0.11
16	76392323	76393644	1321	0.06
16	76630598	76637010	6412	2.41
16	77099041	77101870	2829	0.06
16	77190683	77195253	4570	0.06

16	77713212	77720677	7465	0.06
16	78008733	78044242	35509	0.06
16	78117440	78138201	20761	0.17
16	78297437	78301601	4164	0.06
16	78351415	78369728	18313	0.06
16	78467048	78475182	8134	0.06
16	78910158	78914180	4022	0.40
16	79226868	79239785	12917	0.40
16	80092692	80102545	9853	0.06
16	81472988	81476122	3134	0.06
16	82804425	82816102	11677	0.06
16	82930026	82939716	9690	0.06
16	83162838	83175549	12711	0.34
16	83857418	83862148	4730	0.29
16	84014893	84018865	3972	0.06
16	84351810	84357821	6011	0.06
16	84389150	84399428	10278	0.11
16	84891388	84896955	5567	0.06
16	85011068	85024688	13620	0.11
16	85086894	85091106	4212	0.06
16	85868890	85872665	3775	0.11
16	85981541	85984109	2568	0.06
16	86077333	86086079	8746	0.06
16	86126873	86129615	2742	0.06
16	86734866	86739157	4291	0.06
16	87025260	87030270	5010	0.06
16	87799745	87812868	13123	0.11
16	88412559	88502518	89959	0.34
16	89085500	89258444	172944	0.52
16	89510358	89542941	32583	0.06
16	89582172	89590223	8051	0.23
16	89694244	89713682	19438	0.52
17	669230	676608	7378	0.06
17	863159	914836	51677	0.06
17	926526	946069	19543	0.06
17	1429808	1431357	1549	0.06
17	1444355	1449407	5052	0.06
17	1580292	1591366	11074	0.06
17	1727290	1754605	27315	0.23
17	1812141	1819019	6878	0.06
17	1824484	1837648	13164	0.06
17	2142081	2166389	24308	0.06
17	3451970	3462094	10124	0.06
17	3601380	3656529	55149	0.06
17	4377518	4398152	20634	0.06
17	4446804	4448703	1899	0.11

17	4533059	4552832	19773	0.52
17	6968559	6973541	4982	0.11
17	9334559	9348828	14269	0.06
17	10858182	10862167	3985	0.40
17	12441028	12449819	8791	0.06
17	16106601	16113495	6894	0.06
17	17497658	17543170	45512	0.17
17	17783027	17812355	29328	0.11
17	18118725	18123829	5104	0.06
17	18229963	18252087	22124	0.06
17	18972458	19005730	33272	0.17
17	19590814	19634157	43343	3.96
17	19738008	19742798	4790	0.52
17	20935101	20940244	5143	0.06
17	28420462	28435672	15210	0.06
17	28835436	28843138	7702	0.06
17	29423532	29489668	66136	0.80
17	30107814	30183232	75418	0.29
17	30759639	30904681	145042	0.92
17	31182394	31234697	52303	0.11
17	31507754	31513400	5646	0.11
17	31704788	31715630	10842	0.17
17	33660771	33668288	7517	0.11
17	35354316	35441265	86949	0.23
17	36085689	36153338	67649	0.17
17	36461875	37890178	1428303	0.75
17	38927724	38996971	69247	0.52
17	39243242	39252489	9247	0.06
17	41122053	41132726	10673	0.29
17	41256142	41264680	8538	0.06
17	44258576	44262855	4279	0.23
17	44404704	44493354	88650	0.11
17	44757761	44781608	23847	0.23
17	45835216	45851500	16284	0.29
17	46944098	46989499	45401	0.06
17	47061561	47075159	13598	0.40
17	47138377	47168960	30583	0.23
17	47800841	47808390	7549	0.11
17	47963029	47973269	10240	0.06
17	49634976	49650737	15761	0.06
17	50232082	50240967	8885	0.11
17	52867186	52873738	6552	0.06
17	53849964	53861555	11591	0.06
17	54827112	54831095	3983	0.06
17	58664311	58672267	7956	0.17
17	59277357	59312932	35575	0.06

17	60132228	60160277	28049	0.29
17	60444947	60516473	71526	0.29
17	60698206	60774498	76292	0.17
17	61970281	61994410	24129	0.06
17	62664556	62698051	33495	0.57
17	63045926	63085561	39635	0.06
17	63939683	63973168	33485	0.40
17	64638752	64652251	13499	0.06
17	69495791	69503576	7785	0.06
17	72792602	72798228	5626	0.40
17	72818994	72823658	4664	0.23
17	73750237	73752292	2055	1.38
17	74281740	74305569	23829	0.06
17	75077648	75082964	5316	0.11
17	75162278	75210556	48278	0.06
17	76132255	76138113	5858	0.11
17	76656192	76662002	5810	0.06
17	77270169	77274756	4587	0.34
17	77517625	77525013	7388	0.06
17	77635768	77663499	27731	0.06
17	78420744	78433469	12725	0.23
17	79083494	79096944	13450	0.40
17	80002336	80012331	9995	0.17
17	80824354	80832227	7873	0.06
17	81359903	81364197	4294	0.06
17	81409814	81462332	52518	0.46
17	81692041	81696850	4809	0.06
17	81711066	81720891	9825	0.17
17	82076922	82095290	18368	0.86
17	82291935	82333968	42033	0.06
18	508455	515876	7421	0.11
18	1198102	1204388	6286	0.06
18	1707172	1839338	132166	0.29
18	1905524	1982550	77026	0.34
18	2551773	2561264	9491	0.17
18	4155326	4163810	8484	0.17
18	4329893	4335067	5174	1.09
18	4467980	4478774	10794	0.06
18	5148002	5177967	29965	0.06
18	5926003	5934150	8147	1.26
18	6544261	6547132	2871	0.06
18	7304510	7307152	2642	0.06
18	7687293	7716252	28959	0.06
18	8453781	8455963	2182	0.06
18	8479545	8486158	6613	0.06
18	8734891	8736059	1168	0.23

18	8842882	8845225	2343	0.06
18	10135337	10143880	8543	0.17
18	10286881	10289167	2286	0.11
18	10913665	10920284	6619	0.06
18	11559235	11566111	6876	0.29
18	11682429	11685670	3241	0.46
18	13481721	13488357	6636	0.06
18	13796352	13804920	8568	0.06
18	13809357	13815681	6324	0.29
18	14549800	14569900	20100	0.23
18	14767226	14781600	14374	0.17
18	22463469	22483610	20141	0.80
18	26285904	26318807	32903	0.06
18	26959306	27095784	136478	0.06
18	30701259	30703445	2186	0.29
18	32019229	32056105	36876	0.06
18	32917884	32923371	5487	0.23
18	34144420	34161840	17420	0.06
18	35770922	35777714	6792	0.17
18	35886532	35896604	10072	0.06
18	36675153	36677628	2475	0.06
18	37297998	37307236	9238	0.23
18	37951854	37956410	4556	0.17
18	38846732	38913547	66815	0.06
18	39361646	39382632	20986	0.23
18	39893876	40074303	180427	0.11
18	43300764	43328621	27857	0.97
18	44185713	44196001	10288	0.11
18	47709030	47715734	6704	0.06
18	48785857	48788344	2487	0.06
18	48835749	48843170	7421	0.06
18	51299558	51302974	3416	0.06
18	51690001	51696873	6872	0.17
18	52697828	52741180	43352	0.06
18	53751725	54019362	267637	0.06
18	55899240	55906136	6896	0.06
18	56796689	56817415	20726	0.06
18	57751213	57756011	4798	0.06
18	58262733	58269877	7144	0.29
18	59128155	59135318	7163	0.29
18	59648900	60373354	724454	0.75
18	61436808	61438126	1318	0.06
18	62044077	62047523	3446	0.06
18	63695508	63698973	3465	0.06
18	63781955	63786305	4350	0.06
18	64388614	64394378	5764	0.06

18	65193942	65198125	4183	0.06
18	66165678	66202607	36929	0.46
18	66408511	66424228	15717	0.06
18	66633991	66649298	15307	0.06
18	67223103	67226988	3885	0.52
18	67629278	67651557	22279	3.78
18	67852277	67860216	7939	0.06
18	68928378	68955687	27309	0.06
18	69108747	69118448	9701	0.06
18	70609833	70617574	7741	0.06
18	70785891	70787771	1880	0.06
18	71681925	71685516	3591	0.46
18	71821634	71831310	9676	0.11
18	72108877	72114851	5974	0.06
18	72973403	72975319	1916	0.11
18	76753980	76758869	4889	0.06
18	77051138	77058717	7579	0.17
18	77531405	77539104	7699	0.06
18	77702180	77704253	2073	0.06
18	78409421	78410716	1295	0.23
18	78741662	78751454	9792	0.11
18	78924386	78927314	2928	0.06
18	79166524	79169090	2566	0.06
18	79574401	79591204	16803	0.63
19	624683	665186	40503	0.29
19	836557	841398	4841	0.06
19	1134574	1168989	34415	0.34
19	1456115	1508739	52624	0.11
19	1781709	1789961	8252	0.23
19	2110747	2136516	25769	0.17
19	2577529	2610951	33422	0.06
19	2895803	2926995	31192	0.06
19	3908041	3913476	5435	0.11
19	3930876	3951842	20966	0.11
19	4131135	4145774	14639	0.06
19	4358309	4368145	9836	0.06
19	5178268	5203845	25577	0.11
19	5455411	5456919	1508	0.23
19	5688177	5711919	23742	0.11
19	6957399	7074550	117151	0.75
19	7251090	7252833	1743	0.34
19	7422427	7426503	4076	0.06
19	7620424	7626579	6155	0.06
19	8499028	8523034	24006	0.23
19	11118289	11133332	15043	0.29
19	11173352	11174714	1362	0.40

19	11575133	11579208	4075	0.17
19	12039015	12059799	20784	0.17
19	12389335	12430683	41348	0.11
19	13323903	13331981	8078	0.06
19	13393890	13400225	6335	0.06
19	14021847	14034371	12524	0.06
19	14382490	14393213	10723	0.06
19	14802199	14806157	3958	0.06
19	15553842	15566538	12696	0.34
19	16005214	16026113	20899	0.23
19	16456956	16463965	7009	0.06
19	16957714	16980331	22617	0.06
19	18144549	18175736	31187	0.06
19	18429834	18476362	46528	0.06
19	18736055	18782426	46371	0.06
19	20050922	20054666	3744	0.29
19	20300734	20308406	7672	0.06
19	20517745	20774347	256602	5.16
19	20729406	20743945	14539	0.11
19	20874267	20911271	37004	0.23
19	20973591	20983030	9439	1.72
19	21208316	21217326	9010	0.06
19	21561810	21565585	3775	0.06
19	21566912	21574212	7300	0.06
19	21777605	21787532	9927	0.17
19	23190196	23247634	57438	0.17
19	29427670	29430164	2494	0.06
19	29491118	29508333	17215	0.11
19	30826297	30827451	1154	2.52
19	32553186	32558150	4964	0.06
19	33385488	33415858	30370	0.29
19	35393607	35396746	3139	0.06
19	36365349	36369320	3971	0.06
19	36459269	36472735	13466	0.11
19	36858363	36867409	9046	0.06
19	37539101	37541197	2096	0.06
19	39630318	39632896	2578	0.06
19	41144662	41163234	18572	0.11
19	41460735	41501029	40294	0.46
19	41514643	41527927	13284	0.06
19	41684301	41691199	6898	0.06
19	42739884	43344645	604761	4.82
19	44389361	44434513	45152	0.23
19	44454835	44458652	3817	0.57
19	46052953	46067397	14444	0.06
19	46213459	46233669	20210	0.06

19	47289928	47318881	28953	0.11
19	48055029	48079982	24953	0.34
19	48702748	48705753	3005	0.06
19	49869505	49883083	13578	0.06
19	50194630	50196835	2205	0.06
19	50357351	50367890	10539	0.06
19	50378562	50389547	10985	0.06
19	50397768	50417880	20112	0.17
19	50795609	50798809	3200	0.06
19	51232344	51236512	4168	0.06
19	51624491	51629139	4648	0.06
19	51723207	51729071	5864	0.06
19	52262690	52274366	11676	0.92
19	53061869	53066715	4846	0.06
19	53287985	53295692	7707	0.06
19	53429042	53511584	82542	0.11
19	53798030	53805300	7270	0.06
19	54129140	54133660	4520	0.11
19	54172738	54175471	2733	0.11
19	54481397	54662761	181364	0.06
19	54947433	54986475	39042	0.17
19	55224031	55231895	7864	0.11
19	55991992	55998854	6862	0.06
19	56062244	56071940	9696	0.06
19	56740087	56743612	3525	0.29
19	56961145	56969373	8228	0.46
19	58026679	58032424	5745	0.23
20	1696297	1698104	1807	0.06
20	2558741	2561780	3039	0.06
20	2901348	2928644	27296	0.11
20	3899481	3904453	4972	0.06
20	5507202	5513196	5994	0.06
20	5592311	5601811	9500	0.06
20	7097053	7107791	10738	0.17
20	9006598	9028516	21918	0.06
20	9712122	9729521	17399	0.29
20	12407561	12545151	137590	0.29
20	12961031	12970412	9381	0.17
20	14290118	14298581	8463	0.06
20	14438097	14477903	39806	0.57
20	14748976	14899681	150705	0.57
20	14903486	14936623	33137	0.06
20	15646095	15661936	15841	0.11
20	15770355	15783700	13345	0.17
20	16095377	16103137	7760	1.38
20	16682424	16685408	2984	0.34

20	17614825	17638275	23450	0.23
20	18269010	18272729	3719	0.11
20	19012470	19020843	8373	0.06
20	19169698	19182638	12940	0.23
20	22342087	22349259	7172	0.40
20	22362468	22369888	7420	0.06
20	22393676	22402402	8726	0.06
20	23187139	23194046	6907	1.84
20	24436524	24439555	3031	0.06
20	32297780	32327658	29878	0.06
20	32456388	32470559	14171	0.11
20	32929983	32932914	2931	0.06
20	33090954	33100524	9570	0.06
20	35269203	35278049	8846	0.06
20	37521471	37522702	1231	0.06
20	38252497	38259681	7184	0.06
20	40195143	40199901	4758	0.06
20	42430346	42432573	2227	0.06
20	42553671	42571017	17346	0.06
20	42621393	42649685	28292	0.80
20	43114342	43119491	5149	0.06
20	43565968	43572296	6328	0.23
20	45822913	45827251	4338	0.06
20	47431079	47451780	20701	0.11
20	48882019	48883538	1519	0.75
20	49991211	49994803	3592	0.06
20	50452721	50460356	7635	0.06
20	50662198	50672690	10492	0.06
20	50680041	50690397	10356	0.06
20	50751280	50775369	24089	0.17
20	51134734	51141135	6401	0.11
20	51859193	51895977	36784	0.29
20	51948629	52000847	52218	0.29
20	52206766	52210061	3295	0.06
20	53218826	53227195	8369	0.11
20	53472262	53478881	6619	0.06
20	53725130	53729867	4737	0.06
20	55453377	55463974	10597	0.06
20	57892036	57896547	4511	0.06
20	59721097	59738411	17314	0.29
20	60014013	60025147	11134	0.06
20	61068286	61069423	1137	0.29
20	61590949	61592121	1172	0.06
20	61881019	61882797	1778	0.06
20	63081166	63114187	33021	0.29
20	63374931	63391446	16515	0.11

20	63688889	63704214	15325	0.23
20	63789957	63819726	29769	0.34
21	14029377	14045474	16097	0.06
21	14291719	14294855	3136	0.06
21	14428641	14443573	14932	0.06
21	14703410	14705988	2578	0.06
21	16292355	16295458	3103	0.06
21	17671053	17674435	3382	0.17
21	21051010	21078185	27175	0.06
21	21177234	21182954	5720	0.06
21	21483538	21494766	11228	0.11
21	22804542	22833225	28683	0.75
21	22951909	23082655	130746	1.03
21	23120376	23267071	146695	0.23
21	23310475	23331997	21522	0.06
21	24123601	24134373	10772	0.06
21	24660054	24672091	12037	0.06
21	25088844	25095887	7043	0.06
21	26652378	26656503	4125	0.40
21	27006205	27034820	28615	0.11
21	27167631	27177342	9711	0.23
21	27464543	27472174	7631	0.11
21	27812806	27819704	6898	0.06
21	28754173	28755967	1794	0.11
21	29118852	29127576	8724	0.06
21	30529083	30693503	164420	0.06
21	30813490	30822200	8710	0.06
21	31314593	31318848	4255	0.06
21	35078544	35080052	1508	0.06
21	35188777	35193227	4450	0.29
21	36635495	36642922	7427	0.06
21	36779087	36785349	6262	0.17
21	37305994	37341883	35889	1.15
21	37737174	37742921	5747	0.63
21	38277627	38283071	5444	0.40
21	38539698	38544832	5134	0.17
21	38562277	38568966	6689	0.06
21	41425679	41428539	2860	0.06
21	41955702	41958976	3274	0.11
21	41987559	41990376	2817	0.11
21	42445480	42446949	1469	0.06
21	42849478	42854987	5509	0.06
21	43166998	43174596	7598	0.17
21	43281032	43297318	16286	0.06
21	43360714	43382137	21423	0.17
21	43397020	43404312	7292	0.17

21	43814270	43826895	12625	0.92
21	44210503	44211628	1125	0.06
21	44216504	44244374	27870	0.17
21	44391460	44414033	22573	1.20
21	44575059	44597773	22714	0.11
21	45265260	45266911	1651	0.06
21	45668360	45693763	25403	0.34
21	45975479	46011117	35638	1.03
22	18857119	19022636	165517	0.80
22	19709424	19743577	34153	0.69
22	19954180	19992777	38597	0.86
22	20595197	20613163	17966	0.17
22	20990595	21003313	12718	0.11
22	21089741	21099699	9958	0.06
22	21958756	22219245	260489	0.34
22	22362959	22380302	17343	0.06
22	23203247	23230332	27085	0.17
22	24831727	24832749	1022	0.06
22	25268027	25532662	264635	1.15
22	26398136	26405009	6873	0.11
22	26721727	26733889	12162	0.06
22	27259064	27261546	2482	0.11
22	29061594	29063646	2052	0.06
22	31494313	31511999	17686	0.06
22	31790018	31805785	15767	0.11
22	32543490	32547755	4265	0.06
22	33720552	33728990	8438	0.06
22	34451779	34455385	3606	0.06
22	34543323	34585637	42314	1.95
22	34711004	34719922	8918	0.40
22	35012904	35019789	6885	0.06
22	36045986	36095458	49472	0.06
22	36548793	36561259	12466	0.34
22	37127968	37132566	4598	0.11
22	37295577	37300514	4937	0.06
22	37583257	37588857	5600	0.34
22	37918171	37942015	23844	0.57
22	38281769	38287631	5862	0.06
22	38968338	38996475	28137	2.18
22	39375109	39383361	8252	0.06
22	40408928	40425814	16886	0.11
22	40575699	40595416	19717	0.06
22	40866848	40900306	33458	0.06
22	41104746	41108238	3492	0.11
22	42475109	42481995	6886	0.06
22	42561282	42569638	8356	0.06

22	43153990	43191484	37494	0.06
22	43236094	43250895	14801	0.29
22	43268338	43278939	10601	0.06
22	44798454	44802667	4213	0.06
22	44841806	44849050	7244	0.23
22	45368285	45394931	26646	0.17
22	45907443	45914050	6607	0.29
22	45916222	45935151	18929	0.46
22	46087068	46129298	42230	1.43
22	46238199	46240110	1911	0.06
22	46452922	46457033	4111	0.06
22	46663095	46690524	27429	0.06
22	48000033	48009083	9050	0.06
22	48039744	48041908	2164	0.06
22	48525853	48531736	5883	0.06
22	48610645	48611710	1065	0.06
22	48613648	48622839	9191	0.29
22	48741140	48769986	28846	0.86
22	48821286	48824177	2891	0.11
22	48893636	48897492	3856	0.06
22	48998097	49025325	27228	0.06
22	49076812	49081089	4277	0.23
22	49422634	49467384	44750	0.06

Chr: chromosome; bp: base pair

Human genome assembly: GRCh38

Supplementaty Table 2. Association of structural variations with asthma susceptibility in children from Salvador

Chr	Start	End	Freq. Case	Freq. Ctrl	OR	95% CI	P
Duplications							
1	154945225	155087375	2.1	0.8	2.5	0.9-6.5	0.06
2	132000728	132297217	9.8	6.7	1.5	0.9-2.3	0.08
2	227376905	227394586	1.6	2.1	0.8	0.3-1.9	0.6
3	75370585	75891946	6.1	5.9	1.0	0.6-1.6	0.9
3	95437291	95548133	2.9	2.0	1.7	0.8-3.7	0.2
3	164283490	164410087	2.4	2.4	1.2	0.53-2.5	0.7
4	31791952	31895086	1.1	1.5	0.8	0.2-2.3	0.6
4	68812961	68850046	2.1	0.7	3.2	1.2-8.5	**0.02**
4	174696163	174724266	1.9	1.3	1.5	0.6-3.6	0.4
5	84790666	84803174	1.3	1.5	1.0	0.4-2.8	0.9
6	34213903	34265377	2.9	1.3	2.1	0.9-4.7	0.06
7	26185559	26224658	1.3	2.3	0.6	0.2-1.5	0.2
7	62744672	63252790	4.0	3.1	1.4	0.7-2.6	0.3
11	1120324	1649253	5.9	5.0	1.2	0.7-2.1	0.4
11	2044100	2082296	1.1	1.3	0.7	0.2-2.1	0.5
11	50220667	50821348	7.7	5.2	1.9	1.1-3.4	**0.03**
11	65749835	65799248	4.0	3.9	1.0	0.6-1.9	0.9
11	65816789	65969132	4.8	3.4	1.4	0.8-2.4	0.3
12	11304779	11325747	1.6	1.1	1.2	0.5-2.9	0.7
13	36963500	36982862	1.9	1.4	1.3	0.5-3.2	0.6
14	100132248	100256325	3.2	2.6	1.2	0.6-2.5	0.5
16	87375682	87411805	2.7	1.0	2.6	1.1-6.1	**0.03**
17	14296292	14316169	2.4	0.9	2.8	1.1-6.8	**0.03**
19	3479373	3703130	4.5	4.6	0.9	0.5-1.7	0.9
19	3738651	3805890	2.1	2.7	0.8	0.4-1.8	0.6
19	8493358	8529661	1.1	1.7	0.6	0.2-1.8	0.4
19	15667349	15727059	6.1	3.7	1.8	1.1-3.1	**0.03**
19	32383819	32388358	2.4	1.0	2.5	1.0-6.2	**0.04**
19	36096969	36129876	1.3	1.2	0.9	0.3-2.7	0.9
19	48395295	48514469	3.2	1.5	1.9	0.9-4.0	0.09
20	60992315	61014216	1.9	1.4	1.3	0.5-3.2	0.5
Deletions							
1	1622382	1736859	0.3	1.3	0.2	0.02-1.4	0.1
1	22173037	22183413	1.3	1.3	1.3	0.5-3.7	0.6
1	61939375	61990763	0.5	1.3	0.4	0.09-1.7	0.2
1	103612312	103668424	1.6	1.2	1.4	0.5-3.7	0.5
1	105764986	105774609	1.3	1.2	1.2	0.4-3.1	0.8
1	161519677	161649951	4.0	4.5	0.8	0.5-1.5	0.5
1	196753917	196949015	2.1	2.9	0.7	0.3-1.5	0.4
1	210429364	210441720	0.8	1.5	0.5	0.1-1.7	0.3
2	4263430	4277582	3.5	2.4	1.4	0.7-2.8	0.3
2	35295108	35411515	1.1	1.0	0.9	0.3-3.1	0.9

2	57175857	57248779	1.9	1.5	1.1	0.5-2.7	0.8
2	67532940	67539817	1.9	1.3	1.4	0.6-3.5	0.5
2	112295484	112340863	1.3	1.1	1.2	0.4-3.5	0.7
2	125861332	125910808	1.3	1.2	1.5	0.4-2.9	0.9
2	127794853	127797562	1.3	1.2	1.2	0.4-3.5	0.7
2	163582547	163588672	2.1	1.2	1.8	0.8-4.2	0.2
2	183798045	184036734	2.1	3.1	0.7	0.3-1.5	0.3
2	184174332	184270717	3.2	1.4	2.4	1.1-5.1	**0.03**
2	217392920	217396646	1.3	1.3	1.1	0.4-3.2	0.8
3	106500281	106515006	1.6	1.6	0.9	0.3-2.2	0.8
3	159075161	159095191	1.6	1.8	0.9	0.4-2.4	0.9
4	3483685	3532851	1.3	2.4	0.5	0.2-1.3	0.2
4	14910729	14938665	6.1	3.1	1.9	1.1-3.3	**0.02**
4	39568333	39742743	0.0	1.4	-	-	-
4	45008827	45035653	3.7	2.9	1.2	0.6-2.2	0.7
4	68527638	68623755	2.7	3.1	0.9	0.4-1.8	0.7
4	111371861	111386488	2.1	3.1	0.7	0.3-1.5	0.4
4	143832994	144093819	2.4	4.5	0.5	0.2-1.0	0.06
4	186921989	186929178	2.1	1.8	1.2	0.5-2.7	0.7
5	661741	873886	2.1	3.0	0.7	0.3-1.6	0.4
5	79125957	79215563	2.7	3.4	0.7	0.3-1.4	0.3
5	97367538	98689505	2.4	1.5	1.8	0.8-3.9	0.1
5	113816844	113836766	1.6	1.4	1.1	0.4-2.8	0.9
5	177944712	177974508	2.4	0.7	4.6	1.7-12.2	**0.002**
6	26344105	26352025	3.5	3.4	0.9	0.5-1.9	0.9
6	29517512	29534259	0.5	1.2	0.4	0.09-1.7	0.2
6	29889788	29931412	6.6	2.4	2.9	1.7-5.2	**0.0002**
6	31387541	31488621	2.1	1.8	1.2	0.5-2.7	0.7
6	66238936	66726868	1.6	2.1	0.8	0.3-1.9	0.6
6	78149819	78467228	2.4	3.4	0.7	0.3-1.3	0.2
6	80575520	80584120	0.8	1.8	0.4	0.1-1.5	0.2
6	168040800	168093484	0.8	1.4	0.5	0.2-1.8	0.3
7	4315576	4495828	1.1	1.2	0.8	0.2-2.3	0.6
7	13068725	13090051	3.7	1.9	1.7	0.9-3.4	0.1
7	20267888	20288064	3.5	3.0	0.9	0.5-1.9	0.9
7	38256357	38338702	8.8	10.4	0.7	0.5-1.1	0.2
7	62742653	62997884	4.5	4.4	1.1	0.6-1.9	0.9
7	111140642	111443435	3.2	2.3	1.3	0.7-2.7	0.4
7	118487954	118530461	3.7	1.9	1.6	0.8-3.3	0.2
7	148934474	148947457	1.3	2.1	0.6	0.2-1.6	0.3
7	153497075	153509603	1.3	1.2	1.1	0.4-3.2	0.8
7	155093527	155097474	1.6	1.5	1.1	0.4-2.8	0.8
8	6101073	6126814	4.0	1.5	2.6	1.3-5.2	**0.009**
8	13652018	13756130	2.9	2.0	1.6	0.8-3.4	0.2
8	57442391	57454399	1.1	1.2	0.8	0.3-2.5	0.7
8	136673185	136849837	1.1	1.2	0.8	0.3-2.6	0.8

9	11640367	12182768	2.4	2.8	0.8	0.4-1.8	0.7
9	28672394	28776754	1.3	1.3	1.1	0.4-3.1	0.9
9	103005945	103023251	1.1	1.2	0.8	0.2-2.4	0.6
9	103954708	103958400	1.3	1.3	0.8	0.3-2.2	0.6
9	111702207	111707858	1.1	1.1	1.0	0.3-3.2	0.9
10	26395163	26402038	1.6	2.7	0.5	0.2-1.3	0.1
10	53167918	53177475	2.7	1.3	2.0	0.9-4.3	0.09
10	55099774	55361364	3.2	1.5	2.1	1.0-4.4	**0.05**
10	86653055	86663766	1.1	1.0	0.9	0.3-2.7	0.8
10	90237985	90242355	1.1	2.1	0.4	0.1-1.2	0.1
10	108543367	108557547	2.9	2.7	1.0	0.5-2.0	0.9
11	5844060	5869835	12.2	8.0	1.5	1.0-2.2	**0.03**
11	25564364	25909715	6.9	6.7	0.9	0.6-1.5	0.8
11	77177028	77208958	1.1	1.3	0.7	0.2-2.2	0.6
11	87675905	87743887	4.0	1.9	1.9	1.0-3.8	**0.05**
11	105053115	105060146	3.5	4.4	0.8	0.4-1.4	0.4
11	121999668	122030128	1.9	1.1	1.5	0.6-3.8	0.4
12	2136728	2150500	1.1	1.5	0.6	0.2-1.9	0.4
12	15415250	15421148	1.3	1.0	1.3	0.5-3.6	0.6
12	76100309	76136590	1.6	1.4	1.0	0.4-2.7	0.9
12	128072414	128093983	1.6	1.0	1.6	0.6-4.2	0.4
12	131245772	131340814	1.3	1.0	1.2	0.4-3.3	0.8
13	32097968	32099700	0.8	1.1	0.7	0.2-2.3	0.6
13	57239433	57387355	2.4	2.0	1.1	0.5-2.4	0.8
13	76036154	76039093	0.3	1.6	0.1	0.02-0.9	**0.04**
13	83527345	83582139	3.5	1.2	3.4	1.6-7.5	**0.002**
13	85285616	85308725	1.6	1.3	1.3	0.5-3.5	0.6
13	86684237	86934184	9.8	6.6	1.5	0.9-2.3	0.06
13	96268166	96274136	1.6	0.9	1.6	0.6-4.2	0.3
14	20215935	20275110	1.3	1.1	1.1	0.4-3.2	0.8
14	20877548	20952929	5.1	4.5	1.1	0.6-1.9	0.7
14	28105513	28483881	1.1	1.2	0.7	0.2-2.2	0.6
14	94945834	94953775	1.6	2.3	0.6	0.2-1.4	0.3
15	24112065	24518827	3.5	2.3	1.8	0.9-3.6	0.09
15	32216889	32327926	1.9	2.3	0.8	0.4-1.8	0.6
16	28591670	28618832	1.1	1.5	0.8	0.3-2.3	0.7
16	32457714	32638415	0.8	6.3	0.1	0.04-0.4	**0.0006**
16	34025437	34117342	3.2	3.0	1.1	0.5-2.1	0.9
16	76630598	76637010	3.7	2.0	2.0	1.0-3.9	**0.04**
17	19590814	19634157	3.5	4.1	0.9	0.5-1.6	0.7
17	73750237	73752292	0.5	1.6	0.3	0.07-1.4	0.1
18	4329893	4335067	2.1	0.8	2.2	0.8-5.6	0.1
18	5926003	5934150	1.6	1.2	1.3	0.5-3.3	0.6
18	67629278	67651557	2.9	4.0	0.7	0.4-1.4	0.4
19	20517745	20774347	4.8	5.3	0.8	0.5-1.4	0.5
19	20973591	20983030	2.7	1.5	1.6	0.7-3.4	0.2

19	30826297	30827451	3.2	2.3	1.3	0.6-2.7	0.4
19	42739884	43344645	4.3	5.0	0.9	0.5-1.6	0.6
20	16095377	16103137	1.1	1.5	0.7	0.2-2.0	0.5
20	23187139	23194046	1.6	1.9	0.8	0.3-2.0	0.7
21	22951909	23082655	1.6	0.9	1.7	0.6-4.5	0.3
21	37305994	37341883	0.8	1.2	0.6	0.2-1.9	0.4
21	44391460	44414033	0.8	1.3	0.5	0.1-1.6	0.2
21	45975479	46011117	0.5	1.2	0.4	0.1-1.9	0.3
22	25268027	25532662	0.8	1.2	0.6	0.2-2.2	0.4
22	34543323	34585637	1.9	2.0	0.8	0.3-1.9	0.7
22	38968338	38996475	3.5	1.8	1.9	0.9-3.8	0.07
22	46087068	46129298	0.5	1.7	0.3	0.07-1.3	0.1

Chr: Chromosome; Coordinates in base pair; Frequency of CNVR (%); OR, odds ratio; 95% CI, 95% confidence interval; p, p value (additive model)

Covariates in multivariate analysis: sex, age, Log_2 of R Ratio standard deviation (LRRSD) and principal components (PC1, PC2 and PC3)

Human genome assembly: GRCh38

Supplementary Table 3. The deletion at 6p22.1 is nominally associated with asthma in individuals with different ancestry compositions

CNVR	EUR (%) median	Freq. Case	Freq. Ctrl	OR	SE	95% CI	p
**Salvador, Brazil**	> 42	0.068	0.028	2.6	0.4	1.3-5.4	**0.01**
del 6:29,889,788-29,931,412	≤ 42	0.059	0.017	3.8	0.4	1.6-8.9	**0.002**
**Pelotas, Brazil**	> 84	0.049	0.026	1.8	0.3	1.2-3.0	**0.03**
del 6:29,881,842-29,931,412	≤ 84	0.034	0.018	1.8	0.3	1.1-3.4	**0.04**

CNVR: Copy number variation region, chromosome:start-end; EUR (%) median: European ancestry composition (ADMIXTURE method); Frequency of CNVR (%); Case: asthmatic; Ctrl: non-asthmatic; OR, odds ratio; SE, SE of odds ratio (OR); 95% CI, 95% confidence interval; p, p value (additive model)

Covariates in multivariate analysis: sex, age, Log_2 of R Ratio standard deviation (LRRSD) and principal components (PC1, PC2 and PC3)

Human genome assembly: GRCh38
